# *ZNF445*: a homozygous truncating variant in a patient with Temple syndrome and multilocus imprinting disturbance

**DOI:** 10.1186/s13148-021-01106-5

**Published:** 2021-05-26

**Authors:** Masayo Kagami, Kaori Hara-Isono, Keiko Matsubara, Kazuhiko Nakabayashi, Satoshi Narumi, Maki Fukami, Yumiko Ohkubo, Hirotomo Saitsu, Shuji Takada, Tsutomu Ogata

**Affiliations:** 1 Department of Molecular Endocrinology, National Research Institute for Child Health and Development, 2-10-1 Okura, Setagaya-ku, Tokyo, 157-8535 Japan; 2 Department of Maternal Fetal Biology, National Research Institute for Child Health and Development, 2-10-1 Okura, Setagaya-ku, Tokyo, 157-8535 Japan; 3 Department of Pediatrics, Shizuoka Saiseikai Hospital, Oshika 1-1-1, Suruga-ku, Shizuoka, 422-8527 Japan; 4 Department of Biochemistry, Hamamatsu University School of Medicine, 1-20-1 Handayama, Higashi-ku, Hamamatsu, 431-3192 Japan; 5 Department of Systems BioMedicine, National Research Institute for Child Health and Development, 2-10-1 Okura, Setagaya-ku, Tokyo, 157-8535 Japan; 6 Department of Pediatrics, Hamamatsu University School of Medicine, 1-20-1 Handayama, Higashi-ku, Hamamatsu, 431-3192 Japan; 7 Department of Pediatrics, Hamamatsu Medical Center, Tomitsuka 328, Naka-ku, Hamamatsu, 432-8580 Japan

**Keywords:** *ZNF445*, Postzygotic genomic imprint, Multilocus imprinting disturbance, Temple syndrome, *MEG3/DLK1*:IG-DMR

## Abstract

**Background:**

*ZNF445*, as well as *ZFP57*, is involved in the postfertilization methylation maintenance of multiple imprinting-associated differentially methylated regions (iDMRs). Thus, *ZNF445* pathogenic variants are predicted to cause multilocus imprinting disturbances (MLIDs), as do *ZFP57* pathogenic variants. In particular, the *MEG3/DLK1*:IG-DMR would be affected, because the postzygotic methylation imprint of the *MEG3/DLK1*:IG-DMR is maintained primarily by ZNF445, whereas that of most iDMRs is preserved by both ZFP57 and ZNF445 or primarily by ZFP57.

**Results:**

We searched for a *ZNF445* variant(s) in six patients with various imprinting disorders (IDs) caused by epimutations and MLIDs revealed by pyrosequencing for nine iDMRs, without a selection for the original IDs. Re-analysis of the previously obtained whole exome sequencing data identified a homozygous *ZNF445* variant (NM_181489.6:c.2803C>T:p.(Gln935*)) producing a truncated protein missing two of 14 zinc finger domains in a patient with Temple syndrome and MLID. In this patient, array-based genomewide methylation analysis revealed severe hypomethylation of most CpGs at the *MEG3*:TSS-DMR, moderate hypomethylation of roughly two-thirds of CpGs at the *H19/IGF2*:IG-DMR, and mild-to-moderate hypomethylation of a few CpGs at the *DIRAS3*:TSS-DMR, *MEST*:alt-TSS-DMR, *IGF2*:Ex9-DMR, *IGF2*:alt-TSS, and *GNAS*-*AS1*:TSS-DMR. Furthermore, bisulfite sequencing analysis for the *MEG3/DLK1*:IG-DMR delineated a markedly hypomethylated segment (CG-A). The heterozygous parents were clinically normal and had virtually no aberrant methylation pattern.

**Conclusions:**

We identified a *ZNF445* pathogenic variant for the first time. Since ZNF445 binds to the *MEG3/DLK1*:IG-DMR and other iDMRs affected in this patient, the development of Temple syndrome and MLID would primarily be explained by the *ZNF445* variant. Furthermore, CG-A may be the target site for ZNF445 within the *MEG3/DLK1*:IG-DMR.

**Supplementary Information:**

The online version contains supplementary material available at 10.1186/s13148-021-01106-5.

## Background

Recent studies have identified multilocus imprinting disturbances (MLIDs) in a subset of patients with imprinting disorders (IDs) caused by epimutations affecting imprinting-associated differentially methylated regions (iDMRs) [1]. Although underlying factor(s) for MLIDs remains largely unknown, MLID-related genetic variants have been identified in several genes involved in the establishment of genomic imprints in the oocyte (*e.g.*, *NLRP7* [2]) and in the maintenance of postzygotic genomic imprints (e.g., *ZFP57* [3]).

**Fig. 1:**
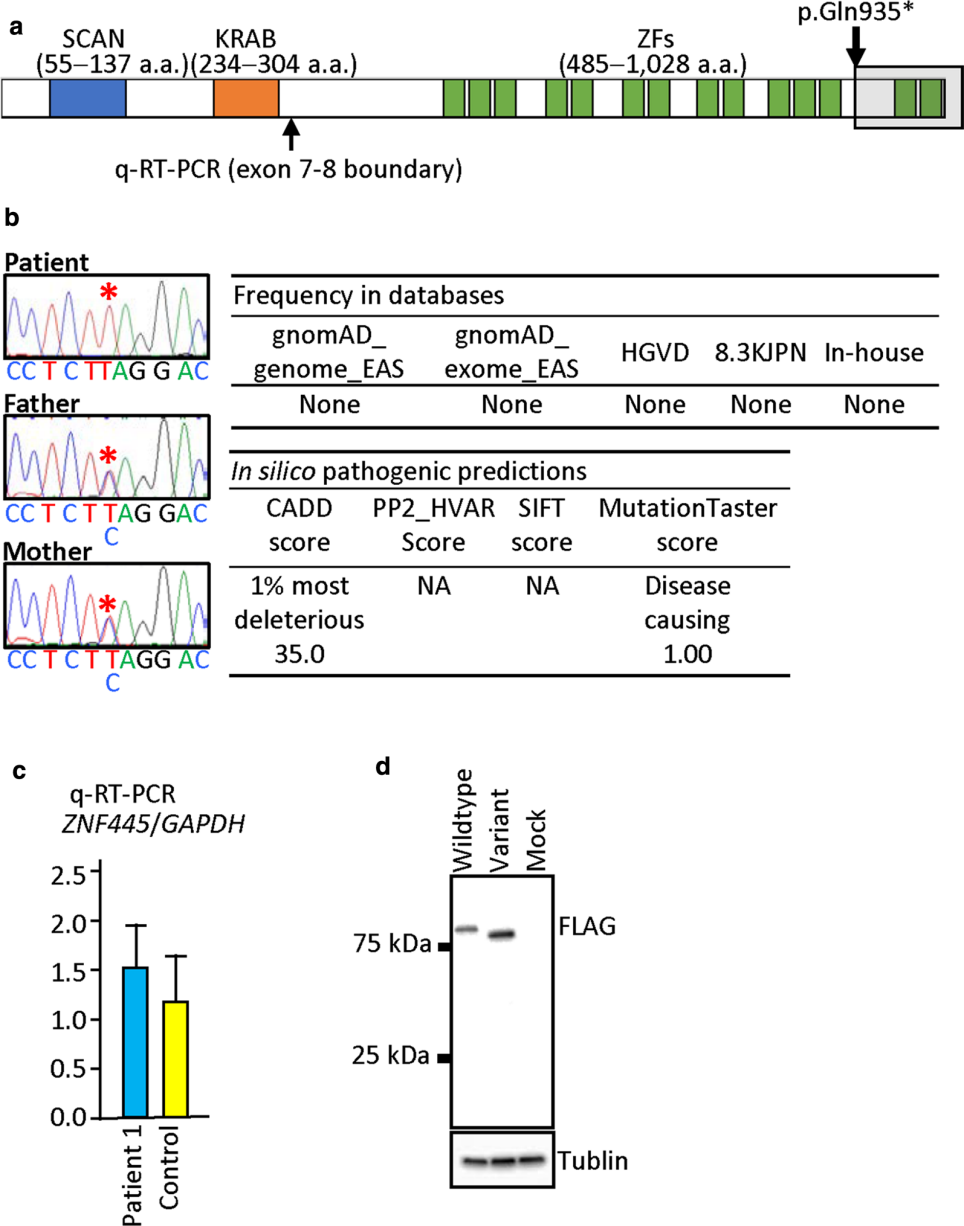
*ZNF445* truncating variant identified in patient 1. **a** Structure of the ZNF445 protein and the position of the c.2803C>T (p.Gln935*) variant. The variant is predicted to produce a truncated ZNF445 protein missing two ZF domains. SCAN: SRE-ZBP, CTfin51, AW-1 and Number 18 cDNA; KRAB: Krüppel-associated box; and ZF: zinc finger. **b** Electrochromatograms showing homozygosity for the variant in patient 1 and heterozygosity for the variant in the parents (red asterisks). This variant is completely absent from public and in-house databases utilized in this study and is predicted to have high pathogenicity. **c** Quantitative reverse-transcriptase PCR analysis by the Taqman methods (ThermoFisher Scientific), using probe for *ZNF445* (Hs00738798,) and *GAPDH* (4326317E). **d** Western blot findings showing the production of a truncated ZNF445 protein by a vector with variant *ZNF445* cDNA (c.2803C>T)

Recently, Takahashi et al. have reported that *ZNF445*, as well as *ZFP57*, plays a critical role in the maintenance of postfertilization methylation imprints [4]. *ZNF445* consists of eight exons and encodes a 1,031 amino acid protein. ZNF445 belongs to the KRAB-containing KZFP family and carries a SCAN domain and 14 zinc finger (ZF) domains together with the KRAB domain (Fig. 1a). ZNF445 binds to multiple imprinting control regions (ICRs) within iDMRs and maintains methylation imprints by recruiting KRAB-associated protein 1 (KAP1) (alias, TRIM28) [4]. Thus, ZNF445 has a biological function similar to that of ZFP57, which also belongs to the KZFP family [4, 5]. It is predicted, therefore, that *ZNF445* pathogenic variants lead to MLIDs, as do *ZFP57* pathogenic variants [3]. In particular, the *MEG3/DLK1*:IG-DMR would be affected, because (1) chromatin immunoprecipitation (ChIP) analyses using human embryonic stem (hES) cells or human embryonic kidney 293 T (HEK293T) cells have shown that the *MEG3/DLK1*:IG-DMR is associated primarily with ZNF445 binding, while most iDMRs are associated with both ZFP57 and ZNF445 bindings or primarily with ZFP57 binding [4, 5]; and (2) the *MEG3/DLK1*:IG-DMR is hypomethylated in *ZNF445* knockdown hES cells [4].

Here we report the first *ZNF445* pathogenic variant identified in a patient with Temple syndrome (TS14) and MLID.

## Results

### Patients

We examined six patients with IDs caused by epimutations, i.e., patient 1 with TS14, patient 2 with both Beckwith–Wiedemann syndrome (BWS) and pseudohypoparathyroidism type Ib (PHP-Ib), patients 3 and 5 with Silver–Russell syndrome (SRS), and patients 4 and 6 with BWS, who were found to have MLIDs by pyrosequencing analysis for nine IDs-related iDMRs (Additional file 1: Table S1). Patients 1 and 2 have been reported previously [6, 7]. Uniparental disomies and deletions/duplications involving the IDs-related iDMRs were excluded by microsatellite analysis and custom-build oligo-microarray analysis for iDMRs, respectively. Here, since it was uncertain whether *ZNF445* pathogenic variants could be identified in epimutation- and MLID-positive patients with TS14 or in those with other IDs, we studied all the six patients in whom whole exome sequencing (WES) has already been performed (patients 1 and 2 and their parents) [6, 7] or DNA samples and informed consent for WES have been obtained (patients 3–6 and the parents of patients 3–5; the parents of patient 6 were not examined), with no selection for the original IDs.

### Identification of a *ZNF445* truncating variant

We re-analyzed the previously obtained WES data in patients 1 and 2 and their parents, and executed WES in patients 3–6 and the parents of patients 3–5 in this study. Consequently, we revealed a nonsense variant on the last exon 8 of *ZNF445* (NM_181489.6:c.2803C > T:p.(Gln935*)) in patient 1 (Fig. 1a). This variant was present in a homozygous condition in patient 1 and in a heterozygous condition in the parents (Fig. 1b and Additional file 6: Figure S1). The variant was completely absent from public and in-house databases utilized in this study and was assessed to have high pathogenicity. Consistent with the notion that a premature termination on the last exon does not cause nonsense mediated mRNA decay [8], quantitative RT-PCR for lymphoblastoid cell lines showed a similar *ZNF445* expression dosage between patient 1 and control subjects (Fig. 1c), and western blot analysis revealed a truncated ZNF445 protein consistent with loss of two of 14 ZF domains in patient 1 (Fig. 1d). While the parents had the same rare variant, PLINK analysis (https://www.cog-genomics.org/plink/1.9/) [9] argued against consanguinity between the parents (Additional file 2: Table S2). Unfortunately, we could not examine the paternal and maternal grandparents, because of their refusal to receive molecular studies.

No other rare (minor allele frequency ≤ 0.01) variant with high predicted pathogenicity (CADD-PHRED score > 20) was identified in *ZNF445* of patients 2–6 and the parents of patients 2–5 as well as in other causative or candidate oocyte and zygotic factor genes for MLIDs such as *NLRP2, NLRP7, KHDC3L, NLRP5, TRIM28, PADI6, OOEP, UHRF1, ZAR1*, and *ZFP57* [1–3, 10, 11] of patients 1–6 and the parents of patients 1–5.

### Genomewide methylation analysis

Genomewide methylation analysis was performed using Infinium MethylationEPIC Kit (EPIC) (Illumina), covering > 850,000 CpGs in the genome. The selection of CpGs to be examined and the definitions of abnormally methylated CpGs and aberrantly methylated iDMRs were described in "Methods".

**Fig. 2:**
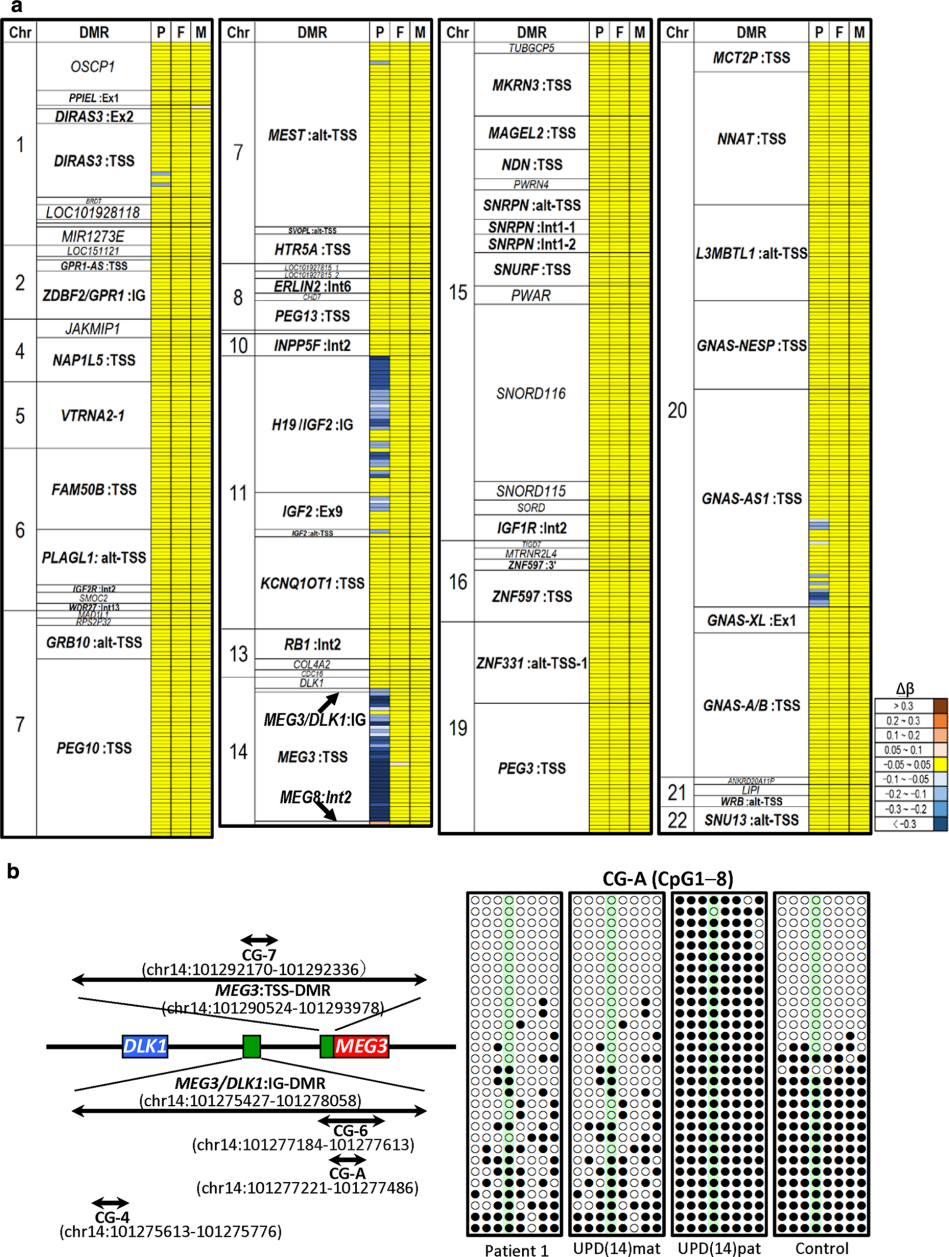
Methylation analysis. **a** Heatmap indicating the Δβ values for 855 CpGs on 78 reported iDMRs examined by EPIC. The methylation levels of CpG sites are classified into nine categories on the basis of Δβ values. A single row indicates a single probe (CpG site). P, patient 1; F, father; and M, mother. **b** Bisulfite sequencing for CG-A. Left part shows the genomic positions of CG-A, CG-4, and CG-6 within the *MEG3*/*DLK1*:IG-DMR and CG-7 within the *MEG3*:TSS-DMR, based on GRCh37/hg19. Right part shows the bisulfite sequencing data for CG-A. Each line indicates each clone, and filled and open circles represent methylated and unmethylated cytosines at the CpG dinucleotides, respectively. The fourth CpGs highlighted in light green have also been examined by EPIC. Bisulfite sequencing data for CG-4 and CG-7 in patient 1 have been reported previously (Fig. 2C in Kagami et al. [6])

The methylation analysis for 855 CpGs on 78 iDMRs revealed severe hypomethylation of most CpGs at the *MEG3*:TSS-DMR, moderate hypomethylation of roughly two-thirds of CpGs at the *H19/IGF2*:IG-DMR, mild-to-moderate hypomethylation of several CpGs at the *GNAS-AS1*:TSS-DMR, and mild hypomethylation of a single to few CpGs at the *DIRAS3*:TSS-DMR, *MEST*:alt-TSS-DMR, *IGF2*:Ex9-DMR, and *IGF2*:alt-TSS-DMR, together with a mildly hypomethylated single CpG at the *MEG3*/*DLK1*:IG-DMR and a mildly hypermethylated single CpG at the *MEG8*:Int2-DMR in patient 1 (Fig. 2a and Additional file 3: Table S3). The *MEG3*:TSS-DMR, *H19/IGF2*:IG-DMR, *IGF2*:Ex9-DMR, and *GNAS-AS1*:TSS-DMR were regarded as aberrantly methylated iDMRs (Fig. 2a and Additional file 3: Table S3). The hypomethylated CpGs tended to be clustered within the affected iDMRs. The parents had no aberrantly methylated CpG, except for a single mildly hypermethylated CpG at the *MEG3*:TSS-DMR in the father and a single mildly hypermethylated CpG at the *C1orf177-*DMR in the mother.

By contrast, the methylation analysis for 333,868 autosomal CpGs at CpG islands and their north and south shores and shelves showed a comparable distribution of β-values indicating the methylation levels among patient 1, the parents, and control subjects, as illustrated with violin plots (Additional file 7: Figure S2). Indeed, only 24 CpGs were found to be abnormally methylated on non-iDMRs of patient 1 (Additional file 4: Table S4).

### Methylation analysis for the *MEG3*/*DLK1*:IG-DMR

Since the *MEG3*/*DLK1*:IG-DMR was barely examined by EPIC, bisulfite sequencing was carried out for CG-A within the previously identified DMR segment named CG-6 (Fig. 2b and Additional file 8: Figure S3). As a whole, CG-A was markedly hypomethylated in patient 1 as well as in a upd(14)mat case, although a few CpGs including the single CpG examined by EPIC were less severely hypomethylated.

### Clinical report

Detailed clinical findings of patient 1 up to 4 5/12 years have been reported previously [6]. In short, she was conceived naturally and delivered by a caesarean section because of fetal growth arrest at 34 weeks of gestation. She exhibited all the six Netchine–Harbison clinical diagnostic features for SRS: (1) small for gestational age (birth length, – 2.9 SD; and birth weight, – 3.8 SD), (2) postnatal growth failure (height at 24 months, – 4.5 SD); (3) relative macrocephaly at birth (occipitofrontal circumference SDS at birth, + 2.5 above birth length SDS and + 3.4 above birth weight SDS); (4) protruding forehead during 1–3 years; (5) body asymmetry; and (6) feeding difficulties requiring tube feeding [12]. She also manifested salient clinical features prompting genetic testing for Prader−Willi syndrome (PWS) before 6 years of age, such as severe hypotonia with poor suck and global developmental delay [13]. On the last examination at 5 9/12 years of age, she showed growth failure (height, – 4.0 SD; weight, – 6.0 SD; and occipitofrontal circumference, – 1.9 SD) despite growth hormone treatment from 3.0 years of age.

The parents were clinically healthy, with the paternal height of 164 cm (–1.2 SD) and the maternal height of 150 cm (–1.6 SD). Allegedly, the paternal and maternal grandparents were also healthy, and there was no consanguinity.

## Discussion

We identified a homozygous nonsense variant of *ZNF445* in patient 1. This variant was completely absent from the public and in-house databases utilized in this study and was shown to produce a truncated ZNF445 protein missing two ZF domains. Furthermore, it is notable that (1) *ZNF445* is associated with a high probability of being loss-of-function intolerant score (1.0) [14], and (2) no homozygous loss-of-function variant has been registered in the gnomAD database (https://gnomad.broadinstitute.org/v2.1.1), although heterozygous loss-of-function variants have been recorded. These findings, together with the normal phenotype and the nearly complete lack of aberrant methylation pattern in the heterozygous parents of patient 1, suggest that *ZNF445* has an essential biological function in the human and that biallelic rather than monoallelic variants of *ZNF445* can lead to disease phenotype. Taken together, it is likely that the *ZNF445* truncating variant is a disease-causing variant with a recessive effect.

Patient 1 had TS14-compatible severe hypomethylations affecting the *MEG3*/*DLK1*:IG-DMR and *MEG3*:TSS-DMR and mild-to-moderate MLID affecting several iDMRs, in the presence of the *ZNF445* variant. In this regard, previous studies have shown that: (1) in hES cells or HEK293T cells, ZNF445 binds to multiple iDMRs including the iDMRs affected in patient 1, to maintain the postfertilization imprint [4], (2) while the postzygotic methylation imprint of most iDMRs is preserved by both ZFP57 and ZNF445 or primarily by ZFP57, that of the *MEG3/DLK1*:IG-DMR is maintained primarily by ZNF445 [4, 5]; and (3) the *MEG3/DLK1*:IG-DMR functions as a hierarchically upper regulator for the methylation pattern of the *MEG3*:TSS-DMR, so that the hypomethylated *MEG3/DLK1*:IG-DMR renders the *MEG3*:TSS-DMR hypomethylated [15]. These findings would explain, in terms of a defective ZNF445 function, why patient 1 had the severely hypomethylated *MEG3/DLK1*:IG-DMR (CG-A) and *MEG3*:TSS-DMR and the mild-to-moderate degrees of MLID. This notion would be supported by the development of severe hypomethylation of the *PLAGL1*:alt-TSS-DMR leading to transient neonatal diabetes mellitus and MLID in patients with *ZFP57* pathogenic variants [3], because the postzygotic methylation imprint of the *PLAGL1*:alt-TSS-DMR is preserved primarily by ZFP57 [4, 5].

For the *MEG3/DLK1*:IG-DMR, the CG-A within CG-6 was severely hypomethylated in patient 1 as well as in a upd(14)mat case. In this regard, the *MEG3/DLK1*:IG-DMR is known to harbor at least two DMR segments (CG-4 and CG-6) (Fig. 2B and Additional file 8: Figure S3) [15], and the previous bisulfite sequencing and pyrosequencing analyses have shown that CG4 in patient 1 is irregularly (not differentially) methylated and, consequently, associated with apparently normal methylation indices (Additional file 1: Table S1 and Fig. 2A and 2C in Kagami et al. [6]). It is possible, therefore, that CG-A contains the major target sequence such as the ICR for ZNF445 and functions as a hierarchically upper regulator for the methylation pattern of the *MEG3*:TSS-DMR [15]. However, both CG-A and CG-4 reside within the regions harboring ZNF445 binding sites identified by ChIP analysis using hES cells [4] (Additional file 8: Figure S3), and further studies are required to determine the precise ZNF445 binding site(s) with a biological function within the *MEG3/DLK1*:IG-DMR. In this context, the segment with continuously hypomethylated CpGs within the *H19*/*IGF2*:IG-DMR may harbor the major target sequence for ZNF445.

The human *ZNF445* expression is strong in the oocyte and the 2–4 cell embryos and becomes weak afterwards [5]. This may suggest that the methylation defects of patient 1 primarily occurred around the time of the high *ZNF445* expression. In addition, since human *ZFP57* expression remains nearly undetectable in the oocyte and the 2–8 cell embryos and becomes obvious in the inner cell mass [5], the deleterious effects of *ZNF445* deficiency in the oocyte and the 2–4 cell embryos could not be compensated for by the *ZFP57* function. However, it is unlikely that the maternal *ZNF445* variant in the oocyte is responsible for the development of methylation defects of patient 1. Although the *MEG3/DLK1*:IG-DMR, *MEG3*:TSS-DMR, and *H19/IGF2*:IG-DMR were obviously hypomethylated in patient 1, these iDMRs are methylated in the sperm, not in the oocyte [16]. By contrast, it may be possible that the homozygous *ZNF445* variant at the initial postzygotic stage is relevant to the development of methylation defects in patient 1. In this regard, while the methylation disturbance occurring at such an initial stage is assumed to lead to relatively severe methylation defects at multiple loci, most of the ZNF445 bound iDMRs in HEK293T cells, such as the *DIRAS3*:TSS-DMR, *ZDBF2*/*GPR1*:IG-DMR, *MEST*:alt-TSS-DMR, *PEG13*:TSS-DMR, *KCNQ1OT1*:TSS-DMR, *NNAT:*TSS-DMR, *GNAS-NESP*:TSS-DMR, *GNAS-AS1*:TSS-DMR, *GNAS-XL*:Ex1-DMR, and *SNU13*:alt-TSS-DMR [5], were barely affected in patient 1. However, it has been suggested for the maternal effect gene *NLRP5* that although the concepti of variant positive mothers have broad methylation defects, natural epigenetic resetting takes place during the embryonic development, leaving only a few methylation defects detectable postnatally [17]. If such an epigenetic resetting has occurred after broad methylation defects at the initial postzygotic stage in patient 1, this would lead to the development of MLID associated with severe hypomethylation of the *MEG3/DLK1*:IG-DMR (CG-A), whose postzygotic methylation imprint is primarily maintained by ZNF445 [4, 5], and resultant marked hypomethylation of the *MEG3*:TSS-DMR [15], together with mild-to-moderate hypomethylation at several loci. Notably, the broad methylation defects at the initial postzygotic period, if it has indeed occurred in patient 1, could be due to the maternal effect of the *ZNF445* variant in the fertilized ovum. Thus, the *ZNF445* variant may have behaved not only as a zygotic factor leading to the methylation defects in a locus-dependent manner, but also as an oocyte factor, resulting in the initial broad methylation defects [2, 4, 5, 11].

Previous studies in patients with MLIDs have revealed no pathogenic *ZNF445* variant [5], although they have identified multiple pathogenic *ZFP5*7 variants [3]. In this regard, *ZNF445* is expressed in the oocyte and initial postzygotic stage, whereas *ZFP57* is expressed from a relatively later postzygotic stage [4, 5]. Thus, while even biallelic apparently amorphic variants of *ZFP57* have been reported [3], biallelic severe loss-of-function variants of *ZNF445* may cause embryonic lethality, because of the disrupted methylation maintenance from a very early developmental stage. Thus, the truncating variant in patient 1 would be a hypomorphic rather than amorphic variant. One may argue against the lethality of *ZNF445* biallelic amorphic variants, because mice carrying either zygotic or maternal-zygotic *Znf445* deletions have no discernible methylation defects in the brain at 12.5 embryonic days, and one-third of such mice survive to adulthood [4]. However, in contrast to human *ZNF445* and *ZFP57* expression patterns, mouse *Znf445* and *Zfp57* are continuously expressed from the zygote to early blastocyst stage, with the expression level being higher in *Zfp57* than in *Znf445* [4]. It is likely, therefore, that the deleterious effects of *Znf445* deficiency could be compensated for by the high *Zfp57* expression in the mouse [4]. In support of the compensatory effects between *Zfp57* and *Znf445*, the *Zfp57* and *Znf445* double knockout mice have massive methylation defects and nearly lethal in the embryonic life [4]. Furthermore, previous studies in search of a *ZNF445* variant(s) have been performed for MLID-positive patients diagnosed with transient neonatal diabetes mellitus (n = 3), BWS (n = 28), and PHP-Ib (n = 10) [5]. Considering the critical role of ZNF445 in the methylation maintenance of the *MEG3/DLK1*:IG-DMR, *ZNF445* variants may be hidden in patients with TS14 caused by epimutations. Consistent with this, a ZNF445 variant was not identified in patients 2–6 with IDs other than TS14. Thus, we are planning to perform *ZNF445* analysis and methylation profiling in patients with TS14 caused by epimutations.

Several matters should be pointed out in this study. First, patient 1 was diagnosed with TS14, because of the marked hypomethylation of the *MEG3/DLK1*:IG-DMR and *MEG3*:TSS-DMR. Indeed, the coexistence of SRS- and PWS-compatible phenotypes is characteristic of TS14 in infancy to early childhood and is observed in ~ half of TS14 patients at that period [18]. Furthermore, it is likely that the moderately hypomethylated *H19*/*IGF2*:IG-DMR has also contributed to the development of typical SRS phenotype in patient 1 [12], as have been implicated previously [6]. Second, no pathogenic variant was identified in the MLID-related oocyte and zygotic factor genes of patients 2–6 and the parents of patients 2–5. This may imply a relatively small relevance of genetic variants to MLIDs, while it is possible that a pathogenic variant(s) is hidden in noncoding regions and that several genes for MLIDs remain undetected at present. Third, the parents were heterozygotes for the extremely rare *ZNF445* variant. Thus, although the PLINK analysis argued against consanguinity, they might be very distant relatives. Fourth, the degree of hypomethylation was more obvious for most of the abnormally methylated CpGs in the EPIC analysis than in than HumanMethylation450 BeadChip utilized in the previous study (Fig. 2B in Kagami et al. [6]), although the overall methylation pattern was similar between the two methods. This would primarily be due to the difference in the DNA samples of patient 1 (obtained at 3 years of age in the previous study and at 5 years of age in this study) and in the control subjects employed in the two methods. Fifth, the single CpG at the *MEG8*:Int2-DMR was hypermethylated. This is consistent with the previous finding that this DMR shows a methylation pattern opposite to that of the *MEG3*:TSS-DMR and *MEG3/DLK1*:IG-DMR [19]. Lastly, the distribution of the β-values for autosomal CpGs at and around CpG islands was comparable among patient 1, the parents, and control subjects. This would imply that ZNF445 mainly, if not exclusively, functions at ICRs within iDMRs.

## Conclusions

We identified for the first time a homozygous variant of *ZNF445* in a patient with TS14 and MLID. Further studies will permit to clarify the role of *ZNF445* variants in the development of MLIDs.

## Methods

### Samples and primers

Molecular studies were performed for leukocyte genomic DNA (gDNA) samples. Primers utilized are shown in Additional file 5: Table S5.

### Whole exome sequencing (WES)

In patients 1 and 2, we searched for a variant(s) of *ZNF445* and other recently reported MLID-related genes, using the previously obtained WES data [6, 7]. In patients 3–6 and the parents of patients 3–5, we performed WES in this study. In brief, WES was carried out using SureSelect Human All Exon V6 (Agilent Technologies), and captured libraries were sequenced by NextSeq 500 (Illumina) with 150-bp paired-end reads. Exome data processing, variant calling, and variant annotation were carried out, as described previously [20]. Human GRCh37/h19 (https://genome.ucsc.edu) was utilized as the reference genome, and NM_181489.6 (https://www.ncbi.nlm.nih.gov/genbank) as the reference for a *ZNF445* variant.

We extracted rare variants with minor allele frequencies of ≤ 0.01 in all the following public databases and in-house database (n = 218): (1) Whole genome and exome data for East Asian Population in Genome Aggregation Database (gnomAD_genome_EAS & gnomAD_exome_EAS) (http://gnomad.broadinstitute.org/); (2) Human Genetic Variation Database (HGVD) (http://www.hgvd.genome.med.kyoto-u.ac.jp/); and (3) whole genome sequences of 8380 healthy Japanese individuals and construction of the highly accurate Japanese population reference panel (8.3KJPN) (https://ijgvd.megabank.tohoku.ac.jp/). Final variants were annotated with Annovar [21].

In silico pathogenicity predictions were carried out for identified rare variants, using the following methods: (1) CADD (Combined Annotation–Dependent Depletion) (http://cadd.gs.washington.edu/); (2) PP2_HVAR (Polyphen-2 Hum Var) (http://genetics.bwh.harvard.edu/pph2/); (3) SIFT (Sorting Intolerant From Tolerant) (http://sift.jcvi.org/); and (4) MutationTaster (http://www.mutationtaster.org/).

Exome sequencing data were also utilized to examine an exonic deletion(s) by exome sequencing-based CNV calling [22] and consanguinity by PLINK [9].

### Western Blotting

We introduced human *ZNF445* cDNA, wildtype or p.Gln935* created by mutagenesis, into pcDNA3 vector with 3xFLAG-tag using the Gibson assembly technique (New England Biolabs, Ipswich, MA, USA). The vectors were transfected into HEK293 cells using Lipofectamine 3000 (ThermoFisher Scientific), and cell lysates were prepared from transfected cells maintained for 48 h. Western blotting was performed with anti-FLAG M2 antibody (Sigma-Aldrich) and anti-tubulin antibody (Abcam) as primary antibodies and with anti-Mouse IgG and anti-Rat IgG (Sigma-Aldrich) as secondary antibodies.

### Genomewide methylation analysis

Genomewide methylation analysis was performed, using Infinium MethylationEPIC Kit (EPIC) (Illumina) covering > 850,000 CpGs in the genome. In brief, 500 ng of bisulfite-treated gDNA was subjected to the beadchip and was scanned using the Illumina iScan system. Then, we applied background subtraction and, subsequently, discarded CpGs with the detection *P *values of > 0.01 and/or no signal intensities, as well as those on the sex chromosomes. Data import, quality control, and correction of multiple batch effect among arrays were performed by R version 3.4.1 using ChAMP R package (version 2.8.9).

We obtained *β*-values indicating the methylation levels for 855 CpGs on 78 iDMRs, after excluding CpGs showing age-related drift and sex bias [23–26]. We calculated the mean and SD of *β*-value at each CpG in 24 control subjects and obtained the difference between the β-value of each case (patient 1, the father, and the mother) and the mean β-value of the control group (Δ*β*). The methylation level of each CpG (probe) was interpreted as abnormal, when the |*β*| was > 3 SD of the mean in controls and |Δ*β*| was > 0.05. When at least two consecutive probes with abnormally methylated levels were detected within iDMRs harboring at least four examined probes, such iDMRs were defined as aberrantly methylated iDMR [27].

We further obtained *β*-values indicating the methylation levels for 333,868 autosomal CpGs at CpG islands based on the UCSC criteria and their north and south shores and shelves, and drew violin plots using the data. To identify abnormally methylated CpGs on non-iDMRs, we took the following steps: (1) exclusion of CpGs in which the values for the mean + 3 SD were above 1.0 or those for the mean − 3 SD were below 0; (2) extraction of CpGs with the |β| of > 3 SD of the mean in controls and |Δβ| of > 0.05; (3) exclusion of CpGs on the iDMR; and (4) exclusion of CpGs showing age-related drift and sex bias [23–26].

### Other experiments

Other experiments were carried out by the standard methods.

## Supplementary Information

**Additional file 1: Table S1.** Methylation indices (%) for CpGs determined by pyrosequencing analysis for disease-related iDMRs.

**Additional file 2: Table S2**. Assessment of consanguinity based on PI_HAT values calculated by PLINK.

**Additional file 3: Table S3.** Methylation values of 855 probes (CpGs) on differentially methylated regions, measured by Infinium MethylationEPIC Kit.

**Additional file 4: Table S4**. The list of abnormally methylated 24 probes (CpGs) on non-iDMRs.

**Additional file 5: Table S5** Primers utilized in the present study.

**Additional file 6: Figure S1.** Exclusion of a heterozygous deletion involving *ZNF445* in patient 1.

**Additional file 7: Figure S2**. Violin plots of the β-values for autosomal probes in patient 1, the parents, and 24 control subjects (average).

**Additional file 8: Figure S3**. Physical map of the chromosome 14q32.2 imprinted region and that of the magnified *MEG3/DLK1*:IG-DMR and *MEG3*:TSS-DMR.

## Data Availability

All data generated or analyzed during this study are available from the corresponding author on reasonable request.

## Abbreviations

iDMR: Imprinting-associated DMR
MLID: Multilocus imprinting disturbance
ID: Imprinting disease
ZF: Zinc finger
ICR: Imprinting control region
KAP1: KRAB-associated protein 1
ChIP: Chromatin immunoprecipitation
hES: Human embryonic stem
HEK293T: Human embryonic kidney 293 T
TS14: Temple syndrome
BWS: Beckwith–Wiedemann syndrome
PHP-Ib: Pseudohypoparathyroidism type Ib
SRS: Silver–Russell syndrome
WES: Whole exome sequencing
EPIC: Infinium MethylationEPIC Kit
PWS: Prader–Willi syndrome
