# Supplementary material for: ZNF445: a homozygous truncating variant in a patient with Temple syndrome and multilocus imprinting disturbance
Source: Clin Epigenetics. 2021 May 26;13:119. doi: 10.1186/s13148-021-01106-5 (PMC8157728; doi:10.1186/s13148-021-01106-5)
Supplement: Supplementary file 1 — Additional file 1: Table S1. Methylation indices (%) for CpGs determined by pyrosequencing analysis for disease-related iDMRs. [file 13148_2021_1106_MOESM1_ESM.pdf]

Table S1. Methylation indices (%) for CpGs determined by pyrosequencing analysis for disease-related iDMRs.

DMR<br>Genomic position*	CpG	Methylate allele	Character of DMR	Pt. 1	Father	Mother	Pt. 2	Pt. 3	Pt. 4	Pt. 5	Pt. 6	Median<br>(Min ~ Max)†
PLAGL1 **:alt-TSS-DMR**	1	Maternal	Oocyte gDMR	49	49	52	52	40	15	26	52	47 (33 ~ 52)
chr6:144329214-144329359	2			47	46	49	47	40	15	25	49	46 (34 ~ 51)
	3			46	50	50	48	47	18	29	50	48 (40 ~ 56)
	4			33	38	43	39	35	14	23	40	39 (31 ~ 47)
	5			45	45	47	49	46	19	26	53	50 (44 ~ 58)
	6			52	51	46	45	46	16	29	51	49 (41 ~ 55)
	7			57	53	50	48	48	17	31	54	53 (47 ~ 58)
PEG10 **:TSS-DMR**	1	Maternal	Oocyte gDMR	54	57	57	56	59	54	51	14	56 (50 ~ 59)
chr7:94285715-94285869	2			53	58	57	56	58	54	52	15	55 (50 ~ 59)
	3			52	59	55	54	58	52	51	14	53 (48 ~ 59)
	4			52	58	56	55	61	55	54	14	54.5 (50 ~ 59)
	5			48	51	51	50	53	48	49	15	50 (45 ~ 55)
MEST **:alt-TSS-DMR**	1	Maternal	Oocyte gDMR	54	66	61	33	54	56	60	56	60 (56 ~ 70)
chr7:130132206-130132348	2			55	65	61	54	55	56	60	56	59 (55 ~ 69)
	3			49	60	59	33	52	54	56	52	58 (52 ~ 68)
	4			55	67	63	37	60	57	61	54	61 (42 ~ 73)
	5			54	62	59	33	55	56	60	55	57 (47 ~ 66)
	6			55	66	62	37	62	57	60	55	60 (54 ~ 70)
H19/IGF2 **:IG-DMR**	1	Paternal	Sperm gDMR	25	52	51	48	18	55	38	49	51 (48 ~ 54)
chr11:2023224-2023357	2			27	54	53	50	12	56	42	46	51 (45 ~ 57)
	3			27	53	54	50	15	58	38	49	54 (49 ~ 58)
	4			24	47	48	45	13	51	33	43	47 (43 ~ 51)
	5			31	50	50	48	15	55	36	44	50 (46 ~ 54)
	6			19	40	42	39	8	45	32	33	41 (36 ~ 46)
chr11:2019579-2019781	7			20	63	61	58	20	62	43	54	60 (57 ~ 63)
	8			21	59	55	56	17	60	39	53	58 (54 ~60)
	9			20	65	61	58	20	64	43	54	61 (57 ~ 65)
	10			19	60	57	57	20	60	41	50	58 (55 ~ 61)
	11			18	57	55	54	19	57	39	48	55 (52 ~ 57)
	12			19	64	60	61	18	62	43	56	60 (56 ~ 64)
	13			15	46	43	45	13	49	34	38	47 (43 ~ 49)
KCNQ1OT1 **:TSS-DMR**	1	Maternal	Oocyte gDMR	53	52	50	9	54	10	55	15	58 (49 ~ 66)
chr11:2720333-2720487	2			54	53	50	11	56	10	58	19	61 (52 ~ 68)
	3			47	48	47	9	49	9	48	14	48 (41 ~ 54)
	4			48	49	47	9	52	8	50	14	48 (42 ~ 55)
	5			58	57	57	12	60	10	56	18	67 (55 ~ 72)
	6			57	54	57	10	58	10	55	15	64 (55 ~ 71)
MEG3/DLK1 **:IG-DMR (CG4)**	1	Paternal	Sperm gDMR	50	66	64	66	53	59	39	60	58 (49 ~ 68)
chr14:101275613-101275776	2			44	58	57	54	50	62	32	53	54 (40 ~ 62)
	3			78	73	74	72	66	78	54	67	68 (54 ~ 78)
	4			41	56	55	56	50	52	31	51	53.5 (43 ~ 64)
MEG3 **:TSS-DMR (CG7)**	1	Paternal	Secondary DMR	2	50	48	48	48	48	26	48	52 (43 ~ 56)
chr14:101292170-101292336	2			3	51	54	50	51	49	28	48	55 (52 ~ 65)
	3			4	50	50	36	49	48	27	47	37 (32 ~ 55)
	4			7	54	58	49	52	51	30	51	60 (44 ~ 74)
	5			2	46	46	33	42	42	24	44	36 (26 ~ 47)
SNURF **:TSS-DMR**	1	Maternal	Oocyte gDMR	51	52	57	56	52	54	55	46	55 (50 ~ 60)
chr15:25200788-25200897	2			47	49	46	50	47	48	51	41	49 (44 ~ 54)
	3			51	51	53	52	49	49	52	41	51 (46 ~ 57)
	4			51	52	49	52	48	49	51	41	52 (47 ~ 57)
	5			63	61	58	65	64	61	60	55	63 (58 ~ 68)
GNAS A/B **:TSS-DMR**	1	Maternal	Secondary DMR	42	45	42	12	45	42	44	41	41 (34 ~ 45)
chr20:57463531-57463746	2			41	45	41	13	52	41	42	45	40 (33 ~ 45)
	3			43	47	44	14	51	42	45	45	43 (35 ~ 47)
	4			39	41	40	13	45	39	42	41	38 (32 ~ 42)
	5			41	45	42	14	49	41	44	42	40 (33 ~ 45)
	6			38	41	40	13	44	39	39	42	38 (31 ~ 42)
	7			40	43	41	12	51	41	42	45	40 (32 ~ 44)
**Clinical diagnosis**				TS14‡	Normal	Normal	BWS+<br>PHPIb§	SRS	BWS	SRS	BWS	
Reference				1			2					

The methods for pyrosequencing have been described previously,[1] and primers utilized in this study are shown in Table S2.

Abbreviations: DMR, differentially methylated region; gDMR, germline DMR; TS14, Temple syndrome; SRS, Silver-Russell syndrome; PHPIb, pseudohypoparathyroidism type Ib; and BWS, Beckwith-Wiedemann syndrome.

* According to GRCh37/hg19.

† Reference data obtained from 50 control subjects.

‡ TS14-compatible phenotype in infancy to early childhood, *i.e.* , coexistence of typical SRS phenotype satisfying all the six Netchine-Harbison scoring system features and marked hypotonia characteristic of Prader-Willi syndrome at 4 5/12 years of age.

§ Co-existence of clinically recognizable BWS and PHPIb phenotypes.

Methylation indices above and below the reference ranges are highlighted with light orange and light blue, respectively.

References

1. Kagami M, Yanagisawa A, Ota M, Matsuoka K, Nakamura A, Matsubara K, et al. Temple syndrome in a patient with variably methylated CpGs at the primary MEG3/DLK1:IG-DMR and severely hypomethylated CpGs at the secondary MEG3:TSS-DMR. Clin Epigenetics. 2019;11:42.

2. Sano S, Matsubara K, Nagasaki K, Kikuchi T, Nakabayashi K, Hata K, et al. Beckwith-Wiedemann syndrome and pseudohypoparathyroidism type Ib in a patient with multilocus imprinting disturbance: a female-dominant phenomenon? J Hum Genet. 2016;61:765–69.
