# Supplementary material for: ZNF445: a homozygous truncating variant in a patient with Temple syndrome and multilocus imprinting disturbance
Source: Clin Epigenetics. 2021 May 26;13:119. doi: 10.1186/s13148-021-01106-5 (PMC8157728; doi:10.1186/s13148-021-01106-5)
Supplement: Supplementary file 2 — Additional file 2: Table S2. Assessment of consanguinity based on PI_HAT values calculated by PLINK. [file 13148_2021_1106_MOESM2_ESM.pdf]

Table S2. Assessment of consanguinity based on PI_HAT values calculated by PLINK

Family	IID1	IID2	Z0	Z1	Z2	PI_HAT
This family	Patient	Father	0	1	0	0.5
	Patient	Mother	0	1	0	0.5
	Father	Mother	1	0	0	0
Reference*	Child	Father	0	1	0	0.5
	Child	Mother	0	1	0	0.5
	Father	Mother	0.7598	0.2402	0	0.1201

A total of 5,123 common exonic variants have been analyzed by PLINK 1.9 beta (https://www.cog-genomics.org/plink/1.9/).

IID1: Individual ID for first individual; IID2: Individual ID for second individual; Z0: P(IBD=0) (IBD: identity by descent); Z1: P(IBD=1); Z2: P(IBD=2); and PI_HAT: P(IBD=2)+0.5*P(IBD=1) (proportion IBD).

Theoretical PI_HAT values: 1.0 for the same individual or monozygotic twins; 0.5 for first-degree relatives; 0.25 for second-degree relatives, 0.125 for third-degree relatives, and 0 for unrelated individuals.

*For a reference, PI_HAT values have been calculated for a family in which the parents were first cousins (third-degree relatives). The value of 0.1201 is similar to the expected value of 0.125.

Reference

Purcell S, Neale B, Todd-Brown K, Thomas L, Ferreira MAR, Bender D, et al. PLINK: A tool set for whole-genome association and population-based linkage analyses. Am J Hum Genet. 2007;81:559–75.
