# Supplementary material for: ZNF445: a homozygous truncating variant in a patient with Temple syndrome and multilocus imprinting disturbance
Source: Clin Epigenetics. 2021 May 26;13:119. doi: 10.1186/s13148-021-01106-5 (PMC8157728; doi:10.1186/s13148-021-01106-5)
Supplement: Supplementary file 4 — Additional file 4: Table S4. The list of abnormally methylated 24 probes (CpGs) on non-iDMRs. [file 13148_2021_1106_MOESM4_ESM.pdf]

Table S4. The list of abnormally methylated 24 probes (CpGs) on non-iDMRs.

Chr.	Genomic position	Target ID	Mean β values in controls	SD	Mean + 3 SD	Mean - 3 SD	β values Patient	Delta-β Patient	CpG island*	Relative position to CpG island†	Gene near the CpG island
1	1914597	cg18270426	0.93260524	0.01787403	0.98622734	0.87898314	0.8781859	-0.05441934	chr1:1910268-1913800	South Shore	*KIAA1751*
1	20696747	cg11417178	0.90511118	0.0265362	0.98471979	0.82550256	0.818985	-0.08612618	chr1:20692582-20693534	South Shelf	
2	118616155	cg25619440	0.91010796	0.02473747	0.98432037	0.83589554	0.8104967	-0.09961126	chr2:118616575-118618163	North Shore	
2	218843411	cg13711385	0.91341694	0.0288209	0.99987963	0.82695425	0.8235939	-0.08982304	chr2:218843460-218843742	North Shore	
5	172662741	cg03625415	0.21249239	0.0563137	0.38143348	0.04355129	0.527005	0.31451261	chr5:172663616-172664584	North Shore	*NKX2-5*
6	170478393	cg06391732	0.84738729	0.03004668	0.93752733	0.75724725	0.7536973	-0.09368999	chr6:170474406-170475553	South Shelf	
6	170478454	cg19680631	0.90813458	0.01723893	0.95985138	0.85641777	0.8412939	-0.06684067	chr6:170474406-170475553	South Shelf	
7	2592339	cg08092333	0.93495116	0.01974107	0.99417438	0.87572794	0.8457728	-0.08917836	chr7:2594724-2595538	North Shelf	*C7orf27*
8	1054899	cg23984176	0.83232428	0.04746852	0.97472985	0.68991872	0.6842682	-0.14805608	chr8:1053354-1053804	South Shore	
10	124133822	cg10576280	0.12479224	0.03707881	0.23602866	0.01355581	0.3074526	0.18266036	chr10:124134088-124134933	North Shore	*PLEKHA1*
10	134943309	cg25142028	0.7964697	0.03827707	0.9113009	0.6816385	0.6733098	-0.1231599	chr10:134944511-134944792	North Shore	*GPR123*
11	430036	cg16362232	0.90942373	0.02521472	0.98506788	0.83377957	0.8177336	-0.09169013	chr11:430814-431775	North Shore	*ANO9*
11	1108632	cg04531584	0.71429302	0.05086846	0.86689838	0.56168765	0.556056	-0.15823702	chr11:1107802-1108041	South Shore	
11	61583871	cg20295071	0.05528314	0.01290366	0.09399411	0.01657218	0.00120796	-0.05407518	chr11:61582573-61584728	CpG island	*MIR1908;FADS2;FADS1*
13	112630399	cg09717927	0.87752055	0.0244472	0.95086214	0.80417897	0.7966517	-0.08086885	chr13:112630568-112630796	North Shore	
14	102101660	cg19571540	0.89517326	0.03055704	0.98684437	0.80350215	0.7598878	-0.13528546	chr14:102101165-102101596	South Shore	
14	105120586	cg10793244	0.95137386	0.01179816	0.98676835	0.91597938	0.8496499	-0.10172396	chr14:105119773-105120005	South Shore	
15	41524250	cg08173216	0.29851026	0.08198841	0.54447547	0.05254504	0.00378449	-0.29472577	chr15:41522828-41523732	South Shore	*CHP;EXD1*
16	88496476	cg07745707	0.85897398	0.04448464	0.99242789	0.72552007	0.7221273	-0.13684668	chr16:88496201-88497763	CpG island	*ZNF469*
17	19771783	cg04036329	0.94343477	0.01505712	0.98860615	0.8982634	0.8931007	-0.05033407	chr17:19771609-19771814	CpG island	*ULK2*
18	76766007	cg16152072	0.8992147	0.02586565	0.97681164	0.82161776	0.8197969	-0.0794178	chr18:76765747-76766833	CpG island	
20	60527061	cg12751644	0.75751243	0.07996424	0.99740515	0.51761972	0.4896641	-0.26784833	chr20:60518033-60523876	South Shelf	
22	36959492	cg21569107	0.92162856	0.01808392	0.97588032	0.86737679	0.8668609	-0.05476766	chr22:36960369-36961054	North Shore	*CACNG2*
22	50705546	cg09778961	0.83521201	0.04854833	0.980857	0.68956702	0.6635463	-0.17166571	chr22:50705406-50706553	CpG island	*MAPK11*

The 24 probes are associated with |β| of > 3 SD of the mean and |Δβ| of > 0.05.
Beta values < mean - 3 SD and > mean + 3 SD are written in green and red numbers, respectively.
Delta beta values < 0.05 and > 0.05 are highlighted with green and red backgroundeds, respectively.
The probes with age-related drift and sex-bias have been excluded.
* CpG island nearest to the probe (accoridng to the UCSC genome browser).
† For the definition of north and south shores and shelves, see Figure S2.
