# Supplementary material for: ZNF445: a homozygous truncating variant in a patient with Temple syndrome and multilocus imprinting disturbance
Source: Clin Epigenetics. 2021 May 26;13:119. doi: 10.1186/s13148-021-01106-5 (PMC8157728; doi:10.1186/s13148-021-01106-5)
Supplement: Supplementary file 5 — Additional file 5: Table S5 Primers utilized in the present study. [file 13148_2021_1106_MOESM5_ESM.pdf]

Table S5. Primers utilized in the present study.

	Forward primer	Reverse primer	Sequence primer
<Pyrosequencing>			
PLAGL1:alt-TSS-DMR	GGGGTAGTYGTGTTTATAGTTTAG	biotin-CCCAAACACCTACCCTAC	GGGTAGTYGTGTTTATAGTTTAGT
MEST:alt-TSS-DMR	GTGTGGTTGGYGGTTTTGGGATTA	biotin-ACACCCCCTCCTCAAATA	TGTTTTTGGGYGAAAATTTTAT
PEG10:TSS-DM*R*	AGAAATTTGATTGYGTTTTGAGGAGAATA	biotin-ACAAAAAAAATAAAATCCCACACCTTAA	AGTTTGGAGAAAGGTT
H19/IGF2:IG-DMR-1	TTTGGGAGAGTTTGTGAGG	biotin-CCCCAAACCRATTCCCATCCAATTA	GRTAATATGYGGTTTTTAGATAGG
H19/IGF2:IG-DMR-2	GGGTTTTYGGAGGTTTTTTGGGAATA	biotin-ACTTAAATCCCAAACCATAACAC	GGAATAGGAYGTTTATGGGAG
KCNQ1OT1:TSS-DM*R*	GGATTTAGAATTAYGATGYGGATTTTA	biotin-TCCCATCTACACCTTATAAACA	TTTTGAATTATTATGAGAATTATAG
MEG3/DLK1:IG-DMR	ATTTGGTATTTGTAGTTTTATGTTAAGATG	biotin-AATCAAAACAACTCAAATCCTTTATAAC	AATTGGGTTTGTTAGTAG
MEG3:TSS-DMR	TTGTGTTTGAATTTATTTTGTTT	biotin-CCCCAAATTCTATAACAAATTACTCT	GTGTTTGAATTTATTTTGTTT
SNURF:TSS-DMR	GTTATGGTAGTGGATTAGGGGGATGA	biotin-CCTTCCCCCTACCTCCCA	ATAGTGGTGGGGGTT
GNAS A/B:TSS-DMR	GGGATATTTGAGATTTTGAAAGAA	biotin-AATACAAAACCTCCCCTACT	GTTATTTTTTTTATTTGGGAGGA
<Sanger sequencing >			
ZNF445 variant	TGTCAGTGGTGTGGGAAAGA	GAGGGGCATCTCTTCACTTG	
<Quantitative real-time PCR>			
q-PCR-1	AAGAGTGGGCTTCTCACTGG	GCTGCACTATCCCATTAGGC	
q-PCR-2	CTACCGTCTGCACCGAGAA	GCTTCTCCTGGGTGTGAATC	
q-PCR-3	TGGGAGACATACCCTTTCCA	CTCTGGCTGCAAGCTTCTTT	
<Mutagenesis>			
ZNF445- c.2803C>T	TTCCTCTTAGGACACAAAGTTGAGA	GTGTCCTAAGAGGAAGACCGTGCAG	
<Bisulfite sequencing>			
MEG3-DLK1:IG-DMR	GTTTATTGGGTTGGGTTTTGTTAG	ACCAATTACAATACCACAAAATTAC	
