# Supplementary material for: ZNF445: a homozygous truncating variant in a patient with Temple syndrome and multilocus imprinting disturbance
Source: Clin Epigenetics. 2021 May 26;13:119. doi: 10.1186/s13148-021-01106-5 (PMC8157728; doi:10.1186/s13148-021-01106-5)
Supplement: Supplementary file 6 — Additional file 6: Figure S1. Exclusion of a heterozygous deletion involving ZNF445 in patient 1. [file 13148_2021_1106_MOESM6_ESM.pdf]

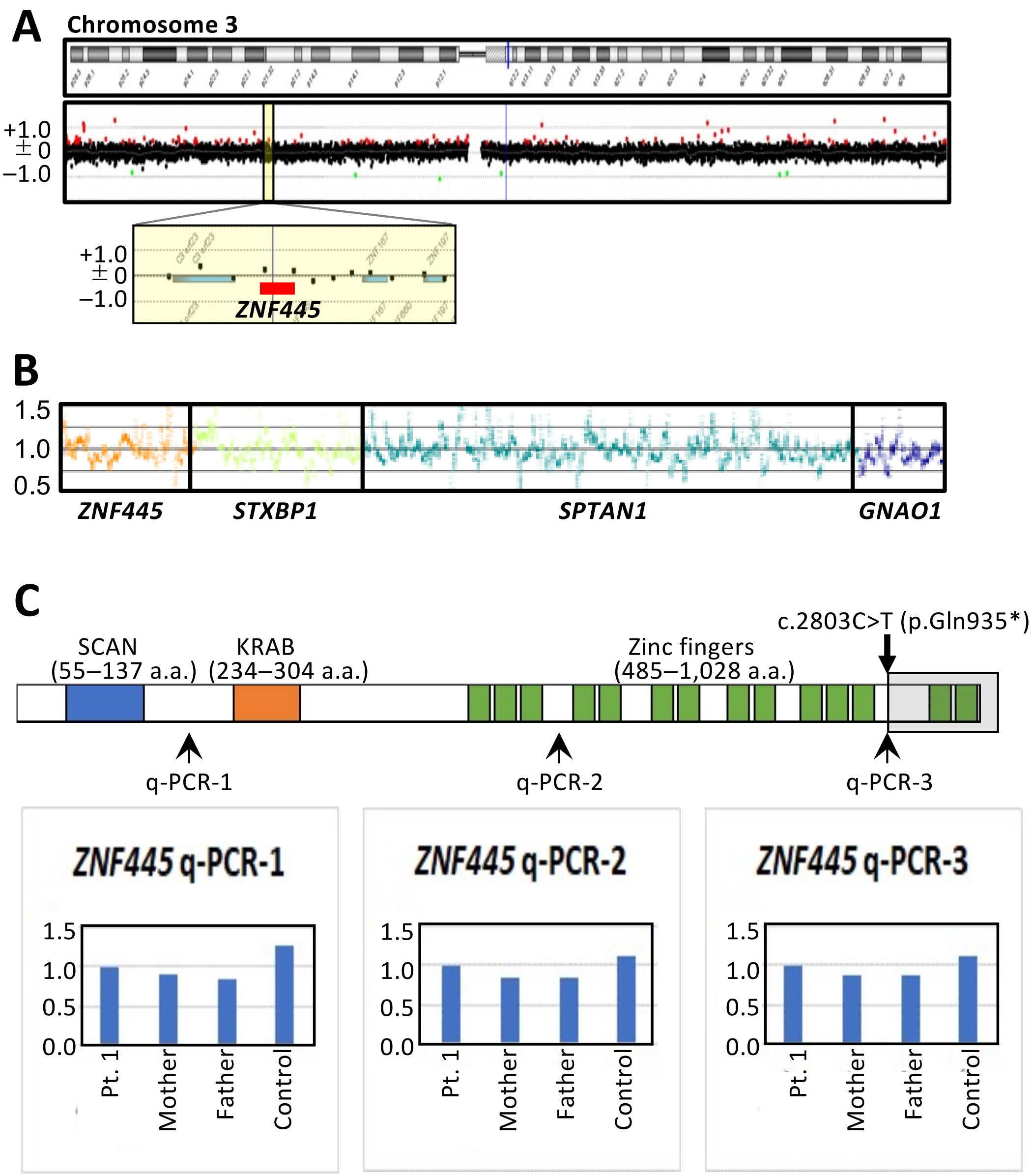

Figure S1. Exclusion of a heterozygous deletion involving *ZNF445* in patient 1.

A. Genomewide array comparative genomic hybridization analysis.

B. Exome sequencing-based CNV calling developed by Nord et al.[1]

C. Quantitative real-time PCR analysis for three positions on *ZNF445*.

Reference

1. Nord AS, Lee M, King MC, Walsh T. Accurate and exact CNV identification from targeted high-throughput sequence data. BMC Genomics. 2011;12:184.
