# Supplementary material for: ZNF445: a homozygous truncating variant in a patient with Temple syndrome and multilocus imprinting disturbance
Source: Clin Epigenetics. 2021 May 26;13:119. doi: 10.1186/s13148-021-01106-5 (PMC8157728; doi:10.1186/s13148-021-01106-5)
Supplement: Supplementary file 7 — Additional file 7: Figure S2. Violin plots of the β-values for autosomal probes in patient 1, the parents, and 24 control subjects (average). [file 13148_2021_1106_MOESM7_ESM.pdf]

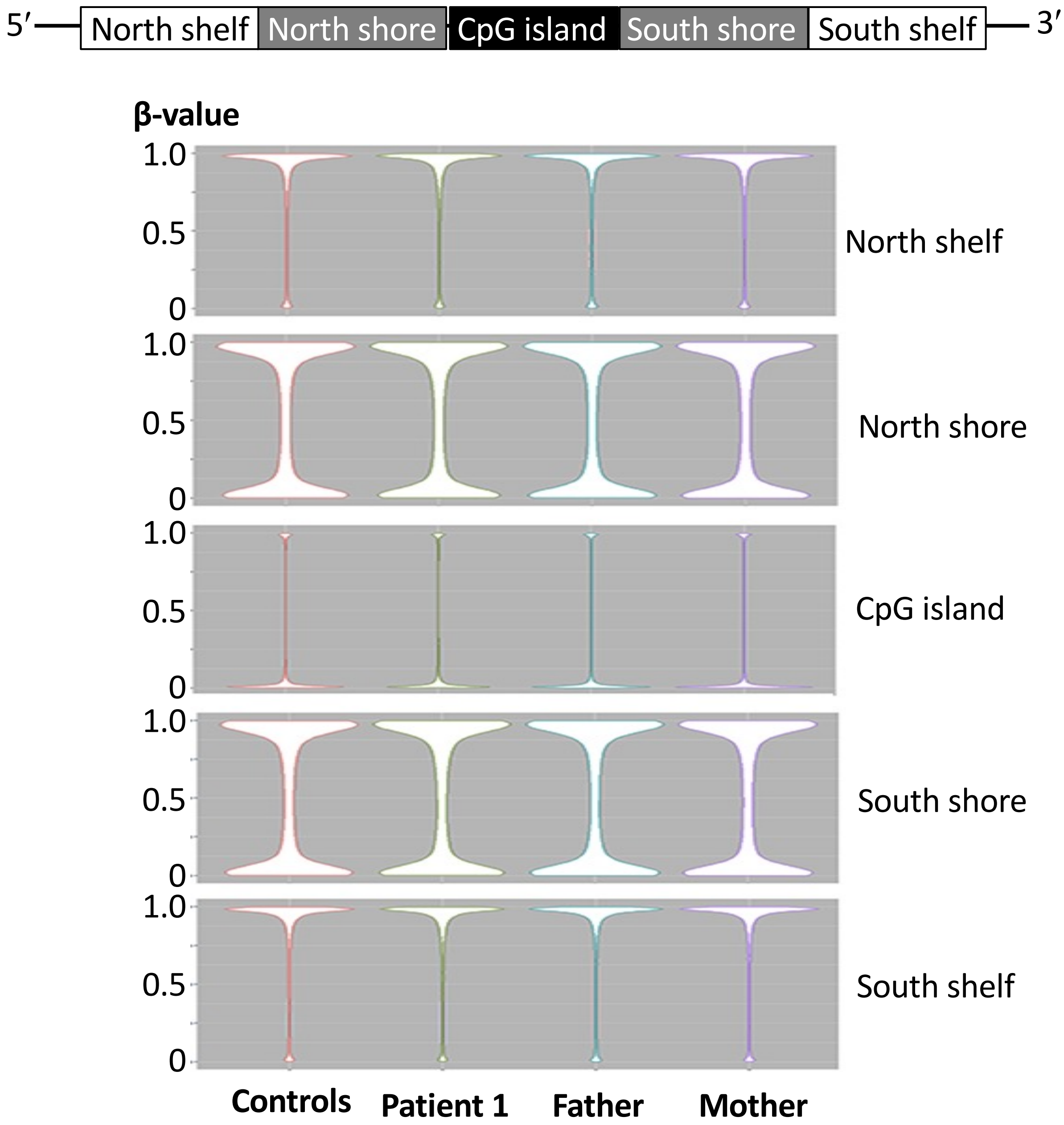

Figure S2. Violin plots of the β-values for autosomal probes in patient 1, the parents, and 24 control subjects (average). CpG islands are defined as regions that span > 200 bp in length and contain > 50% of CpGs based on the UCSC criteria. Shore and Shelf denote the regions up to 2 kb and 2–4 kb from CpG islands, respectively. North and south mean 5′ and 3′ sides of CpG islands, respectively. 143630 probes reside on CpG islands, 74216 probes on North Shore, 63446 probes on South Shore, 27280 probes on North Shelf, and 25296 probes on South Shelf.
