# Supplementary material for: ZNF445: a homozygous truncating variant in a patient with Temple syndrome and multilocus imprinting disturbance
Source: Clin Epigenetics. 2021 May 26;13:119. doi: 10.1186/s13148-021-01106-5 (PMC8157728; doi:10.1186/s13148-021-01106-5)
Supplement: Supplementary file 3 — Additional file 3: Table S3. Methylation values of 855 probes (CpGs) on differentially methylated regions, measured by Infinium MethylationEPIC Kit. [file 13148_2021_1106_MOESM3_ESM.pdf]

Table S3. Methylation values of 855 probes (CpGs) on differentially methylated regions, measured by Infinium MethylationEPIC Kit.

Chr.	Genomic position	Target ID	Name of DMRs	450K probes	Mean β values in controls	SD	Mean - 3 SD	Mean + 3 SD	β values Patient	Delta-β Patient	β values Father	Delta-β Father	β values Mother	Delta-β Mother
1	36915841	cg27439456	OSCP1	Yes	0.193439981	0.065998658	-0.004555993	0.391435955	0.162212301	-0.022298253	0.164328363	-0.02018219	0.229963009	0.045452455
1	36915865	cg08878450	OSCP1	Yes	0.417937518	0.08264052	0.170015959	0.665859077	0.388105273	-0.010819845	0.337544032	-0.061381085	0.552949607	0.15402449
1	36916006	cg14912727	OSCP1	Yes	0.041453072	0.007097445	0.020160737	0.062745407	0.037234459	-0.00311173	0.034926573	-0.005419616	0.044190478	0.003844289
1	36916013	cg16583330	OSCP1	Yes	0.015857457	0.004377236	0.002725748	0.028989166	0.015089673	-0.000296723	0.015672971	0.000286575	0.008937022	-0.006449374
1	36916040	cg10035922	OSCP1	Yes	0.00610117	0.001952576	0.000243442	0.011958898	0.003893416	-0.001902997	0.005514626	-0.000281787	0.003900735	-0.001895678
1	36916160	cg00578252	OSCP1	Yes	0.013048481	0.00293805	0.004234332	0.02186263	0.011969365	-0.000439884	0.010863368	-0.001545881	0.010691374	-0.001717874
1	36916165	cg11798976	OSCP1	Yes	0.002685999	0.00113309	-0.000713272	0.006085269	0.002840409	0.000242155	0.001335501	-0.001262753	0.002669906	7.16515E-05
1	36916179	cg15725228	OSCP1	Yes	0.013622069	0.00280758	0.00519933	0.022044808	0.010229475	-0.003037942	0.010715863	-0.002551555	0.013107167	-0.00016025
1	36916191	cg09662887	OSCP1	Yes	0.01370958	0.006680768	-0.006332724	0.033751884	0.015185413	0.002749353	0.011033188	-0.001402872	0.008867687	-0.003568373
1	36916194	cg06367472	OSCP1	Yes	0.006344305	0.001468057	0.001940135	0.010748476	0.005109455	-0.001013667	0.006704272	0.00058115	0.004696409	-0.001426713
1	36916196	cg12630409	OSCP1	Yes	0.020766104	0.003705575	0.009649378	0.03188283	0.018813055	-0.001126901	0.023033955	0.003093999	0.019182835	-0.000757122
1	36916502	cg22397854	OSCP1	Yes	0.008641881	0.002616505	0.000792368	0.016491395	0.008228236	-0.000197942	0.006811316	-0.001614861	0.007460897	-0.000965281
1	36916606	cg03954991	OSCP1	Yes	0.097806653	0.035820005	-0.009653363	0.205266669	0.061239378	-0.033693625	0.06101903	-0.033913972	0.162365588	0.067432586
1	40024972	cg10243676	**PPIEL:Ex1-DMR**	Yes	0.664496286	0.049027564	0.517413595	0.811578977	0.629505458	-0.003632491	0.684304935	0.051166986	0.723651498	0.09051355
1	40025233	cg11704876	**PPIEL:Ex1-DMR**	Yes	0.736911929	0.052622943	0.579043099	0.894780758	0.737741803	0.033708557	0.728285211	0.024251965	0.784397523	0.080364277
1	40025412	cg22862450	**PPIEL:Ex1-DMR**	Yes	0.830563697	0.031083953	0.737311838	0.923815557	0.815661066	0.022112524	0.807391815	0.013843273	0.868020801	0.074472258
1	40025416	cg15057250	**PPIEL:Ex1-DMR**	Yes	0.849652267	0.035854201	0.742089663	0.957214871	0.817011119	0.005256765	0.867310468	0.055556114	0.878659986	0.066905632
1	55271674	cg21872908	C1orf177	Yes	0.97651074	0.008548325	0.950865766	1.002155715	0.971560705	0.036170561	0.974205562	0.038815419	0.987883307	0.052493164
1	68512540	cg03641225	**DIRAS3:Ex2-DMR**	Yes	0.32374034	0.039248731	0.205994147	0.441486534	0.272433587	-0.034170818	0.298730007	-0.007874398	0.447876679	0.141272274
1	68512808	cg20149168	**DIRAS3:Ex2-DMR**	Yes	0.675861738	0.0575393	0.503243839	0.848479638	0.611448525	-0.030030994	0.679895252	0.038415733	0.822782937	0.181303418
1	68512929	cg00448707	**DIRAS3:Ex2-DMR**	Yes	0.747525614	0.047213546	0.605884975	0.889166253	0.706765387	-0.005464637	0.727451287	0.015221263	0.874149028	0.161919005
1	68513064	cg21808053	**DIRAS3:Ex2-DMR**	Yes	0.475449637	0.052537588	0.317836874	0.633062399	0.44447504	-0.006646688	0.417526957	-0.033594771	0.604032148	0.15291042
1	68515789	cg02317907	**DIRAS3:TSS-DMR**	Yes	0.794813455	0.029373037	0.706694345	0.882932564	0.790869355	0.031189742	0.793642929	0.033963316	0.792244249	0.032564636
1	68515873	cg17943391	**DIRAS3:TSS-DMR**	Yes	0.460785155	0.047355258	0.318719381	0.60285093	0.435355243	-0.001728827	0.440251075	0.003167005	0.485355939	0.048271868
1	68515978	cg19114595	**DIRAS3:TSS-DMR**	Yes	0.767041149	0.034165681	0.664544106	0.869538192	0.741980559	0.009977039	0.766013411	0.034009891	0.751021283	0.019017763
1	68516081	cg12070746	**DIRAS3:TSS-DMR**	Yes	0.515116616	0.042619476	0.387258189	0.642975044	0.455925484	-0.034663112	0.524804841	0.034216245	0.538644861	0.048056265
1	68516102	cg16314899	**DIRAS3:TSS-DMR**	Yes	0.704511094	0.048367328	0.55940911	0.849613077	0.648249071	-0.022980238	0.761115484	0.089886175	0.695057218	0.023827909
1	68516139	cg25755905	**DIRAS3:TSS-DMR**	Yes	0.511188161	0.045830092	0.373697885	0.648678438	0.456662391	-0.028777356	0.489953587	0.00451384	0.559105597	0.073665849
1	68516273	cg22500004	**DIRAS3:TSS-DMR**	Yes	0.713508053	0.042016139	0.587459635	0.839556471	0.65435697	-0.027410304	0.755540238	0.073772964	0.707109346	0.025342072
1	68516280	cg05949203	**DIRAS3:TSS-DMR**	Yes	0.660755468	0.03248297	0.563306559	0.758204377	0.595062925	-0.036289307	0.676495197	0.045142965	0.688593242	0.057241011
1	68516375	cg19694923	**DIRAS3:TSS-DMR**	NA	0.563736671	0.03399298	0.461757732	0.665715611	0.537239273	-0.001700863	0.585825063	0.046884927	0.560685393	0.021745256
1	68516454	cg05392265	**DIRAS3:TSS-DMR**	NA	0.616051106	0.042130167	0.489660605	0.742441607	0.559269476	-0.028634124	0.64930083	0.06139723	0.62692473	0.03902113
1	68516464	cg06191076	**DIRAS3:TSS-DMR**	Yes	0.608674398	0.041047154	0.485532935	0.73181586	0.583110527	0.003205634	0.5944587	0.014553807	0.645615586	0.065710692
1	68516519	cg13208159	**DIRAS3:TSS-DMR**	Yes	0.539499662	0.050705931	0.387381869	0.691617455	0.497939064	-0.016201545	0.572301854	0.058161245	0.546212352	0.032071743
1	68516714	cg13099417	**DIRAS3:TSS-DMR**	Yes	0.702774361	0.046685553	0.562717702	0.84283102	0.704153332	0.032335088	0.668661615	-0.00315663	0.769346696	0.097528451
1	68517178	cg27545611	**DIRAS3:TSS-DMR**	Yes	0.686387453	0.044120804	0.554025042	0.818749864	0.523210643	-0.131356512	0.73438218	0.079815025	0.724060011	0.069492856
1	68517206	cg16148134	**DIRAS3:TSS-DMR**	Yes	0.526493991	0.062517914	0.33894025	0.714047733	0.341076999	-0.162694364	0.575833119	0.072061757	0.563853344	0.060081981
1	68517256	cg13605615	**DIRAS3:TSS-DMR**	Yes	0.477543356	0.058517368	0.301991254	0.653095459	0.334376755	-0.119480449	0.500565949	0.046708745	0.528300118	0.074442914
1	68517274	cg10038185	**DIRAS3:TSS-DMR**	Yes	0.547265804	0.064979927	0.352326024	0.742205584	0.343336985	-0.179544488	0.614669738	0.091788264	0.59633284	0.073451367
1	68517437	cg14230016	**DIRAS3:TSS-DMR**	NA	0.630985563	0.045184254	0.4954328	0.766538326	0.519446412	-0.084274676	0.648145177	0.044424089	0.663736951	0.060015863
1	68517455	cg01547550	**DIRAS3:TSS-DMR**	NA	0.591609114	0.039653343	0.472649084	0.710569144	0.510439191	-0.055434652	0.591201732	0.025327888	0.62767235	0.061798506
1	68517463	cg14059179	**DIRAS3:TSS-DMR**	NA	0.517425305	0.040261123	0.396641935	0.638208675	0.42329104	-0.071463919	0.551690388	0.056935429	0.534251804	0.039496845
1	92414521	cg03421440	BRDT	Yes	0.927406767	0.029558715	0.838730624	1.016082911	0.929087651	0.04157273	0.901127777	0.013612856	0.938852457	0.051337536
1	92414723	cg14732540	BRDT	Yes	0.683304968	0.083932346	0.431507931	0.935102005	0.607920099	-0.042096938	0.609746617	-0.040270419	0.775954168	0.125937132
1	95698955	cg24808901	LOC101928118	Yes	0.818137791	0.059113219	0.640798135	0.995477447	0.791194518	0.008008625	0.747983301	-0.035202592	0.85367969	0.070493797
1	95699038	cg26537280	LOC101928118	Yes	0.488411373	0.088266019	0.223613316	0.75320943	0.495168286	0.030148472	0.394625084	-0.07039473	0.503556084	0.03853627
1	95699098	cg03123541	LOC101928118	Yes	0.65618013	0.101019184	0.353122579	0.959237682	0.636517385	0.008857785	0.551360366	-0.076299234	0.734964268	0.107304668
1	95699314	cg06440946	LOC101928118	Yes	0.060378632	0.028983669	-0.026572375	0.14732964	0.062139164	0.005072096	0.033508158	-0.02355891	0.072415129	0.015348062
1	158900385	cg11177526	PYHIN1	Yes	0.736840866	0.106682362	0.416793781	1.056887951	0.821913094	0.122711553	0.642411322	-0.056790219	0.752771095	0.053569554
1	228503802	cg18857768	OBSCN	Yes	0.984340369	0.003803447	0.972930029	0.99575071	0.986857643	0.043739128	0.981283393	0.038164877	0.985770101	0.042651586
1	240656343	cg01809217	MIR1273E	Yes	0.431955125	0.075528968	0.205368222	0.658542028	0.389212517	-0.018039457	0.486914006	0.079662032	0.410093214	0.00284124
1	240656514	cg02577267	MIR1273E	Yes	0.686965947	0.083905389	0.435249778	0.938682115	0.635618528	-0.018105081	0.767081685	0.113358076	0.636268259	-0.017455349
1	240656587	cg22879626	MIR1273E	Yes	0.493501203	0.082526106	0.245922886	0.741079521	0.481151707	0.016505476	0.542094913	0.077448681	0.414721202	-0.04992503
1	240656668	cg04391463	MIR1273E	Yes	0.473289199	0.080391867	0.232113597	0.7144648	0.417173525	-0.031251158	0.537227598	0.088802915	0.43738264	-0.011042043
1	240656738	cg00081729	MIR1273E	Yes	0.816222941	0.066503089	0.616713674	1.015732207	0.822496319	0.04441006	0.814723231	0.036636972	0.810111335	0.032025076
2	130345045	cg05962382	LOC151121	Yes	0.865737914	0.076523351	0.636167861	1.095307966	0.951496009	0.123921981	0.782913194	-0.044660834	0.893794207	0.066220179
2	130345206	cg05903289	LOC151121	Yes	0.698609526	0.133009129	0.29958214	1.097636913	0.807360425	0.139156732	0.595432642	-0.072771051	0.809010052	0.140806359
2	130345356	cg19182557	LOC151121	Yes	0.806496331	0.073830914	0.585003591	1.027989072	0.840822175	0.070043223	0.774420195	0.003641243	0.830350224	0.059571272
2	172974139	cg11557618	DLX2_AS1	Yes	0.203964428	0.066795932	0.003576631	0.404352225	0.202163357	0.007994905	0.236695543	0.042527091	0.169212454	-0.024955998
2	207068255	cg19696983	**GPR1-AS:TSS-DMR**	Yes	0.98936564	0.003124939	0.979990822	0.998740458	0.985176005	0.037201055	0.989396833	0.041421883	0.992321394	0.044346444
2	207068318	cg23239484	**GPR1-AS:TSS-DMR**	Yes	0.979406779	0.004496776	0.96591645	0.992897108	0.976847238	0.038475695	0.986406079	0.048034536	0.976197569	0.037826026
2	207068410	cg13443116	**GPR1-AS:TSS-DMR**	Yes	0.993995682	0.001683702	0.988944575	0.999046788	0.993313137	0.040751894	0.994093785	0.041532543	0.994155161	0.041593919
2	207114925	cg22719723	**ZDBF2/GPR1:IG-DMR**	NA	0.667309887	0.050813717	0.514868736	0.819751038	0.670229928	0.034905007	0.722441547	0.087116626	0.66032314	0.02499822
2	207116071	cg25535070	**ZDBF2/GPR1:IG-DMR**	Yes	0.831189404	0.02882109	0.744726133	0.917652675	0.82199562	0.027774019	0.833821893	0.039600293	0.8368299	0.0426083
2	207116402	cg04359324	**ZDBF2/GPR1:IG-DMR**	Yes	0.721082268	0.037275218	0.609256615	0.832907922	0.748228275	0.059820556	0.717862985	0.029455265	0.718064899	0.02965718
2	207118254	cg20471298	**ZDBF2/GPR1:IG-DMR**	Yes	0.716235283	0.030972739	0.623317067	0.8091535	0.73785022	0.054569172	0.688721499	0.005440452	0.72173133	0.038450283
2	207118289	cg03428109	**ZDBF2/GPR1:IG-DMR**	Yes	0.856852856	0.02081175	0.794417607	0.919288105	0.858422963	0.038003693	0.846829024	0.026409754	0.885697913	0.065278643
2	207125020	cg15093040	**ZDBF2/GPR1:IG-DMR**	NA	0.614685864	0.050236457	0.463976493	0.765395234	0.620401639	0.03378598	0.5661021	-0.02051356	0.656963369	0.070347709
2	207125061	cg17133012	**ZDBF2/GPR1:IG-DMR**	NA	0.467647629	0.053383244	0.307497897	0.627797361	0.384944601	-0.059217033	0.478893544	0.034731909	0.504973185	0.06081155
2	207125269	cg16353127	**ZDBF2/GPR1:IG-DMR**	NA	0.01794966	0.004842191	0.003423089	0.032476232	0.014306126	-0.003045342	0.014862945	-0.002488522	0.01980116	0.002449692
2	207125466	cg04918871	**ZDBF2/GPR1:IG-DMR**	NA	0.647682423	0.058145971	0.47324451	0.822120336	0.66937105	0.052328492	0.646574963	0.029532405	0.620632328	0.00358977
2	207125607	cg03141680	**ZDBF2/GPR1:IG-DMR**	NA	0.712014352	0.048882343	0.565367322	0.858661382	0.694234427	0.015489478	0.713421739	0.03467679	0.743538325	0.064793376
2	207127227	cg25307915	**ZDBF2/GPR1:IG-DMR**	Yes	0.694445696	0.031376524	0.600316123	0.788575269	0.730380411	0.067991338	0.711322621	0.048933547	0.651964096	-0.010424977
2	207127365	cg19366042	**ZDBF2/GPR1:IG-DMR**	Yes	0.722365071	0.037796208	0.608976448	0.835753693	0.722681252	0.03313391	0.696571041	0.0070237	0.771659319	0.082111978
2	207136185	cg10583266	**ZDBF2/GPR1:IG-DMR**	Yes	0.496482002	0.059408434	0.318256699	0.674707304	0.508420456	0.03798167	0.476903059	0.006464274	0.518697639	0.048258853
4	6107132	cg16724588	JAKMIP1	Yes	0.512801282	0.03931226	0.394864503	0.630738062	0.489724113	-0.002086895	0.567784173	0.075973164	0.464724392	-0.027086616
4	6107281	cg23166781	JAKMIP1	Yes	0.530358289	0.039421426	0.412094012	0.648622566	0.444931884	-0.064465853	0.590446695	0.081048958	0.487379706	-0.022018031
4	6107321	cg06231140	JAKMIP1	Yes	0.472957499	0.048006614	0.328937658	0.616977341	0.453929998	0.000915004	0.538236775	0.085221781	0.410627583	-0.042387411
4	6107340	cg22098660	JAKMIP1	Yes	0.592492229	0.04681583	0.452044739	0.73293972	0.546046814	-0.021019613	0.640460143	0.073393716	0.530326312	-0.036740114
4	6107634	cg21003654	JAKMIP1	Yes	0.72130429	0.051006866	0.568283692	0.874324888	0.695657353	0.007073638	0.666273135	-0.022310579	0.729084424	0.04050071
4	89618325	cg18772071	**NAP1L5:TSS-DMR**	Yes	0.560053439	0.037345421	0.448017177	0.672089701	0.526000979	-0.009613345	0.588107159	0.052492836	0.578221637	0.042607314
4	89618534	cg05065100	**NAP1L5:TSS-DMR**	Yes	0.610683479	0.036226606	0.50200366	0.719363299	0.603794532	0.019658771	0.612684524	0.028548763	0.611500985	0.027365224
4	89618638	cg12759554	**NAP1L5:TSS-DMR**	Yes	0.604678713	0.043026656	0.475598745	0.733758681	0.552875547	-0.024326192	0.628216697	0.051014958	0.604534943	0.027333205
4	89618668	cg01578057	**NAP1L5:TSS-DMR**	Yes	0.515428774	0.038760722	0.399146609	0.631710939	0.506508245	0.015301962	0.503094484	0.011888201	0.524012151	0.032805868
4	89618862	cg27150681	**NAP1L5:TSS-DMR**	Yes	0.683828539	0.020305776	0.62291121	0.744745867	0.675892799	0.021158495	0.668613652	0.013879347	0.695091928	0.040357624
4	89618983	cg23954636	**NAP1L5:TSS-DMR**	Yes	0.547815652	0.024158045	0.475341515	0.620289788	0.534816506	0.00968184	0.565562495	0.040427829	0.54419811	0.019063444
4	89619015	cg01174175	**NAP1L5:TSS-DMR**	Yes	0.67832085	0.030764867	0.58602625	0.770615451	0.684644527	0.037741718	0.687498266	0.040595457	0.663927247	0.017024437
4	89619024	cg06617468	**NAP1L5:TSS-DMR**	Yes	0.678719529	0.018840891	0.622196857	0.7352422	0.648074501	-0.002389335	0.711272044	0.060808207	0.671050511	0.020586674
4	89619031	cg18607468	**NAP1L5:TSS-DMR**	Yes	0.602097026	0.025458856	0.525720459	0.678473593	0.58329776	0.006641705	0.582036772	0.005380717	0.613958279	0.037302224
4	89619039	cg11300971	**NAP1L5:TSS-DMR**	Yes	0.734610171	0.028389199	0.649442573	0.819777769	0.713711837	0.011888495	0.780588523	0.078765182	0.73790923	0.036085888
4	89619086	cg13610072	**NAP1L5:TSS-DMR**	Yes	0.730342283	0.033798033	0.628948185	0.831736381	0.71905302	0.021010056	0.781472063	0.083429099	0.701523954	0.00348099
4	89619237	cg07539802	**NAP1L5:TSS-DMR**	Yes	0.820275335	0.029765378	0.7309792	0.90957147	0.821605596	0.03766832	0.852304382	0.068367105	0.788233023	0.004295746
5	135414859	cg11852404	**VTRNA2-1:DMR**	Yes	0.81244905	0.09259313	0.534669659	1.090228441	0.820024281	0.045029623	0.903409742	0.128415084	0.675594846	-0.099399812
5	135415130	cg16684184	**VTRNA2-1:DMR**	Yes	0.681000315	0.088953723	0.414139147	0.947861484	0.623946297	-0.023110057	0.777931724	0.130875369	0.631414232	-0.015642123
5	135415191	cg00308130	**VTRNA2-1:DMR**	Yes	0.41349194	0.179874101	-0.126130362	0.953114241	0.351409467	-0.03942638	0.589394239	0.198558392	0.151718295	-0.239117552
5	135415259	cg15837280	**VTRNA2-1:DMR**	Yes	0.256951972	0.150334572	-0.194051744	0.707955688	0.15085798	-0.09057592	0.432576935	0.191143035	0.059694559	-0.181739341
5	135415694	cg07158503	**VTRNA2-1:DMR**	Yes	0.562927406	0.258951787	-0.213927956	1.339782768	0.752629054	0.224765327	0.734546413	0.206682686	0.112362649	-0.415501078
5	135415763	cg04515200	**VTRNA2-1:DMR**	Yes	0.264945095	0.147109327	-0.176382886	0.706273075	0.343239179	0.098326005	0.369244383	0.124331209	0.047618222	-0.197294952
5	135415782	cg13581155	**VTRNA2-1:DMR**	Yes	0.15988386	0.104946037	-0.15495425	0.47472197	0.222931279	0.076224532	0.224434043	0.077727297	0.010109984	-0.136596763
5	135415820	cg11978884	**VTRNA2-1:DMR**	Yes	0.243031646	0.140337194	-0.177979936	0.664043229	0.322419007	0.097293356	0.341961458	0.116835806	0.035367405	-0.189758247
5	135415949	cg11608150	**VTRNA2-1:DMR**	Yes	0.407798811	0.220835067	-0.254706392	1.070304013	0.588192016	0.208421823	0.589235595	0.209465403	0.023819848	-0.355950344
5	135416030	cg06478886	**VTRNA2-1:DMR**	Yes	0.458379599	0.210458239	-0.172995119	1.089754317	0.580752164	0.152867586	0.607094984	0.179210406	0.102116606	-0.325767972
5	135416206	cg04481923	**VTRNA2-1:DMR**	Yes	0.492004449	0.274467581	-0.331398293	1.315407191	0.670772344	0.213713122	0.72828659	0.271227368	0.020224823	-0.436834399
5	135416332	cg18678645	**VTRNA2-1:DMR**	Yes	0.452142888	0.257948545	-0.321702748	1.225988524	0.618926383	0.198666388	0.669770242	0.249510247	0.020369864	-0.399890131
5	135416382	cg06536614	**VTRNA2-1:DMR**	Yes	0.460966318	0.220924437	-0.201806993	1.123739629	0.627809734	0.195813685	0.647541439	0.21554539	0.044385493	-0.387610556
5	135416399	cg25340688	**VTRNA2-1:DMR**	Yes	0.477441164	0.238090799	-0.236831233	1.191713561	0.638701878	0.190120729	0.664468817	0.215887668	0.041863947	-0.406717202
5	135416406	cg26896946	**VTRNA2-1:DMR**	Yes	0.426319151	0.204479009	-0.187117877	1.03975618	0.559704657	0.159629985	0.603685882	0.20361121	0.043492295	-0.356582378
5	135416413	cg00124993	**VTRNA2-1:DMR**	Yes	0.335756115	0.181905597	-0.209960676	0.881472906	0.459372552	0.144658324	0.500525667	0.185811439	0.019757127	-0.294957101
5	135416530	cg08745965	**VTRNA2-1:DMR**	Yes	0.358637441	0.219907426	-0.301084835	1.018359718	0.479564761	0.148742323	0.511805861	0.180983423	0.013778302	-0.317044137
5	135416614	cg18797653	**VTRNA2-1:DMR**	Yes	0.528802933	0.248718865	-0.217353663	1.274959529	0.672740368	0.177604449	0.69872314	0.20358722	0.074021353	-0.421114567
6	3849096	cg18872973	**FAM50B:TSS-DMR**	Yes	0.82407114	0.027631785	0.741175785	0.906966495	0.832613323	0.044578502	0.766191422	-0.0218434	0.838887486	0.050852665
6	3849191	cg17739279	**FAM50B:TSS-DMR**	Yes	0.635964908	0.044775109	0.50163958	0.770290237	0.600720018	-0.010478812	0.683349772	0.072150941	0.636721995	0.025523164

6	3849236	cg18656763	**FAM50B:TSS-DMR**	Yes	0.53784909	0.022575283	0.47012324	0.605574941	0.535398555	0.020217365	0.559323398	0.044142209	0.515188658	7.46787E-06
6	3849278	cg09821214	**FAM50B:TSS-DMR**	Yes	0.585452991	0.043584953	0.454698131	0.716207851	0.572462283	0.014134775	0.605766117	0.047438608	0.575901329	0.017573821
6	3849295	cg07898446	**FAM50B:TSS-DMR**	Yes	0.508134453	0.026884946	0.427479615	0.58878929	0.49522841	0.008976506	0.506312153	0.020060248	0.524715507	0.038463602
6	3849328	cg25195497	**FAM50B:TSS-DMR**	Yes	0.621520819	0.064401112	0.428317482	0.814724156	0.538169286	-0.052740384	0.595665562	0.004755892	0.714204571	0.123294901
6	3849332	cg21740964	**FAM50B:TSS-DMR**	Yes	0.705492715	0.062572963	0.517773828	0.893211603	0.616375463	-0.055945901	0.675046933	0.002725569	0.789695019	0.117373654
6	3849351	cg13289019	**FAM50B:TSS-DMR**	Yes	0.409026031	0.02856111	0.3233427	0.494709362	0.394290662	0.001995934	0.434302706	0.042007978	0.381343894	-0.010950834
6	3849382	cg12840312	**FAM50B:TSS-DMR**	Yes	0.60359418	0.027210043	0.52196405	0.685224309	0.607372565	0.031048737	0.58920414	0.012880312	0.614157443	0.037833614
6	3849392	cg01905633	**FAM50B:TSS-DMR**	Yes	0.429264365	0.03226399	0.332472395	0.526056336	0.394643347	-0.01615129	0.428944691	0.018150054	0.437438399	0.026643762
6	3849412	cg21177626	**FAM50B:TSS-DMR**	Yes	0.450262272	0.035323503	0.344291764	0.556232781	0.441902416	0.012273573	0.457520616	0.027891773	0.438958551	0.009329708
6	3849435	cg03954573	**FAM50B:TSS-DMR**	Yes	0.652007368	0.034198807	0.549410947	0.754603789	0.617508766	-0.006051066	0.67436012	0.050800288	0.652152297	0.028592465
6	3849459	cg18197332	**FAM50B:TSS-DMR**	Yes	0.641425552	0.032627069	0.543544344	0.73930676	0.616541747	0.001909741	0.659988566	0.04535656	0.635514175	0.02088217
6	3849476	cg04447621	**FAM50B:TSS-DMR**	Yes	0.657520229	0.027456727	0.575150048	0.73989041	0.654744822	0.027526083	0.658940452	0.031721713	0.659798368	0.032579629
6	3849537	cg23835083	**FAM50B:TSS-DMR**	Yes	0.615446925	0.03794205	0.501620776	0.729273073	0.596924184	0.010223051	0.60932564	0.022624507	0.647449102	0.060747969
6	3849543	cg18487516	**FAM50B:TSS-DMR**	Yes	0.691977022	0.038117802	0.577623616	0.806330427	0.644771899	-0.015637585	0.714735267	0.054325782	0.722073217	0.061663732
6	3849578	cg12497786	**FAM50B:TSS-DMR**	Yes	0.725901074	0.038551276	0.610247247	0.841554901	0.732318158	0.036641704	0.783457969	0.087781514	0.675910516	-0.019765938
6	3849689	cg03202897	**FAM50B:TSS-DMR**	Yes	0.780217421	0.028970618	0.693305567	0.867129276	0.76162954	0.015345945	0.803318868	0.057035273	0.766694815	0.02041122
6	3849691	cg21153160	**FAM50B:TSS-DMR**	Yes	0.491336025	0.031622914	0.396467284	0.586204766	0.506873438	0.036739462	0.480099044	0.009965068	0.470918358	0.000784382
6	3849703	cg25073793	**FAM50B:TSS-DMR**	Yes	0.708470419	0.037421827	0.596204937	0.820735901	0.685889976	0.009700882	0.70851366	0.032324567	0.709167653	0.03297856
6	3849819	cg13101072	**FAM50B:TSS-DMR**	Yes	0.665586398	0.037788975	0.552219474	0.778953322	0.62324843	-0.013326744	0.674358779	0.037783605	0.692668599	0.056093425
6	3850107	cg05811173	**FAM50B:TSS-DMR**	Yes	0.612261177	0.02461867	0.538405168	0.686117186	0.602323121	0.015854433	0.63601541	0.049546722	0.586598413	0.000129725
6	144328422	cg09730369	**PLAGL1_alt:TSS-DMR**	Yes	0.824999225	0.028422511	0.739731693	0.910266756	0.821922667	0.033419714	0.88191313	0.093410177	0.790067982	0.001565029
6	144328483	cg17865602	**PLAGL1_alt:TSS-DMR**	Yes	0.652256521	0.042102638	0.525948606	0.778564436	0.651158908	0.029888415	0.667576141	0.046305648	0.644765569	0.023495077
6	144328918	cg25350411	**PLAGL1_alt:TSS-DMR**	Yes	0.575090912	0.050564191	0.423398339	0.726783484	0.517332597	-0.030851857	0.640508126	0.092323671	0.578176741	0.029992286
6	144329053	cg07077459	**PLAGL1_alt:TSS-DMR**	Yes	0.591378256	0.033723292	0.490208378	0.692548133	0.608900055	0.043996375	0.588290033	0.023386353	0.585177384	0.020273704
6	144329173	cg22378065	**PLAGL1_alt:TSS-DMR**	Yes	0.590858115	0.023252687	0.521100055	0.660616176	0.576435772	0.010777945	0.579132393	0.013474565	0.623561102	0.057903274
6	144329383	cg22352234	**PLAGL1_alt:TSS-DMR**	Yes	0.527594293	0.026210135	0.448963888	0.606224698	0.518723503	0.014975395	0.518626333	0.014878224	0.534660145	0.030912036
6	144329474	cg00702231	**PLAGL1_alt:TSS-DMR**	Yes	0.748503163	0.021690588	0.683431398	0.813574927	0.752963289	0.037459796	0.757449182	0.041945689	0.735358237	0.019854745
6	144329486	cg12757684	**PLAGL1_alt:TSS-DMR**	Yes	0.427599825	0.028520994	0.342036842	0.513162808	0.448212481	0.037565814	0.441923063	0.031276396	0.403453561	-0.007193106
6	144329745	cg12820609	**PLAGL1_alt:TSS-DMR**	NA	0.575947784	0.055283841	0.41009626	0.741799308	0.555769382	0.006002553	0.546662303	-0.003104526	0.566641769	0.01687494
6	144329767	cg05326984	**PLAGL1_alt:TSS-DMR**	Yes	0.652417564	0.022423826	0.585146086	0.719689041	0.645161961	0.01958266	0.657671168	0.032091867	0.660335366	0.034756065
6	144329781	cg08263357	**PLAGL1_alt:TSS-DMR**	Yes	0.674667016	0.05001545	0.524620668	0.824713365	0.672513926	0.029384771	0.687367518	0.044238362	0.651750485	0.008621329
6	144329790	cg11532302	**PLAGL1_alt:TSS-DMR**	Yes	0.530085394	0.04149318	0.405605853	0.654564934	0.524912859	0.019072668	0.546239586	0.040399395	0.495107575	-0.010732617
6	144329803	cg27216384	**PLAGL1_alt:TSS-DMR**	Yes	0.396527415	0.034573417	0.292807165	0.500247666	0.377155604	-0.003525982	0.439513726	0.058832141	0.343129831	-0.037551755
6	144329830	cg17895149	**PLAGL1_alt:TSS-DMR**	Yes	0.701449774	0.05582255	0.533982123	0.868917425	0.716831803	0.048564277	0.729329341	0.061061815	0.679855442	0.011587916
6	144329888	cg23460430	**PLAGL1_alt:TSS-DMR**	Yes	0.677569268	0.089802987	0.408160307	0.94697823	0.575776196	-0.069736913	0.582229507	-0.063283603	0.767959317	0.122446208
6	160427348	cg16039889	**IGF2R:Int2-DMR**	NA	0.641086255	0.048755204	0.494820641	0.787351868	0.615839978	0.004183769	0.682367362	0.070711154	0.638835659	0.027179451
6	160427502	cg08350488	**IGF2R:Int2-DMR**	Yes	0.617156674	0.101280215	0.313316029	0.920997319	0.643118502	0.055775414	0.591272426	0.003929338	0.69612311	0.108780023
6	168785356	cg25394782	SMOC2	Yes	0.909065662	0.028548278	0.823420828	0.994710497	0.911321175	0.040250834	0.937845748	0.066775407	0.844410244	-0.026660097
6	168785397	cg12758485	SMOC2	Yes	0.761577818	0.064751181	0.567324276	0.95583136	0.743471898	0.014693732	0.827358674	0.098580508	0.695241029	-0.033537138
6	168785525	cg01601658	SMOC2	Yes	0.271372517	0.047727498	0.128190023	0.414555012	0.255803732	-0.004417311	0.296783596	0.036562553	0.215339146	-0.044881897
6	170055156	cg11938672	**WDR27:Int13-DMR**	NA	0.762200171	0.101165636	0.458703262	1.06569708	0.786872487	0.054840402	0.782292228	0.050260143	0.773461437	0.041429352
6	170055333	cg18322025	**WDR27:Int13-DMR**	Yes	0.584065227	0.151137795	0.130651841	1.037478613	0.574394432	0.010757099	0.561504618	-0.002132715	0.616220893	0.05258356
7	1980480	cg13798585	MAD1L1	Yes	0.217015951	0.098741975	-0.079209975	0.513241877	0.195169332	-0.003427106	0.319198856	0.120602418	0.09589491	-0.102701528
7	1980809	cg26996890	MAD1L1	Yes	0.98323655	0.010843516	0.950706004	1.015767097	0.98845702	0.046528161	0.990309133	0.048380275	0.96021196	0.018283102
7	23530000	cg12829142	RPS2P32	Yes	0.330960321	0.060786735	0.148600115	0.513320527	0.260500747	-0.058891735	0.438115435	0.118722952	0.284277946	-0.035114537
7	23530811	cg16204552	RPS2P32	Yes	0.772546246	0.076659484	0.542567794	1.002524698	0.70528715	-0.032513226	0.85801177	0.120211395	0.810187317	0.072386942
7	50849169	cg03104936	**GRB10:alt-TSS-DMR**	Yes	0.684476213	0.048469819	0.539066756	0.829885669	0.653480866	0.000628174	0.717337245	0.064484553	0.692285843	0.03943315
7	50849286	cg05998930	**GRB10:alt-TSS-DMR**	Yes	0.805176378	0.054949936	0.64032657	0.970026185	0.772034797	0.004284218	0.753438684	-0.014311896	0.856997436	0.089246856
7	50849724	cg16349612	**GRB10:alt-TSS-DMR**	Yes	0.706153126	0.040314427	0.585209844	0.827096408	0.703475839	0.028910852	0.696650798	0.022085811	0.695489145	0.020924158
7	50849806	cg23742152	**GRB10:alt-TSS-DMR**	Yes	0.563215813	0.03457339	0.459495643	0.666935983	0.529338867	-0.008535225	0.580123942	0.042249851	0.585433919	0.047559828
7	50849932	cg08835688	**GRB10:alt-TSS-DMR**	Yes	0.619427123	0.039478933	0.500990324	0.737863922	0.627622532	0.035143007	0.617755849	0.025276323	0.603677743	0.011198217
7	50850565	cg12903171	**GRB10:alt-TSS-DMR**	Yes	0.597643454	0.045444756	0.461309185	0.733977723	0.621705488	0.051139728	0.552187312	-0.018378449	0.596702436	0.026136676
7	50850636	cg23023285	**GRB10:alt-TSS-DMR**	NA	0.623792364	0.036365832	0.514694868	0.73288986	0.629517805	0.033654191	0.618539434	0.02267582	0.62483315	0.028969536
7	50850756	cg27653667	**GRB10:alt-TSS-DMR**	NA	0.334368053	0.03905351	0.217207523	0.451528584	0.331839459	0.009475698	0.318550294	-0.003813468	0.333190586	0.010826824
7	50850871	cg24977055	**GRB10:alt-TSS-DMR**	Yes	0.63098496	0.041943849	0.505153412	0.756816509	0.624619604	0.02165047	0.642901421	0.039932288	0.635056087	0.032086953
7	94285643	cg19107595	**PEG10:TSS-DMR**	Yes	0.203674733	0.04295908	0.074797492	0.332551973	0.22865686	0.035319441	0.224360374	0.031022955	0.211654208	0.018316789
7	94285673	cg19924104	**PEG10:TSS-DMR**	Yes	0.394588889	0.033749679	0.293339852	0.495837926	0.360550365	-0.016769908	0.426470136	0.049149863	0.407673925	0.030353652
7	94285686	cg11589966	**PEG10:TSS-DMR**	Yes	0.547417276	0.033071775	0.44820195	0.646632601	0.55269573	0.028595115	0.557532268	0.033431654	0.506335028	-0.017765587
7	94285688	cg05531533	**PEG10:TSS-DMR**	NA	0.462695458	0.030965393	0.369799278	0.555591637	0.446168014	0.004141843	0.531746341	0.08972017	0.43130548	-0.01072069
7	94285713	cg27435646	**PEG10:TSS-DMR**	Yes	0.61713919	0.030596521	0.525349626	0.708928754	0.592410296	0.002466551	0.635563665	0.04561992	0.61630437	0.026360625
7	94285746	cg01566785	**PEG10:TSS-DMR**	Yes	0.633358453	0.023168565	0.56385276	0.702864147	0.605091844	-0.001758857	0.622347937	0.015497236	0.655701361	0.04885066
7	94285760	cg05819130	**PEG10:TSS-DMR**	Yes	0.624464673	0.033056772	0.525294356	0.72363499	0.57041036	-0.029527901	0.648723927	0.048785666	0.641359112	0.041420851
7	94285785	cg02965180	**PEG10:TSS-DMR**	Yes	0.568729847	0.024216795	0.496079462	0.641380233	0.545054157	0.001493536	0.583847302	0.040286681	0.554498877	0.010938255
7	94285815	cg05277165	**PEG10:TSS-DMR**	Yes	0.540635137	0.034160715	0.438152993	0.643117281	0.54368752	0.027405848	0.544740193	0.028458521	0.523691282	0.007409611
7	94285874	cg13660372	**PEG10:TSS-DMR**	Yes	0.567744301	0.032824922	0.469269535	0.666219067	0.554550582	0.009477501	0.57777254	0.032699458	0.539449917	-0.005623165
7	94285888	cg06674062	**PEG10:TSS-DMR**	Yes	0.52439507	0.043498155	0.393900606	0.654889534	0.514927868	0.01532561	0.499926762	0.000324503	0.551066621	0.051464363
7	94285912	cg07921535	**PEG10:TSS-DMR**	Yes	0.443986395	0.061003043	0.260977266	0.626995524	0.358043917	-0.062968743	0.448561887	0.027549226	0.473423781	0.052411121
7	94285943	cg09910998	**PEG10:TSS-DMR**	Yes	0.532952475	0.032790883	0.434579826	0.631325123	0.516936422	0.004757839	0.534230787	0.022052204	0.533880835	0.021702251
7	94285952	cg27393730	**PEG10:TSS-DMR**	Yes	0.31692582	0.041529583	0.19233707	0.44151457	0.309870022	0.005187053	0.346989569	0.0423066	0.298349963	-0.006333006
7	94285961	cg08291000	**PEG10:TSS-DMR**	Yes	0.619037181	0.042003134	0.493027779	0.745046583	0.605969425	0.015031947	0.653661382	0.062723904	0.578477263	-0.012460215
7	94285994	cg27230044	**PEG10:TSS-DMR**	Yes	0.626138853	0.065639436	0.429220543	0.823057162	0.576435863	-0.021615229	0.642392274	0.044341182	0.672849897	0.074798805
7	94286087	cg24885794	**PEG10:TSS-DMR**	Yes	0.549279379	0.044194663	0.41669539	0.681863369	0.550234344	0.027124538	0.560633645	0.037523839	0.542270298	0.019160492
7	94286111	cg26997085	**PEG10:TSS-DMR**	Yes	0.51686354	0.063231283	0.327169692	0.706557387	0.480132766	-0.011246689	0.520683178	0.029303723	0.564466833	0.073087378
7	94286132	cg22331138	**PEG10:TSS-DMR**	Yes	0.680439618	0.04597518	0.542514079	0.818365158	0.661737084	0.011989272	0.716523684	0.066775872	0.665116955	0.015369143
7	94286209	cg16492735	**PEG10:TSS-DMR**	Yes	0.701432387	0.044267246	0.56863065	0.834234124	0.687004994	0.015083204	0.736765766	0.064843976	0.703016278	0.031094488
7	94286220	cg09512080	**PEG10:TSS-DMR**	Yes	0.629125408	0.046653005	0.489166392	0.769084424	0.62976103	0.030851888	0.62929718	0.030388039	0.63904713	0.040137988
7	94286233	cg00906934	**PEG10:TSS-DMR**	Yes	0.617346537	0.057565926	0.444648758	0.790044316	0.630823612	0.043931894	0.622755427	0.03586371	0.607346905	0.020455187
7	94286244	cg26503018	**PEG10:TSS-DMR**	Yes	0.596059494	0.04579592	0.458671733	0.733447256	0.611205316	0.043703993	0.619854197	0.052352875	0.597124942	0.02962362
7	94286262	cg27120649	**PEG10:TSS-DMR**	Yes	0.420272623	0.032093616	0.323991775	0.516553472	0.405561692	0.001982874	0.424587806	0.021008988	0.436225737	0.032646918
7	94286264	cg21771834	**PEG10:TSS-DMR**	Yes	0.615854353	0.042269688	0.48904529	0.742663416	0.578710088	-0.007932555	0.654271193	0.06762855	0.633486561	0.046843918
7	94286268	cg27001184	**PEG10:TSS-DMR**	Yes	0.709408628	0.039177662	0.591875642	0.826941615	0.673484053	-0.003469487	0.736786815	0.059833276	0.725182198	0.048228658
7	94286295	cg23342787	**PEG10:TSS-DMR**	Yes	0.809105201	0.031992442	0.713127876	0.905082526	0.809960784	0.037121609	0.834927874	0.062088699	0.806026247	0.033187072
7	94286305	cg14873490	**PEG10:TSS-DMR**	Yes	0.529037346	0.037331228	0.41704366	0.641031031	0.521444128	0.017464062	0.519716098	0.015736032	0.531974259	0.027994193
7	94286344	cg03384175	**PEG10:TSS-DMR**	Yes	0.753846132	0.034651796	0.649890743	0.857801521	0.752555043	0.032176722	0.778677959	0.058299638	0.751734888	0.031356568
7	94286352	cg11985632	**PEG10:TSS-DMR**	Yes	0.681371507	0.03797315	0.567452058	0.795290957	0.679498587	0.029144747	0.717349016	0.066995176	0.703501861	0.053148021
7	94286363	cg13797824	**PEG10:TSS-DMR**	Yes	0.704456319	0.042972662	0.575538332	0.833374306	0.675436987	0.004949565	0.743738341	0.073250919	0.730007742	0.05952032
7	94286421	cg11175683	**PEG10:TSS-DMR**	Yes	0.650945247	0.051174613	0.497421409	0.804469085	0.636748752	0.015647171	0.676702776	0.055601195	0.664093787	0.042992206
7	94286432	cg04521244	**PEG10:TSS-DMR**	Yes	0.658816755	0.044930906	0.524024037	0.793609474	0.646086405	0.01767029	0.696683316	0.068267201	0.652848757	0.024432642
7	94286474	cg11562309	**PEG10:TSS-DMR**	Yes	0.573192326	0.060068744	0.392986094	0.753398558	0.549395633	0.004833339	0.614908217	0.070345923	0.583882516	0.039320222
7	94286485	cg18898992	**PEG10:TSS-DMR**	Yes	0.633659229	0.049235691	0.485952155	0.781366302	0.632339824	0.029383012	0.661441968	0.058485155	0.621375366	0.018418553
7	94286512	cg05096321	**PEG10:TSS-DMR**	Yes	0.59156645	0.031808027	0.496142368	0.686990531	0.562320462	-0.002469951	0.623225062	0.058434649	0.565389246	0.000598833
7	94286522	cg19663555	**PEG10:TSS-DMR**	Yes	0.7218919	0.040142154	0.601465437	0.842318364	0.723989459	0.034016096	0.735395378	0.045422016	0.705392506	0.015419144
7	94286527	cg06695761	**PEG10:TSS-DMR**	Yes	0.664937727	0.05044521	0.513602096	0.816273358	0.639774382	0.006819871	0.674380515	0.041426005	0.667905387	0.034950877
7	94286560	cg21853542	**PEG10:TSS-DMR**	Yes	0.767772456	0.040407008	0.646551431	0.88899348	0.789463433	0.057312532	0.775912856	0.043761954	0.732287135	0.000136234
7	94286651	cg09890341	**PEG10:TSS-DMR**	Yes	0.697669768	0.048113996	0.553327781	0.842011755	0.648633081	-0.015246601	0.767321318	0.103441636	0.691989593	0.028109911
7	94286670	cg20041873	**PEG10:TSS-DMR**	Yes	0.678139322	0.04615219	0.539682752	0.816595892	0.656800763	0.008831423	0.723950307	0.075980967	0.674814973	0.026845633
7	94286754	cg04303139	**PEG10:TSS-DMR**	Yes	0.555591917	0.042567513	0.427889378	0.683294455	0.533906284	0.004001163	0.54293581	0.01303069	0.563269364	0.033364243
7	94286761	cg03436478	**PEG10:TSS-DMR**	Yes	0.662751225	0.056487672	0.493288208	0.832214242	0.655095491	0.022649238	0.708814164	0.076367911	0.637941571	0.005495318
7	94286794	cg05509218	**PEG10:TSS-DMR**	Yes	0.740979538	0.041107502	0.617657031	0.864302046	0.683242292	-0.024944918	0.750588596	0.042401386	0.82575918	0.117571971
7	94286835	cg22924867	**PEG10:TSS-DMR**	Yes	0.632554931	0.043187913	0.502991193	0.762118669	0.605204438	0.000806647	0.661155948	0.056758157	0.635104224	0.030706433
7	94286937	cg01169624	**PEG10:TSS-DMR**	Yes	0.705367756	0.041645599	0.580430958	0.830304555	0.697110454	0.022076657	0.715963951	0.040930154	0.744296542	0.069262745
7	94287187	cg12397924	**PEG10:TSS-DMR**	Yes	0.433535357	0.03605005	0.325385208	0.541685506	0.417859631	0.000488092	0.410062096	-0.007309443	0.465129095	0.047757556
7	94287212	cg15696408	**PEG10:TSS-DMR**	Yes	0.288128129	0.030187018	0.197567076	0.378689182	0.277264348	-0.000323858	0.297020033	0.019431827	0.295886809	0.018298604
7	130130123	cg10473158	**MEST:alt-TSS-DMR**	Yes	0.800327968	0.055662546	0.633340331	0.967315606	0.649623009	-0.115939935	0.862701482	0.097138538	0.696501419	-0.069061525
7	130130156	cg05222671	**MEST:alt-TSS-DMR**	Yes	0.819071638	0.056341569	0.650046931	0.988096345	0.716987171	-0.068470417	0.877550323	0.092092736	0.673226694	-0.112230893
7	130130188	cg10767216	**MEST:alt-TSS-DMR**	Yes	0.731478184	0.045842713	0.593950045	0.869006323	0.597748673	-0.104344714	0.765726159	0.063632772	0.65293162	-0.049161767
7	130130204	cg15068552	**MEST:alt-TSS-DMR**	Yes	0.802478284	0.05713072	0.631086125	0.973870444	0.736374864	-0.034992199	0.875639614	0.104272551	0.678481077	-0.092885986
7	130130321	cg02756856	**MEST:alt-TSS-DMR**	Yes	0.811568183	0.051973635	0.655647279	0.967489086	0.732143453	-0.044092136	0.877826521	0.101590931	0.694804253	-0.081431336
7	130130384	cg26275543	**MEST:alt-TSS-DMR**	Yes	0.671723503	0.046794108	0.531341179	0.812105827	0.464228202	-0.179461118	0.773817504	0.130128183	0.586798405	-0.056890915
7	130130479	cg20826277	**MEST:alt-TSS-DMR**	Yes	0.686024598	0.047741244	0.542800866	0.829248329	0.585796044	-0.070402436	0.731960449	0.075761969	0.597252119	-0.058946361
7	130130482	cg14584935	**MEST:alt-TSS-DMR**	Yes	0.648008862	0.052487825	0.490545386	0.805472337	0.522442271	-0.096970846	0.717259985	0.097846868	0.550276979	-0.069136138
7	130130589	cg24941047	**MEST:alt-TSS-DMR**	Yes	0.902896215	0.027248091	0.821151941	0.984640489	0.902408883	0.037110303	0.941454701	0.076156121	0.872021503	0.006722923
7	130130741	cg02501418	**MEST:alt-TSS-DMR**	Yes	0.827618945	0.03486831	0.723014016	0.932223873	0.803115123	0.012449687	0.854451091	0.063785655	0.843253463	0.052588027
7	130130748	cg08229366	**MEST:alt-TSS-DMR**	NA	0.760139158	0.048479416	0.614700909	0.905577406	0.704929952	-0.022441331	0.776296911	0.048925628	0.799418503	0.072047221
7	130130754	cg07224147	**MEST:alt-TSS-DMR**	Yes	0.413360669	0.060960606	0.230478852	0.596242485	0.366338912	-0.031089792	0.382666056	-0.014762648	0.368107511	-0.029321193
7	130130919	cg27417677	**MEST:alt-TSS-DMR**	Yes	0.707045378	0.044294945	0.574160542	0.839930214	0.684662668	0.010419793	0.687832234	0.013589359	0.731523076	0.057280201

7	130130996	cg09003373	**MEST:alt-TSS-DMR**	Yes	0.482392528	0.045521314	0.345828587	0.61895647	0.515141976	0.054553011	0.488394964	0.027805999	0.433583519	-0.027005445
7	130131086	cg14088957	**MEST:alt-TSS-DMR**	Yes	0.504663356	0.054102194	0.342356776	0.666969937	0.470481614	-0.010655275	0.538110803	0.056973914	0.479866351	-0.001270537
7	130131137	cg06100421	**MEST:alt-TSS-DMR**	Yes	0.587355149	0.062229098	0.400667855	0.774042443	0.527930432	-0.031758176	0.588784861	0.029096254	0.619168312	0.059479704
7	130131139	cg20297423	**MEST:alt-TSS-DMR**	Yes	0.533387806	0.062371413	0.346273567	0.720502045	0.462737094	-0.04351693	0.581599494	0.07534547	0.548680998	0.042426974
7	130131147	cg05369791	**MEST:alt-TSS-DMR**	Yes	0.449586511	0.034164921	0.347091749	0.552081273	0.418975504	-0.014322353	0.410715718	-0.022582139	0.461391892	0.028094035
7	130131190	cg08734637	**MEST:alt-TSS-DMR**	Yes	0.515796888	0.029942084	0.425970637	0.605623138	0.520052477	0.026132021	0.487088273	-0.006832182	0.515117782	0.021197327
7	130131259	cg13917504	**MEST:alt-TSS-DMR**	Yes	0.629718376	0.049378189	0.481583809	0.777852942	0.591499823	-0.008685054	0.602972248	0.002787371	0.651518526	0.051333649
7	130131269	cg25407198	**MEST:alt-TSS-DMR**	NA	0.640321704	0.059529457	0.461733333	0.818910075	0.6128314	0.002621789	0.647424541	0.037214929	0.660828655	0.050619044
7	130131360	cg21667116	**MEST:alt-TSS-DMR**	Yes	0.598120371	0.064776578	0.403790638	0.792450103	0.553885575	-0.014866973	0.655369619	0.086617071	0.555252473	-0.013500076
7	130131368	cg23156962	**MEST:alt-TSS-DMR**	Yes	0.485096941	0.050007727	0.335073761	0.635120121	0.438756588	-0.022907082	0.469533863	0.007870193	0.498404096	0.036740426
7	130131404	cg04678950	**MEST:alt-TSS-DMR**	Yes	0.640733144	0.057686028	0.467675062	0.813791227	0.603059708	-0.011695607	0.685738517	0.070983202	0.644763755	0.030008441
7	130131481	cg17079325	**MEST:alt-TSS-DMR**	Yes	0.678424641	0.052604198	0.520612046	0.836237236	0.647041904	-0.00112922	0.679747828	0.031576704	0.693333281	0.045162156
7	130131485	cg04344875	**MEST:alt-TSS-DMR**	Yes	0.680245569	0.056322479	0.511278132	0.849213007	0.680397499	0.032888022	0.643769628	-0.00373985	0.694818616	0.047309139
7	130131634	cg00286878	**MEST:alt-TSS-DMR**	Yes	0.703728777	0.052532618	0.546130922	0.861326631	0.643851614	-0.027898067	0.723193111	0.05144343	0.697210747	0.025461067
7	130131677	cg12347392	**MEST:alt-TSS-DMR**	Yes	0.620856944	0.057359784	0.448777593	0.792936294	0.565220762	-0.029275978	0.628329869	0.033833129	0.654840673	0.060343933
7	130131692	cg04786207	**MEST:alt-TSS-DMR**	Yes	0.667798576	0.05512877	0.502412267	0.833184886	0.621247559	-0.015982645	0.694869625	0.057639421	0.686976146	0.049745941
7	130131731	cg26708559	**MEST:alt-TSS-DMR**	Yes	0.627338885	0.048668233	0.481334186	0.773343584	0.629650821	0.032338023	0.574661462	-0.022651336	0.656062926	0.058750128
7	130131798	cg22705386	**MEST:alt-TSS-DMR**	Yes	0.730361404	0.051594836	0.575576896	0.885145912	0.689329254	-0.011850375	0.786102829	0.084923199	0.719748963	0.018569334
7	130131827	cg06212135	**MEST:alt-TSS-DMR**	Yes	0.743822816	0.042710292	0.615691939	0.871953693	0.746575687	0.036919454	0.752080613	0.042424379	0.755682089	0.046025855
7	130131830	cg10249538	**MEST:alt-TSS-DMR**	Yes	0.725733303	0.035880582	0.618091556	0.83337505	0.720308445	0.02682842	0.740036933	0.046556907	0.717950899	0.024470874
7	130131870	cg16823958	**MEST:alt-TSS-DMR**	Yes	0.721226978	0.026799401	0.640828774	0.801625182	0.722594667	0.031413146	0.708839443	0.017657921	0.716661701	0.02548018
7	130131886	cg27338480	**MEST:alt-TSS-DMR**	Yes	0.674238326	0.036200619	0.565636468	0.782840184	0.644406748	0.000753359	0.654569093	0.010915705	0.697086824	0.053433436
7	130131888	cg09080913	**MEST:alt-TSS-DMR**	Yes	0.664927955	0.030771455	0.572613591	0.75724232	0.675493766	0.038653249	0.685628819	0.048788301	0.636325783	-0.000514734
7	130131906	cg13104298	**MEST:alt-TSS-DMR**	Yes	0.744763658	0.034208463	0.642138269	0.847389047	0.756648467	0.045663013	0.733785627	0.022800173	0.724433605	0.013448151
7	130131924	cg20050761	**MEST:alt-TSS-DMR**	Yes	0.534948293	0.029874757	0.445324021	0.624572566	0.522812906	0.011975782	0.512521697	0.001684573	0.538414291	0.027577167
7	130132162	cg05556276	**MEST:alt-TSS-DMR**	Yes	0.589823468	0.030485033	0.49836837	0.681278567	0.601471833	0.03686409	0.559851533	-0.00475621	0.605776036	0.041168293
7	130132200	cg17580798	**MEST:alt-TSS-DMR**	Yes	0.599897617	0.029065549	0.512700972	0.687094263	0.591357605	0.017767166	0.613425852	0.039835414	0.594428062	0.020837623
7	130132260	cg05862114	**MEST:alt-TSS-DMR**	Yes	0.296096805	0.062081076	0.109853576	0.482340034	0.225703629	-0.05843674	0.34206671	0.057926341	0.29049443	0.006354061
7	130132266	cg01784351	**MEST:alt-TSS-DMR**	Yes	0.496859031	0.062567216	0.309157382	0.684560679	0.493393659	0.020019066	0.513407774	0.040033182	0.45976875	-0.013605842
7	130132287	cg27589003	**MEST:alt-TSS-DMR**	Yes	0.487994421	0.043299502	0.358095916	0.617892927	0.504770181	0.03872033	0.520862933	0.054813082	0.450030666	-0.016019185
7	130132306	cg23714917	**MEST:alt-TSS-DMR**	Yes	0.709497711	0.036115503	0.601151202	0.817844219	0.699092089	0.019629235	0.708909438	0.029446584	0.705464678	0.026001823
7	130132361	cg25519926	**MEST:alt-TSS-DMR**	Yes	0.703900303	0.025643798	0.62696891	0.780831696	0.702777764	0.029802789	0.699027313	0.026052338	0.71613681	0.043161834
7	130132420	cg22592140	**MEST:alt-TSS-DMR**	Yes	0.619196405	0.054091229	0.456922718	0.781470092	0.559656154	-0.030837616	0.660489415	0.069995646	0.684290054	0.093796284
7	130132423	cg03588221	**MEST:alt-TSS-DMR**	Yes	0.658650186	0.047971025	0.514737111	0.80256326	0.650724154	0.022801584	0.657030729	0.029108158	0.652330939	0.024408369
7	130132454	cg13986840	**MEST:alt-TSS-DMR**	Yes	0.628341718	0.078168228	0.393837034	0.862846402	0.630333609	0.031547792	0.661795716	0.063009899	0.625169711	0.026383894
7	130133111	cg05260959	**MEST:alt-TSS-DMR**	Yes	0.822786403	0.04321256	0.693148722	0.952424083	0.831272155	0.04531193	0.824273447	0.038313222	0.819090763	0.033130539
7	130134257	cg18622506	**MEST:alt-TSS-DMR**	NA	0.381355417	0.104488411	0.067890184	0.694820649	0.37783401	0.004979066	0.484870949	0.112016005	0.226563662	-0.146291282
7	138348775	cg05719902	**SVOPL:alt-TSS-DMR**	Yes	0.510870941	0.077458459	0.278495562	0.743246319	0.439734585	-0.048183904	0.589033752	0.101115263	0.432159966	-0.055758524
7	138348982	cg16111924	**SVOPL:alt-TSS-DMR**	Yes	0.15075319	0.044087397	0.018491	0.28301538	0.125342576	-0.020925744	0.173898524	0.027630203	0.112868227	-0.033400093
7	154862771	cg25780543	**HTR5A:TSS-DMR**	Yes	0.327562263	0.048864166	0.180969765	0.474154761	0.273505592	-0.034878588	0.368866007	0.060481827	0.300887809	-0.007496371
7	154862970	cg00852398	**HTR5A:TSS-DMR**	Yes	0.617349042	0.031485363	0.522892953	0.711805131	0.572632212	-0.018053944	0.60326476	0.012578604	0.665310495	0.074624339
7	154863008	cg09125907	**HTR5A:TSS-DMR**	NA	0.670417758	0.043256604	0.540647946	0.800187571	0.629687013	-0.010074442	0.63263936	-0.007122095	0.718518003	0.078756548
7	154863177	cg25058023	**HTR5A:TSS-DMR**	Yes	0.600592813	0.046341996	0.461566826	0.7396188	0.547107773	-0.028564556	0.648275513	0.072603184	0.618495023	0.042822693
7	154863245	cg03024742	**HTR5A:TSS-DMR**	Yes	0.691826712	0.04108962	0.56855785	0.815095573	0.64674157	-0.014863303	0.709056973	0.0474521	0.678380963	0.01677609
7	154863324	cg13892088	**HTR5A:TSS-DMR**	NA	0.751467018	0.053283702	0.591615913	0.911318123	0.714597991	-0.001361547	0.762975438	0.0470159	0.755899188	0.03993965
7	154863339	cg10538202	**HTR5A:TSS-DMR**	Yes	0.791470693	0.047190699	0.649898595	0.93304279	0.706183287	-0.050767817	0.81816185	0.061210746	0.847608531	0.090657426
7	154863382	cg09623773	**HTR5A:TSS-DMR**	Yes	0.837878789	0.039839078	0.718361554	0.957396024	0.844222228	0.044649546	0.807875335	0.008302652	0.880258184	0.080685502
8	2585667	cg24475182	LOC101927815_1	Yes	0.280647499	0.083411793	0.03041212	0.530882879	0.2313669	-0.034609007	0.259262117	-0.006713791	0.338491893	0.072515985
8	2586226	cg11779808	LOC101927815_1	Yes	0.321377999	0.083372542	0.071260373	0.571495625	0.296390831	-0.005624769	0.335685433	0.033669832	0.311021936	0.009006336
8	2591208	cg04671734	LOC101927815_2	Yes	0.404824842	0.090542527	0.13319726	0.676452424	0.33158206	-0.051711742	0.461600318	0.078306516	0.417561787	0.034267985
8	2591412	cg08242633	LOC101927815_2	Yes	0.474296871	0.155429766	0.008007574	0.940586168	0.339798419	-0.106100118	0.608453608	0.162555071	0.464428695	0.018530158
8	37605553	cg05020125	**ERLIN2:Int6-DMR**	Yes	0.40920349	0.06589342	0.211523229	0.606883751	0.390436759	0.004882457	0.461442855	0.075888552	0.398883526	0.013329223
8	37605718	cg21505509	**ERLIN2:Int6-DMR**	Yes	0.581216556	0.064647413	0.387274318	0.775158793	0.590449528	0.038201414	0.626222776	0.073974662	0.53171272	-0.020535395
8	37605784	cg08247852	**ERLIN2:Int6-DMR**	Yes	0.605637444	0.087045394	0.344501263	0.866773626	0.529338759	-0.047377044	0.721949881	0.145234078	0.563542836	-0.013172967
8	37605979	cg00450319	**ERLIN2:Int6-DMR**	Yes	0.653967772	0.068290942	0.449094946	0.858840598	0.678573799	0.053647682	0.722579323	0.097653206	0.599334435	-0.025591682
8	61626647	cg12844324	CHD7	Yes	0.601211867	0.054487508	0.437749345	0.76467439	0.54597863	-0.028107719	0.63778278	0.063696431	0.658137651	0.084051302
8	61626833	cg20648501	CHD7	Yes	0.770214923	0.033141015	0.670791878	0.869637969	0.740938083	0.003998958	0.798974939	0.062035814	0.821549657	0.084610532
8	141108608	cg21926091	**PEG13:TSS-DMR**	Yes	0.77779599	0.02552439	0.701222819	0.854369161	0.767277286	0.022743504	0.796186496	0.051652714	0.785489713	0.04095593
8	141108999	cg22502459	**PEG13:TSS-DMR**	Yes	0.833430937	0.035683122	0.726381572	0.940480302	0.810510878	0.01372693	0.876112862	0.079328914	0.853503113	0.056719165
8	141109052	cg13713522	**PEG13:TSS-DMR**	Yes	0.808450282	0.025879756	0.730811014	0.886089551	0.800380698	0.025268019	0.844267461	0.069154782	0.783959639	0.00884696
8	141109629	cg17763909	**PEG13:TSS-DMR**	NA	0.785003816	0.029973291	0.695083942	0.874923689	0.756540277	0.005572591	0.81356674	0.062599054	0.790760579	0.039792894
8	141109732	cg07878171	**PEG13:TSS-DMR**	Yes	0.713113561	0.043806795	0.581693177	0.844533945	0.71923824	0.037829965	0.745100501	0.063692225	0.724034568	0.042626293
8	141110261	cg03803002	**PEG13:TSS-DMR**	Yes	0.738054192	0.033306989	0.638133226	0.837975159	0.708828318	0.003539554	0.77711579	0.071827026	0.727116466	0.021827702
8	141110748	cg00155882	**PEG13:TSS-DMR**	Yes	0.583395645	0.033082998	0.484146652	0.682644639	0.567280815	0.010364528	0.60887721	0.051960923	0.587283801	0.030367514
8	141110901	cg22807877	**PEG13:TSS-DMR**	Yes	0.571756219	0.035388223	0.465591551	0.677920888	0.507497178	-0.038099609	0.593747291	0.048150504	0.575766878	0.030170091
9	98075493	cg21891967	**FANCC:Int1-DMR**	Yes	0.515534447	0.077330887	0.283541786	0.747527107	0.440403962	-0.048968501	0.563706872	0.074334409	0.57459676	0.085224297
10	121578130	cg26352088	**INPP5F:Int2-DMR**	NA	0.791527307	0.03300809	0.692503036	0.890551578	0.796819938	0.038747444	0.820334157	0.062261664	0.814698111	0.056625617
10	121578138	cg02722214	**INPP5F:Int2-DMR**	Yes	0.809388526	0.022301169	0.742485019	0.876292032	0.820817065	0.045119651	0.853054595	0.077357181	0.831277158	0.055579744
10	121578385	cg20365618	**INPP5F:Int2-DMR**	Yes	0.615129881	0.040171504	0.494615368	0.735644394	0.587737859	-5.04798E-05	0.670944881	0.083156542	0.615995309	0.02820697
10	121578562	cg02857557	**INPP5F:Int2-DMR**	Yes	0.838796132	0.026242682	0.760068085	0.917524178	0.814779764	0.011975618	0.904171518	0.101367373	0.850683666	0.047879521
10	121578626	cg01729557	**INPP5F:Int2-DMR**	NA	0.846304127	0.030454291	0.754941254	0.937666999	0.84750987	0.036945223	0.879167288	0.068602641	0.862110406	0.051545759
10	121578640	cg25045785	**INPP5F:Int2-DMR**	Yes	0.847709099	0.023959917	0.775829348	0.91958885	0.833737371	0.022896702	0.882660694	0.071820024	0.858131635	0.047290965
11	2019080	cg11753499	**H19-IGF2:IG-DMR**	Yes	0.614292324	0.052836844	0.455781792	0.772802855	0.272328797	-0.314095537	0.671439879	0.085015545	0.654020473	0.067596139
11	2019091	cg13210239	**H19-IGF2:IG-DMR**	Yes	0.706626903	0.035262306	0.600839986	0.81241382	0.427027283	-0.248817626	0.78534617	0.109501261	0.747694345	0.071849436
11	2019117	cg17985533	**H19-IGF2:IG-DMR**	Yes	0.641988592	0.057642924	0.46905982	0.814917364	0.366906993	-0.250562573	0.694731665	0.077262099	0.68470175	0.067232184
11	2019130	cg15922305	**H19-IGF2:IG-DMR**	Yes	0.690223341	0.049176255	0.542694574	0.837752107	0.456176481	-0.205953198	0.787181153	0.125051475	0.707376318	0.045246639
11	2019437	cg09701145	**H19-IGF2:IG-DMR**	Yes	0.668964349	0.038786149	0.552605902	0.785322795	0.387798941	-0.252063555	0.717367968	0.077505472	0.695867741	0.056005245
11	2019453	cg25437674	**H19-IGF2:IG-DMR**	NA	0.533312323	0.070336919	0.322301565	0.744323081	0.214433681	-0.293156031	0.555413256	0.047823544	0.563224229	0.055634516
11	2019569	cg17769238	**H19-IGF2:IG-DMR**	Yes	0.691573177	0.04815364	0.547112256	0.836034097	0.442992733	-0.217610912	0.763756356	0.103152712	0.724869901	0.064266257
11	2019588	cg04975775	**H19-IGF2:IG-DMR**	Yes	0.671469872	0.070091191	0.461196297	0.881743446	0.388586417	-0.260631807	0.769380265	0.120162041	0.642217844	-0.00700038
11	2019607	cg15886040	**H19-IGF2:IG-DMR**	Yes	0.632627381	0.075159569	0.407148673	0.858106088	0.398871943	-0.214499398	0.711276186	0.097904846	0.624617649	0.011246308
11	2019625	cg10154633	**H19-IGF2:IG-DMR**	Yes	0.669685758	0.034082172	0.567439243	0.771932273	0.507596192	-0.133085548	0.702932764	0.062251023	0.702570591	0.06188885
11	2019627	cg26469586	**H19-IGF2:IG-DMR**	Yes	0.619271895	0.055671038	0.452258779	0.78628501	0.429728875	-0.159663894	0.654987595	0.065594827	0.666091996	0.076699228
11	2019656	cg24409677	**H19-IGF2:IG-DMR**	Yes	0.601865675	0.029498728	0.513369492	0.690361859	0.393713752	-0.183493314	0.64687422	0.069667154	0.633969997	0.056762931
11	2019668	cg16303279	**H19-IGF2:IG-DMR**	Yes	0.670808871	0.030559502	0.579130364	0.762487379	0.454941386	-0.188228347	0.721971785	0.078802053	0.694222842	0.05105311
11	2019731	cg02694715	**H19-IGF2:IG-DMR**	Yes	0.630240733	0.023786268	0.558881931	0.701599536	0.541567688	-0.062913899	0.649729029	0.045247442	0.658506411	0.054024824
11	2019733	cg11735853	**H19-IGF2:IG-DMR**	Yes	0.528574458	0.039358356	0.41049939	0.646649525	0.371129092	-0.132844079	0.570189626	0.066216455	0.551490492	0.047517321
11	2019737	cg01977486	**H19-IGF2:IG-DMR**	Yes	0.785702427	0.044313342	0.652762402	0.918642452	0.610276688	-0.140036656	0.818387501	0.068074157	0.822033057	0.071719713
11	2019824	cg13581483	**H19-IGF2:IG-DMR**	Yes	0.659705694	0.029080734	0.572463493	0.746947896	0.495950298	-0.134899337	0.69397998	0.063130345	0.685116231	0.054266596
11	2019863	cg01539474	**H19-IGF2:IG-DMR**	Yes	0.778570311	0.041617502	0.653717805	0.903422816	0.498220056	-0.245245791	0.829468348	0.086002501	0.813160472	0.069694625
11	2019931	cg18362496	**H19-IGF2:IG-DMR**	Yes	0.689845697	0.062287245	0.502983963	0.876707431	0.42207331	-0.234529037	0.698323989	0.041721642	0.746843357	0.090241011
11	2019944	cg24605090	**H19-IGF2:IG-DMR**	Yes	0.763592908	0.053788461	0.602227525	0.924958291	0.537605669	-0.189515886	0.788887732	0.061766177	0.805575751	0.078454196
11	2020102	cg16675558	**H19-IGF2:IG-DMR**	Yes	0.927295737	0.028545292	0.841659862	1.012931612	0.853989755	-0.032891932	0.93214319	0.045261502	0.962380925	0.075499238
11	2020105	cg03996735	**H19-IGF2:IG-DMR**	Yes	0.857077378	0.045471582	0.720662634	0.993492123	0.724298616	-0.093088971	0.86440948	0.047021892	0.915657164	0.098269577
11	2020119	cg18104242	**H19-IGF2:IG-DMR**	Yes	0.895211234	0.031191936	0.801635426	0.988787043	0.815642847	-0.039633672	0.890793568	0.035517049	0.941592005	0.086315486
11	2020130	cg27300742	**H19-IGF2:IG-DMR**	Yes	0.715445754	0.070028775	0.50535943	0.925532078	0.48951229	-0.190635331	0.727984742	0.047837121	0.79206404	0.111916419
11	2020280	cg25281616	**H19-IGF2:IG-DMR**	Yes	0.753797388	0.053174715	0.594273241	0.913321534	0.574770053	-0.143234949	0.74671576	0.028710757	0.803287261	0.085282259
11	2020287	cg01895612	**H19-IGF2:IG-DMR**	Yes	0.641143364	0.060895988	0.458455399	0.823831328	0.474982773	-0.133913213	0.606725358	-0.002170628	0.704864931	0.095968944
11	2020297	cg23476401	**H19-IGF2:IG-DMR**	Yes	0.738365068	0.072712085	0.520228814	0.956501322	0.484720489	-0.217018149	0.74834082	0.046602183	0.824079837	0.1223412
11	2020315	cg00237904	**H19-IGF2:IG-DMR**	Yes	0.684506305	0.067883264	0.480856514	0.888156096	0.402634465	-0.247036656	0.704434102	0.054762981	0.778385946	0.128714825
11	2020418	cg25821896	**H19-IGF2:IG-DMR**	Yes	0.677203424	0.053265946	0.517405584	0.837001263	0.452691169	-0.191823495	0.711163308	0.066648644	0.7280288	0.083514135
11	2020538	cg18454954	**H19-IGF2:IG-DMR**	Yes	0.604624933	0.052071519	0.448410376	0.760839489	0.442880522	-0.133658427	0.610264198	0.033725249	0.625395893	0.048856944
11	2020550	cg25579157	**H19-IGF2:IG-DMR**	Yes	0.577365043	0.024067768	0.50516174	0.649568346	0.504997507	-0.047192303	0.573446424	0.021256614	0.607314134	0.055124324
11	2020557	cg02886509	**H19-IGF2:IG-DMR**	Yes	0.617660446	0.059181236	0.440116737	0.795204155	0.406123948	-0.182243799	0.631925116	0.043557369	0.699210519	0.110842772
11	2020561	cg02657360	**H19-IGF2:IG-DMR**	Yes	0.633632533	0.061530496	0.449041045	0.81822402	0.396247382	-0.205733212	0.639670262	0.037689668	0.686382306	0.084401711
11	2022325	cg16574793	**H19-IGF2:IG-DMR**	Yes	0.663729345	0.047029121	0.522641981	0.804816709	0.618194257	-0.015353038	0.647267359	0.013720063	0.692327641	0.058780345
11	2022387	cg09452478	**H19-IGF2:IG-DMR**	NA	0.561487719	0.047931317	0.417693767	0.705281671	0.525971367	-0.009047685	0.520731963	-0.014287089	0.601004026	0.065984974
11	2022749	cg06658831	**H19-IGF2:IG-DMR**	NA	0.635445501	0.058178036	0.460911392	0.80997961	0.575240284	-0.029917107	0.605146875	-1.05164E-05	0.657976476	0.052819085
11	2024127	cg19024989	**H19-IGF2:IG-DMR**	Yes	0.695611661	0.034989765	0.590642365	0.800580957	0.645230761	-0.02014415	0.702713974	0.037339063	0.721244214	0.055869302
11	2153992	cg02807948	**IGF2:Ex9-DMR**	Yes	0.567798738	0.05421246	0.405161358	0.730436119	0.510063267	-0.027651015	0.565073782	0.0273595	0.58203873	0.044324448
11	2154075	cg12528452	**IGF2:Ex9-DMR**	NA	0.735161031	0.058268515	0.560355485	0.909966576	0.594949047	-0.111975104	0.734136521	0.02721237	0.798603804	0.091679653
11	2154114	cg13165070	**IGF2:Ex9-DMR**	Yes	0.301418007	0.038978604	0.184482195	0.418353819	0.210661716	-0.07928091	0.285769367	-0.00417326	0.329755928	0.039813302
11	2154133	cg11717189	**IGF2:Ex9-DMR**	Yes	0.445345174	0.067939335	0.241527169	0.649163178	0.26788426	-0.155562804	0.432306619	0.008859556	0.407845485	-0.015601579
11	2154256	cg07096953	**IGF2:Ex9-DMR**	Yes	0.298449271	0.043449021	0.168102208	0.428796333	0.174787483	-0.11197567	0.325971398	0.039208245	0.313015786	0.026252633
11	2154387	cg02613624	**IGF2:Ex9-DMR**	Yes	0.867551671	0.022539316	0.799933723	0.93516962	0.794486865	-0.034670011	0.857564148	0.028407272	0.875910602	0.046753726
11	2154433	cg22956483	**IGF2:Ex9-DMR**	Yes	0.271253028	0.035680819	0.164210571	0.378295486	0.177438906	-0.080577952	0.297115577	0.039098719	0.280181118	0.02216426

11	2154639	cg26401390	**IGF2:Ex9-DMR**	Yes	0.624133737	0.047608883	0.481307089	0.766960384	0.49795635	-0.098691699	0.615839849	0.0191918	0.645203796	0.048555747
11	2154935	cg05384664	**IGF2:Ex9-DMR**	Yes	0.799534796	0.038518402	0.68397959	0.915090003	0.700636237	-0.063194859	0.793256206	0.02942511	0.812734621	0.048903526
11	2154953	cg00570518	**IGF2:Ex9-DMR**	Yes	0.803937017	0.038896351	0.687247964	0.920626071	0.703227822	-0.064451746	0.797461879	0.029782312	0.826183772	0.058504205
11	2168626	cg19642877	**IGF2:alt-TSS-DMR**	Yes	0.626156499	0.038474206	0.510733881	0.741579118	0.474894959	-0.125084493	0.612711763	0.01273231	0.687694134	0.087714682
11	2169139	cg20622142	**IGF2:alt-TSS-DMR**	NA	0.34911607	0.047312163	0.20717958	0.491052559	0.240692054	-0.093239338	0.361224407	0.027293016	0.373672994	0.039741603
11	2720230	cg27119222	**KCNQ1OT1:TSS-DMR**	Yes	0.695874486	0.058219796	0.521215099	0.870533874	0.666578781	0.001125468	0.70752442	0.042071107	0.709430957	0.043977645
11	2720464	cg00000924	**KCNQ1OT1:TSS-DMR**	Yes	0.626726151	0.05889633	0.450037161	0.803415141	0.614619963	0.018447833	0.622587141	0.026415012	0.628618726	0.032446597
11	2720811	cg11297256	**KCNQ1OT1:TSS-DMR**	Yes	0.288397648	0.031734786	0.193193289	0.383602008	0.268877311	-0.009412668	0.288720625	0.010430646	0.29204008	0.013750101
11	2721244	cg12077660	**KCNQ1OT1:TSS-DMR**	Yes	0.560319753	0.024279986	0.487479795	0.633159711	0.56031266	0.022791459	0.559098015	0.021576814	0.537593295	7.20938E-05
11	2721249	cg03401726	**KCNQ1OT1:TSS-DMR**	Yes	0.616704419	0.031772528	0.521386835	0.712022002	0.59530654	0.006462815	0.605396503	0.016552778	0.644364801	0.055521076
11	2721337	cg16739686	**KCNQ1OT1:TSS-DMR**	Yes	0.59072947	0.036684473	0.480676051	0.70078289	0.560543193	-0.003383786	0.629522618	0.065595638	0.569033958	0.005106979
11	2721352	cg08359167	**KCNQ1OT1:TSS-DMR**	Yes	0.561870618	0.03363691	0.460959889	0.662781348	0.547597293	0.009824566	0.564125103	0.026352376	0.55557105	0.017798322
11	2721367	cg08446215	**KCNQ1OT1:TSS-DMR**	Yes	0.723766907	0.031698784	0.628670555	0.818863258	0.676010916	-0.016992161	0.751393553	0.058390475	0.735458323	0.042455246
11	2721384	cg26104781	**KCNQ1OT1:TSS-DMR**	Yes	0.738156416	0.036683645	0.628105482	0.84820735	0.748059992	0.042116878	0.750348996	0.044405881	0.720420703	0.014477588
11	2721410	cg02219360	**KCNQ1OT1:TSS-DMR**	Yes	0.572634917	0.031001662	0.479629931	0.665639903	0.544162207	-0.006500051	0.582510849	0.031848591	0.599843197	0.049180939
11	2721481	cg07595203	**KCNQ1OT1:TSS-DMR**	Yes	0.549505965	0.033290668	0.449633959	0.64937797	0.535480879	0.010156592	0.573501137	0.048176851	0.525450215	0.000125928
11	2721592	cg09518720	**KCNQ1OT1:TSS-DMR**	Yes	0.683415336	0.040056664	0.563245345	0.803585328	0.657659801	0.003102376	0.707898241	0.053340816	0.672132478	0.017575054
11	2721611	cg14958441	**KCNQ1OT1:TSS-DMR**	Yes	0.622536386	0.048757316	0.476264438	0.768808334	0.60273029	0.009092134	0.594727047	0.001088892	0.646912845	0.05327469
11	2721620	cg27323091	**KCNQ1OT1:TSS-DMR**	Yes	0.590208554	0.029585779	0.501451216	0.678965892	0.586432307	0.021153326	0.585185055	0.019906074	0.624536173	0.059257192
11	2721633	cg01873334	**KCNQ1OT1:TSS-DMR**	Yes	0.790802681	0.03202609	0.694724411	0.886880952	0.786736587	0.029394918	0.795638294	0.038296625	0.804754024	0.047412355
11	2721800	cg05816130	**KCNQ1OT1:TSS-DMR**	Yes	0.672161266	0.047350864	0.530108674	0.814213859	0.683255411	0.042014833	0.630493648	-0.010746929	0.672648435	0.031407858
11	2721818	cg14243741	**KCNQ1OT1:TSS-DMR**	Yes	0.689502237	0.050599928	0.537702453	0.841302021	0.654216335	-0.002447962	0.673946815	0.017282517	0.707278766	0.050614469
11	2721867	cg05740879	**KCNQ1OT1:TSS-DMR**	Yes	0.758476649	0.038230329	0.643785661	0.873167636	0.745018407	0.020271697	0.744999894	0.020253185	0.776452987	0.051706277
11	2721953	cg01893176	**KCNQ1OT1:TSS-DMR**	Yes	0.641189608	0.041111328	0.517855625	0.76452359	0.597906058	-0.014843136	0.630723849	0.017974656	0.675670909	0.062921716
11	2722063	cg11666921	**KCNQ1OT1:TSS-DMR**	Yes	0.482355025	0.066715405	0.282208809	0.682501241	0.491748361	0.029628983	0.479353027	0.017233649	0.485982383	0.023863005
11	2722074	cg26094482	**KCNQ1OT1:TSS-DMR**	Yes	0.412978289	0.030533353	0.321378231	0.504578346	0.381419395	-0.013366427	0.449830254	0.055044433	0.411671395	0.016885574
11	2722083	cg03422070	**KCNQ1OT1:TSS-DMR**	Yes	0.727294196	0.044260834	0.594511696	0.860076697	0.707107406	0.013322814	0.719232027	0.025447435	0.743369779	0.049585187
11	2722085	cg15651941	**KCNQ1OT1:TSS-DMR**	Yes	0.572427078	0.049100929	0.425124291	0.719729865	0.548647031	0.004755236	0.581661744	0.037769949	0.588145352	0.044253557
11	2722087	cg25306939	**KCNQ1OT1:TSS-DMR**	Yes	0.641419296	0.058709275	0.465291472	0.817547121	0.635655229	0.024549497	0.630350026	0.019244295	0.64836328	0.037257548
11	2722196	cg02798157	**KCNQ1OT1:TSS-DMR**	Yes	0.560176788	0.061312426	0.376239509	0.744114067	0.578423522	0.046161745	0.477342676	-0.054919102	0.58260481	0.050343033
13	48892552	cg27182551	**RB1:Int2-DMR**	Yes	0.853631005	0.037333086	0.741631747	0.965630262	0.840576292	0.022591312	0.902789511	0.084804531	0.833406067	0.015421087
13	48893175	cg13431205	**RB1:Int2-DMR**	Yes	0.608992367	0.044145588	0.476555604	0.741429131	0.57097644	-0.011662871	0.644819142	0.062179832	0.59984047	0.017201159
13	48893728	cg18481241	**RB1:Int2-DMR**	Yes	0.550594731	0.049919765	0.400835436	0.700354027	0.54854552	0.023590635	0.60686865	0.081913766	0.497824465	-0.027130419
13	48894215	cg25676335	**RB1:Int2-DMR**	Yes	0.812176439	0.030310516	0.721244891	0.903107987	0.801224278	0.024389713	0.81750635	0.040671785	0.819615001	0.042780436
13	48894383	cg00059930	**RB1:Int2-DMR**	Yes	0.600681878	0.043981373	0.468737759	0.732625996	0.581799061	0.008498115	0.639077854	0.065776908	0.58278797	0.009487024
13	48895242	cg26740195	**RB1:Int2-DMR**	Yes	0.632471036	0.047378263	0.490336247	0.774605826	0.5922683	-0.011650142	0.641201931	0.037283489	0.638354565	0.034436122
13	48895319	cg27026713	**RB1:Int2-DMR**	Yes	0.689014522	0.038116738	0.574664307	0.803364737	0.706330026	0.04481863	0.656651523	-0.004859873	0.740745247	0.079233851
13	48895479	cg07880715	**RB1:Int2-DMR**	Yes	0.748542407	0.037870997	0.634929415	0.862155398	0.720618433	0.003764071	0.77842871	0.061574347	0.731972302	0.01511794
13	111062248	cg18114040	COL4A2	Yes	0.284940753	0.048297697	0.140047661	0.429833845	0.276160921	0.002937477	0.331670614	0.05844717	0.228830719	-0.044392726
13	111062267	cg08959039	COL4A2	Yes	0.57236764	0.056967336	0.401465632	0.743269648	0.580390233	0.031001161	0.532710068	-0.016679004	0.56777764	0.018388567
13	111062288	cg11096515	COL4A2	Yes	0.584306978	0.074274679	0.361482941	0.807131014	0.615013392	0.058689743	0.59713267	0.040809021	0.51384438	-0.042479269
13	115000150	cg21025488	CDC16	Yes	0.105154491	0.039295552	-0.012732164	0.223041145	0.066716959	-0.03350561	0.137541887	0.037319318	0.100959222	0.000736653
13	115000163	cg24890222	CDC16	Yes	0.105150346	0.037879037	-0.008486765	0.218787456	0.056831163	-0.040268369	0.117859734	0.020760202	0.098042794	0.000943262
14	101192853	cg14092276	DLK1	Yes	0.558303518	0.049473282	0.409883672	0.706723363	0.456631063	-0.079590217	0.632752088	0.096530808	0.529487285	-0.006733995
14	101192861	cg18373878	DLK1	Yes	0.752427998	0.041084023	0.629175928	0.875680068	0.675986259	-0.048468481	0.77723195	0.052777209	0.770610626	0.046155885
14	101192914	cg10528576	DLK1	Yes	0.4755124	0.077920055	0.241752235	0.709272564	0.42517545	-0.037608607	0.524730629	0.061946572	0.471794367	0.009010311
14	101277287	cg27281850	**MEG3-DLK1:IG_DMR**	NA	0.791294669	0.048601408	0.645490445	0.937098894	0.627883398	-0.127089546	0.822738578	0.067765634	0.802284183	0.047311238
14	101290557	cg00764369	**MEG3:TSS-DMR**	Yes	0.810857518	0.028694279	0.724774682	0.896940353	0.632758546	-0.142967948	0.834938197	0.059211703	0.832578239	0.056851745
14	101290718	cg10515315	**MEG3:TSS-DMR**	Yes	0.552500248	0.060786867	0.370139646	0.73486085	0.192813367	-0.331904042	0.5894691	0.064751691	0.591952251	0.067234842
14	101290868	cg15991553	**MEG3:TSS-DMR**	Yes	0.649692062	0.056326184	0.480713511	0.818670613	0.208640931	-0.41224408	0.68181404	0.060929028	0.689537454	0.068652442
14	101291069	cg23870378	**MEG3:TSS-DMR**	Yes	0.602207596	0.055399853	0.436008036	0.768407156	0.359887204	-0.214884837	0.667368544	0.092596502	0.633791895	0.059019854
14	101291101	cg14034270	**MEG3:TSS-DMR**	Yes	0.872768976	0.026260372	0.79398786	0.951550091	0.782945027	-0.052253841	0.894684495	0.059485628	0.900703675	0.065504807
14	101291137	cg16567044	**MEG3:TSS-DMR**	Yes	0.56109419	0.053411908	0.400858466	0.721329913	0.449179497	-0.085170162	0.549234385	0.014884726	0.6196205	0.085270842
14	101291181	cg10540302	**MEG3:TSS-DMR**	Yes	0.676985657	0.044632079	0.543089419	0.810881895	0.541495894	-0.104418918	0.669679925	0.023765113	0.709013086	0.063098274
14	101291289	cg02313552	**MEG3:TSS-DMR**	Yes	0.675593147	0.044204628	0.542979264	0.80820703	0.465743214	-0.179293256	0.704912968	0.059876498	0.688341957	0.043305487
14	101291411	cg02982753	**MEG3:TSS-DMR**	Yes	0.594414973	0.05214721	0.437973344	0.750856602	0.244673827	-0.321526268	0.638920475	0.072720379	0.63718287	0.070982775
14	101291441	cg02888166	**MEG3:TSS-DMR**	Yes	0.698083187	0.028117409	0.613730958	0.782435415	0.580193251	-0.086810645	0.70808961	0.041085714	0.716153112	0.049149216
14	101291460	cg24089209	**MEG3:TSS-DMR**	Yes	0.698255301	0.046099211	0.559957668	0.836552933	0.498181581	-0.166268499	0.729850822	0.065400742	0.735160036	0.070709956
14	101291470	cg16738180	**MEG3:TSS-DMR**	Yes	0.717563153	0.038591348	0.601789109	0.833337197	0.589274532	-0.095357851	0.733363138	0.048730755	0.749173875	0.064541492
14	101291501	cg19643803	**MEG3:TSS-DMR**	Yes	0.695871083	0.047297287	0.553979222	0.837762944	0.425481439	-0.23867903	0.729148919	0.06498845	0.720500643	0.056340174
14	101291603	cg10065153	**MEG3:TSS-DMR**	Yes	0.681285904	0.063112484	0.491948452	0.870623355	0.399077011	-0.25193523	0.731508888	0.080496647	0.721806198	0.070793957
14	101291688	cg09280976	**MEG3:TSS-DMR**	Yes	0.8097256	0.030083734	0.719474398	0.899976803	0.673687013	-0.100171085	0.823718313	0.049860215	0.846777461	0.072919362
14	101291830	cg22940816	**MEG3:TSS-DMR**	Yes	0.547922223	0.054242677	0.385194193	0.710650253	0.222789746	-0.299243034	0.592024164	0.069991385	0.603626245	0.081593466
14	101291857	cg14070323	**MEG3:TSS-DMR**	Yes	0.722023512	0.024506277	0.64850468	0.795542344	0.254397565	-0.438149568	0.770669241	0.078122108	0.769075028	0.076527895
14	101291892	cg14119337	**MEG3:TSS-DMR**	Yes	0.460721868	0.038241505	0.345997352	0.575446384	0.153327411	-0.286673161	0.492119618	0.052119047	0.506804098	0.066803527
14	101291934	cg20952167	**MEG3:TSS-DMR**	Yes	0.504100359	0.026859921	0.423520597	0.584680122	0.162550345	-0.320345257	0.552059317	0.069163715	0.558481676	0.075586074
14	101291998	cg12967319	**MEG3:TSS-DMR**	Yes	0.594684084	0.048485245	0.449228347	0.74013982	0.156272411	-0.409414955	0.645120415	0.079433049	0.656124008	0.090436642
14	101292129	cg04304932	**MEG3:TSS-DMR**	Yes	0.678755203	0.019171606	0.621240384	0.736270022	0.17744661	-0.471891117	0.736560064	0.087222336	0.733858714	0.084520987
14	101292150	cg04576764	**MEG3:TSS-DMR**	Yes	0.679372297	0.025410687	0.603140237	0.755604357	0.227451411	-0.421626104	0.725618904	0.076541389	0.734348469	0.085270954
14	101292307	cg25836301	**MEG3:TSS-DMR**	Yes	0.683827857	0.04589471	0.546143727	0.821511988	0.204624986	-0.449968721	0.725855355	0.071261648	0.743540532	0.088946826
14	101292393	cg09926418	**MEG3:TSS-DMR**	Yes	0.660511796	0.057327972	0.488527881	0.832495711	0.131813814	-0.497213428	0.723507862	0.09448062	0.730145586	0.101118344
14	101292420	cg14759154	**MEG3:TSS-DMR**	NA	0.749730951	0.048022028	0.605664867	0.893797036	0.209775587	-0.505087238	0.803334523	0.088471698	0.8163183	0.101455474
14	101292644	cg23176399	**MEG3:TSS-DMR**	Yes	0.692923196	0.036941071	0.582099984	0.803746408	0.232075299	-0.429828528	0.734204384	0.072300556	0.756605216	0.094701388
14	101292681	cg05711886	**MEG3:TSS-DMR**	Yes	0.640114551	0.034291276	0.537240724	0.742988378	0.159030667	-0.453736143	0.69825324	0.08548643	0.703168717	0.090401907
14	101292873	cg15419911	**MEG3:TSS-DMR**	Yes	0.673276234	0.032898914	0.574579491	0.771972976	0.228332365	-0.414319852	0.740649497	0.09799728	0.735563214	0.092910997
14	101292968	cg14245102	**MEG3:TSS-DMR**	Yes	0.538763833	0.0411861	0.415205534	0.662322132	0.127886229	-0.387105256	0.623593878	0.108602393	0.612680765	0.09768928
14	101293091	cg15373285	**MEG3:TSS-DMR**	Yes	0.606011802	0.055858729	0.438435615	0.773587989	0.150396429	-0.426692307	0.641352836	0.064264101	0.660816675	0.08372794
14	101293451	cg14123427	**MEG3:TSS-DMR**	Yes	0.537941872	0.05040792	0.386718111	0.689165633	0.279636545	-0.230781468	0.545730577	0.035312565	0.563348502	0.05293049
14	101293636	cg00743158	**MEG3:TSS-DMR**	NA	0.752864478	0.037291873	0.640988859	0.864740096	0.207609632	-0.511938169	0.808995791	0.08944799	0.807890438	0.088342638
14	101293646	cg09291977	**MEG3:TSS-DMR**	NA	0.680508722	0.05145763	0.526135832	0.834881611	0.101506732	-0.545583941	0.752393634	0.105302961	0.752251835	0.105161162
14	101293727	cg15101633	**MEG3:TSS-DMR**	Yes	0.667157445	0.047712495	0.524019961	0.810294929	0.280663886	-0.355163722	0.674548324	0.038720717	0.699292661	0.063465054
14	101293857	cg26374305	**MEG3:TSS-DMR**	Yes	0.616324588	0.053168231	0.456819895	0.77582928	0.281311933	-0.309494465	0.660322569	0.069516171	0.655538314	0.064731917
14	101370990	cg06804177	**MEG8:Int2-DMR**	Yes	0.780120575	0.026218371	0.701465463	0.858775687	0.929532271	0.183821753	0.759046855	0.013336336	0.754082837	0.008372319
15	22833150	cg07959104	TUBGCP5	Yes	0.199778105	0.067258345	-0.001996929	0.401553139	0.239982447	0.051921124	0.091029404	-0.097031919	0.179078272	-0.008983051
15	22833173	cg17809365	TUBGCP5	Yes	0.121717586	0.056168419	-0.046787672	0.290222844	0.118925501	0.004306543	0.058540798	-0.056078161	0.113448914	-0.001170045
15	22833214	cg16707215	TUBGCP5	Yes	0.082943062	0.047523089	-0.059626205	0.22551233	0.093121303	0.014922905	0.026284497	-0.0519139	0.082319451	0.004121054
15	23807181	cg17819168	**MKRN3:TSS-DMR**	Yes	0.86639053	0.043241989	0.736664564	0.996116496	0.80906716	-0.018614364	0.905902591	0.078221066	0.88146909	0.053787566
15	23807235	cg13423098	**MKRN3:TSS-DMR**	Yes	0.566960091	0.059965385	0.387063936	0.746856246	0.493558313	-0.048411865	0.598050949	0.05608077	0.58717139	0.045201211
15	23809946	cg07501539	**MKRN3:TSS-DMR**	NA	0.934970872	0.014338734	0.89195467	0.977987075	0.956865401	0.061325102	0.939153517	0.043613217	0.921196883	0.025656584
15	23810164	cg09437135	**MKRN3:TSS-DMR**	Yes	0.92641801	0.018938407	0.869602787	0.983233232	0.914701157	0.027401675	0.90098748	0.013687998	0.952900461	0.065600978
15	23810239	cg20769842	**MKRN3:TSS-DMR**	Yes	0.877448421	0.026807351	0.797026367	0.957870476	0.8551398	0.015133979	0.896694155	0.056688333	0.921222097	0.081216276
15	23810281	cg16131766	**MKRN3:TSS-DMR**	Yes	0.975857809	0.007879405	0.952219593	0.999496024	0.964296795	0.029715526	0.979476124	0.044894855	0.984446449	0.04986518
15	23810335	cg20792895	**MKRN3:TSS-DMR**	Yes	0.978629602	0.008125122	0.954254236	1.003004968	0.969171387	0.031619359	0.981560864	0.044008835	0.985263585	0.047711556
15	23810379	cg05952543	**MKRN3:TSS-DMR**	Yes	0.981649447	0.00506903	0.966442356	0.996856538	0.982967488	0.042362923	0.983258696	0.042654132	0.984910326	0.044305761
15	23810653	cg27046052	**MKRN3:TSS-DMR**	Yes	0.953059001	0.017615835	0.900211494	1.005906507	0.937995666	0.025407406	0.948047959	0.035459699	0.972730712	0.060142452
15	23810665	cg03969797	**MKRN3:TSS-DMR**	NA	0.907725121	0.03562948	0.800836682	1.014613559	0.850611291	-0.017495815	0.892618039	0.024510932	0.95118753	0.083080424
15	23810844	cg00215587	**MKRN3:TSS-DMR**	Yes	0.971718443	0.011991493	0.935743963	1.007692923	0.95035953	0.019586858	0.970198701	0.039426029	0.986633761	0.055861089
15	23810850	cg13096454	**MKRN3:TSS-DMR**	NA	0.980062536	0.007829886	0.956572879	1.003552193	0.965633993	0.026618778	0.985258468	0.046243253	0.987228044	0.048212829
15	23810862	cg11100640	**MKRN3:TSS-DMR**	Yes	0.970733668	0.012450869	0.93338106	1.008086275	0.947521331	0.01754276	0.972237596	0.042259025	0.984493437	0.054514866
15	23811206	cg23234999	**MKRN3:TSS-DMR**	Yes	0.973697466	0.009670277	0.944686634	1.002708298	0.957976389	0.024984247	0.981263672	0.04827153	0.974947231	0.041955089
15	23811567	cg13415551	**MKRN3:TSS-DMR**	NA	0.895877923	0.024887738	0.821214708	0.970541137	0.856094505	-0.001500405	0.908520782	0.050925872	0.885560407	0.027965497
15	23811573	cg20525221	**MKRN3:TSS-DMR**	NA	0.904432517	0.01873344	0.848232198	0.960632836	0.871879006	0.005427666	0.925172192	0.058720851	0.877046368	0.010595028
15	23812335	cg07760796	**MKRN3:TSS-DMR**	Yes	0.945972249	0.017101653	0.894667291	0.997277207	0.912793575	0.00712666	0.962212427	0.056545511	0.963946653	0.058279738
15	23892575	cg20747630	**MAGEL2:TSS-DMR**	Yes	0.602491164	0.028224824	0.51781669	0.687165637	0.553188507	-0.02339469	0.642593119	0.066009922	0.614134453	0.037551256
15	23892657	cg04142555	**MAGEL2:TSS-DMR**	Yes	0.39674778	0.038202677	0.28213975	0.511355811	0.331899517	-0.046135825	0.438073727	0.060038385	0.402079918	0.024044576
15	23892771	cg14432018	**MAGEL2:TSS-DMR**	Yes	0.702088771	0.028526357	0.6165097	0.787667841	0.697221684	0.02543917	0.706924782	0.035142269	0.696151979	0.024369465
15	23893026	cg19961998	**MAGEL2:TSS-DMR**	Yes	0.469641874	0.047258557	0.327866205	0.611417544	0.510805407	0.061573601	0.450971693	0.001739888	0.473431161	0.024199355
15	23893031	cg22925508	**MAGEL2:TSS-DMR**	NA	0.37075809	0.031004978	0.277743156	0.463773024	0.397578267	0.040736143	0.352717715	-0.004124409	0.38208828	0.025246155
15	23893041	cg18253764	**MAGEL2:TSS-DMR**	Yes	0.473761726	0.028753957	0.387499857	0.560023596	0.466706022	0.011926259	0.451905396	-0.002874367	0.501788792	0.047009029
15	23893087	cg19147262	**MAGEL2:TSS-DMR**	NA	0.654815016	0.039559115	0.536137672	0.773492361	0.722210239	0.093620566	0.594406425	-0.034183248	0.657692253	0.02910258
15	23893743	cg22872376	**MAGEL2:TSS-DMR**	Yes	0.468679669	0.067580361	0.265938585	0.671420752	0.387722721	-0.05684278	0.512010271	0.06744477	0.475211641	0.03064614
15	23893770	cg20871407	**MAGEL2:TSS-DMR**	NA	0.73870301	0.038090932	0.624430214	0.852975807	0.727811828	0.022368675	0.727165813	0.02172266	0.760631649	0.055188496
15	23931941	cg24215869	**NDN:TSS-DMR**	NA	0.401993413	0.040052797	0.281835021	0.522151806	0.416102933	0.032303198	0.358004357	-0.025795377	0.486964858	0.103165123
15	23932371	cg12532169	**NDN:TSS-DMR**	Yes	0.462040836	0.050587611	0.310278002	0.613803671	0.457162362	0.016024005	0.420308621	-0.020829736	0.503288574	0.062150216
15	23932374	cg13828758	**NDN:TSS-DMR**	Yes	0.515093684	0.044471162	0.3816802	0.648507169	0.509228185	0.018836666	0.486106671	-0.004284849	0.548855422	0.058463902
15	23932398	cg22694067	**NDN:TSS-DMR**	Yes	0.408675331	0.062862711	0.220087199	0.597263464	0.44897689	0.061970438	0.401345211	0.014338758	0.416249026	0.029242574

15	23932413	cg26725891	**NDN:TSS-DMR**	Yes	0.5632451	0.037914082	0.449502853	0.676987347	0.613306567	0.07423526	0.583197233	0.044125926	0.549449027	0.01037772
15	23932621	cg18552939	**NDN:TSS-DMR**	Yes	0.264655379	0.053819617	0.103196526	0.426114231	0.253922918	0.001609646	0.226630168	-0.025683103	0.316360601	0.064047329
15	23932627	cg19089045	**NDN:TSS-DMR**	NA	0.225970167	0.047008107	0.084945845	0.366994489	0.237512296	0.022452094	0.181183955	-0.033876247	0.23490855	0.019848349
15	23932759	cg01989224	**NDN:TSS-DMR**	Yes	0.664390265	0.054717029	0.500239177	0.828541353	0.704488706	0.072354142	0.692246133	0.060111568	0.607698003	-0.024436561
15	24142921	cg06013632	PWRN4	Yes	0.47973423	0.039658615	0.360758386	0.598710074	0.474718182	0.016542273	0.538249057	0.080073149	0.497614936	0.039439027
15	24142936	cg01250563	PWRN4	Yes	0.468547839	0.042612975	0.340708912	0.596386765	0.497417367	0.049358271	0.472919787	0.024860692	0.446124788	-0.001934308
15	24143001	cg19698403	PWRN4	Yes	0.21557788	0.026579487	0.135839417	0.295316342	0.217344456	0.01155116	0.197694006	-0.00809929	0.229258182	0.023464886
15	25068565	cg22491305	**SNRPN:alt-TSS-DMR**	Yes	0.908793694	0.01538494	0.862638874	0.954948514	0.931580814	0.061816644	0.891443266	0.021679096	0.915679376	0.045915206
15	25068739	cg11265941	**SNRPN:alt-TSS-DMR**	Yes	0.624980497	0.042315496	0.498034009	0.751926986	0.570749079	-0.026529488	0.638838716	0.04156015	0.664124127	0.06684556
15	25068755	cg11826663	**SNRPN:alt-TSS-DMR**	Yes	0.629850657	0.028371785	0.544735304	0.714966011	0.61087785	0.009126814	0.648973469	0.047222433	0.630207557	0.028456521
15	25068758	cg24785225	**SNRPN:alt-TSS-DMR**	Yes	0.708542034	0.028342291	0.62351516	0.793568907	0.682125354	0.00457258	0.743360415	0.065807642	0.715607553	0.038054779
15	25068764	cg10271763	**SNRPN:alt-TSS-DMR**	Yes	0.685742865	0.025806458	0.608323491	0.76316224	0.653819067	-0.002348285	0.701797334	0.045629982	0.701070031	0.044902679
15	25068770	cg27304225	**SNRPN:alt-TSS-DMR**	Yes	0.681674457	0.039019024	0.564617385	0.798731529	0.644754888	-0.007512304	0.713580824	0.061313632	0.710136628	0.057869437
15	25068791	cg19803984	**SNRPN:alt-TSS-DMR**	Yes	0.509711268	0.048808212	0.363286632	0.656135904	0.501426365	0.016064733	0.474697046	-0.010664586	0.538385063	0.053023431
15	25069377	cg26033681	**SNRPN:alt-TSS-DMR**	Yes	0.620696972	0.058713131	0.444557578	0.796836367	0.666451555	0.075495843	0.571723099	-0.019232614	0.596301265	0.005345552
15	25093245	cg25978208	**SNRPN:Int1-DMR1**	Yes	0.576479854	0.060019868	0.39642025	0.756539458	0.513828369	-0.037666122	0.682194987	0.130700495	0.503162247	-0.048332244
15	25093367	cg02171545	**SNRPN:Int1-DMR1**	Yes	0.330237943	0.085289041	0.07437082	0.586105066	0.284133696	-0.028633742	0.283188421	-0.029579017	0.448739643	0.135972205
15	25093457	cg02305723	**SNRPN:Int1-DMR1**	Yes	0.505019909	0.06275192	0.316764149	0.693275669	0.461599911	-0.025513124	0.585320917	0.098207882	0.495697928	0.008584894
15	25093522	cg22678136	**SNRPN:Int1-DMR1**	Yes	0.277352295	0.07135265	0.063294346	0.491410244	0.227764502	-0.038964512	0.355507251	0.088778237	0.234233372	-0.032495642
15	25123288	cg27644292	**SNRPN:nt1-DMR2**	Yes	0.680037576	0.054291854	0.517162015	0.842913137	0.692837826	0.042573871	0.738774612	0.088510657	0.657458232	0.007194277
15	25123382	cg08560373	**SNRPN:nt1-DMR2**	Yes	0.375143916	0.087276323	0.113314948	0.636972885	0.390814185	0.030243562	0.439216028	0.078645406	0.297741671	-0.062828952
15	25123492	cg25657700	**SNRPN:nt1-DMR2**	Yes	0.809952066	0.035267293	0.704150186	0.915753945	0.777149075	0.001802517	0.850895679	0.075549122	0.817613033	0.042266476
15	25123689	cg01786704	**SNRPN:nt1-DMR2**	Yes	0.520619265	0.124390517	0.147447715	0.893790814	0.513424636	0.019318138	0.503555212	0.009448714	0.477862876	-0.016243621
15	25123732	cg21870668	**SNRPN:nt1-DMR2**	Yes	0.561789882	0.063316986	0.371838925	0.751740839	0.527193075	-0.00830436	0.668985451	0.133488016	0.560017334	0.024519899
15	25200134	cg17916021	**SNURF:TSS-DMR**	NA	0.647227168	0.025962339	0.56934015	0.725114186	0.614618572	-0.003932174	0.678652137	0.060101391	0.637805983	0.019255237
15	25200146	cg04072648	**SNURF:TSS-DMR**	NA	0.641291456	0.033231584	0.541596705	0.740986206	0.647286138	0.034108295	0.648442982	0.03526514	0.638800279	0.025622437
15	25200203	cg17503663	**SNURF:TSS-DMR**	NA	0.737996683	0.0282384	0.653281484	0.822711882	0.741543262	0.036162996	0.745630896	0.04025063	0.752191763	0.046811497
15	25200254	cg18506672	**SNURF:TSS-DMR**	Yes	0.613373509	0.043975841	0.481445985	0.745301033	0.571697468	-0.014834321	0.662009195	0.075477405	0.597380012	0.010848222
15	25200407	cg02125271	**SNURF:TSS-DMR**	Yes	0.861319399	0.016811734	0.810884198	0.911754601	0.849451554	0.024474889	0.878361114	0.053384449	0.861493877	0.036517212
15	25201021	cg22159025	**SNURF:TSS-DMR**	Yes	0.544162658	0.045111048	0.408829515	0.679495802	0.604986635	0.084020169	0.535799387	0.014832921	0.516489604	-0.004476862
15	25201225	cg22555495	**SNURF:TSS-DMR**	Yes	0.629732015	0.035448233	0.523387315	0.736076715	0.613997439	0.012597083	0.644079613	0.042679257	0.636311146	0.034910789
15	25201430	cg01614564	**SNURF:TSS-DMR**	Yes	0.621085372	0.039466543	0.502685744	0.739485	0.607340538	0.015391703	0.643270309	0.051321473	0.640728239	0.048779403
15	25201733	cg13073261	**SNURF:TSS-DMR**	Yes	0.512355957	0.03161827	0.417501145	0.607210768	0.515122817	0.025373482	0.54298604	0.053236705	0.505793195	0.01604386
15	25227037	cg05894557	PWAR	Yes	0.935998743	0.017240653	0.884276783	0.987720703	0.927204109	0.030860128	0.95637821	0.06003423	0.941934852	0.045590871
15	25229257	cg26410550	PWAR	Yes	0.957160145	0.014684996	0.913105157	1.001215133	0.971294434	0.054917826	0.942254541	0.025877933	0.969262461	0.052885854
15	25229523	cg02891726	PWAR	Yes	0.968931364	0.007095018	0.94764631	0.990216418	0.971575655	0.043353376	0.975266946	0.047044667	0.962204352	0.033982073
15	25230201	cg18560144	PWAR	Yes	0.981531789	0.004753363	0.967271701	0.995791877	0.988340769	0.04792206	0.978082281	0.037663572	0.979962782	0.039544074
15	25230296	cg18488946	PWAR	Yes	0.833766625	0.04108521	0.710510995	0.957022255	0.825507895	0.027052867	0.850030626	0.051575598	0.852682443	0.054227415
15	25296465	cg13994177	SNORD116	Yes	0.959376402	0.014173994	0.916854421	1.001898382	0.97457533	0.055706695	0.966519494	0.047650859	0.934107426	0.015238791
15	25296572	cg03381111	SNORD116	Yes	0.82830901	0.030149528	0.737860428	0.918757593	0.836889436	0.045019125	0.794829156	0.002958845	0.858187511	0.0663172
15	25296629	cg10832166	SNORD116	Yes	0.970469086	0.007674243	0.947446358	0.993491814	0.963518238	0.033723799	0.976257273	0.046462834	0.975486047	0.045691608
15	25298230	cg22837251	SNORD116	Yes	0.979745479	0.006680801	0.959703075	0.999787884	0.985159898	0.046385262	0.987304134	0.048529497	0.970649336	0.031874699
15	25298517	cg03709297	SNORD116	Yes	0.972179464	0.006495049	0.952694318	0.99166461	0.977356674	0.045923363	0.979853648	0.048420337	0.957032978	0.025599667
15	25298854	cg03432464	SNORD116	Yes	0.921910272	0.019747874	0.862666649	0.981153894	0.931947182	0.049148318	0.927907519	0.045108655	0.904637401	0.021838537
15	25299158	cg03840075	SNORD116	Yes	0.979206434	0.005629498	0.962317942	0.996094927	0.985285924	0.046744135	0.982423615	0.043881827	0.973648819	0.035107031
15	25299173	cg00475883	SNORD116	Yes	0.985491991	0.005177162	0.969960506	1.001023476	0.981841906	0.037313812	0.99130649	0.046778396	0.9859583	0.041430206
15	25299212	cg14898768	SNORD116	Yes	0.908151258	0.021436514	0.843841716	0.972460801	0.921658165	0.052537183	0.895235918	0.026114936	0.900386024	0.031265042
15	25300688	cg16067029	SNORD116	Yes	0.965721794	0.009299467	0.937823392	0.993620195	0.96445441	0.038957743	0.976273251	0.050776584	0.958076855	0.032580188
15	25301784	cg06126413	SNORD116	Yes	0.989275747	0.003603631	0.978464853	1.000086641	0.993495819	0.045520567	0.986845783	0.03887053	0.988756205	0.040780953
15	25301861	cg23740975	SNORD116	Yes	0.890737454	0.028311551	0.805802802	0.975672107	0.892505709	0.03993806	0.914449479	0.06188183	0.835802526	-0.016765123
15	25319114	cg14172427	SNORD116	Yes	0.914742123	0.028039991	0.830622151	0.998862096	0.942854554	0.06603808	0.926077292	0.049260818	0.861899299	-0.014917175
15	25319652	cg14182820	SNORD116	Yes	0.913051228	0.024286512	0.840191692	0.985910765	0.932691851	0.058915385	0.91346661	0.039690145	0.899749839	0.025973373
15	25320168	cg14798020	SNORD116	Yes	0.966742605	0.011795845	0.931355071	1.00213014	0.969518848	0.043319523	0.980379323	0.054179998	0.962883731	0.036684407
15	25320196	cg22132343	SNORD116	Yes	0.97459339	0.014609334	0.930765387	1.018421394	0.986661702	0.052714859	0.983876697	0.049929854	0.958921887	0.024975044
15	25320887	cg04524239	SNORD116	Yes	0.956448343	0.010973672	0.923527326	0.989369359	0.964703185	0.048507006	0.965538194	0.049342014	0.949079281	0.032883101
15	25321222	cg08661338	SNORD116	Yes	0.830548077	0.04523839	0.694832906	0.966263248	0.813676354	0.018416272	0.809666425	0.014406343	0.85287585	0.057615768
15	25321717	cg19708133	SNORD116	Yes	0.894462264	0.020314817	0.833517814	0.955406713	0.915943288	0.059612042	0.861869424	0.005538179	0.89601266	0.039681414
15	25322009	cg17742273	SNORD116	Yes	0.740633156	0.035171166	0.635119657	0.846146654	0.774796304	0.067952576	0.710362167	0.003518439	0.725166805	0.018323077
15	25322129	cg01620672	SNORD116	Yes	0.952605864	0.022986686	0.883645805	1.021565923	0.942737749	0.029948416	0.977168884	0.064379551	0.931826892	0.019037559
15	25322898	cg01699028	SNORD116	Yes	0.720270565	0.040856442	0.597701239	0.842839891	0.680432472	-0.007328454	0.770798326	0.0830374	0.694058392	0.006297466
15	25323794	cg05864627	SNORD116	Yes	0.959931377	0.007984885	0.935976723	0.983886031	0.958483101	0.038827068	0.965862677	0.046206644	0.960422904	0.040766871
15	25323796	cg21844656	SNORD116	Yes	0.961374972	0.016114759	0.913030694	1.009719249	0.960176635	0.039015599	0.972561854	0.051400819	0.967759556	0.04659852
15	25324125	cg08170950	SNORD116	Yes	0.983341388	0.004637716	0.96942824	0.997254535	0.987064888	0.04458365	0.987892898	0.04541166	0.984660041	0.042178803
15	25325714	cg09838956	SNORD116	Yes	0.943783084	0.018243369	0.889052976	0.998513192	0.920260072	0.015676299	0.94465395	0.040070177	0.956889447	0.052305673
15	25325902	cg19183867	SNORD116	NA	0.768478569	0.030009849	0.678449023	0.858508116	0.729018329	-0.007683238	0.775314762	0.038613195	0.793878001	0.057176435
15	25326264	cg20385284	SNORD116	Yes	0.902631891	0.021741402	0.837407686	0.967856096	0.919285009	0.054222563	0.882821065	0.017758618	0.896978254	0.031915808
15	25326511	cg19844955	SNORD116	Yes	0.969704812	0.007429688	0.947415746	0.991993877	0.966989908	0.037961689	0.975019384	0.045991165	0.96521147	0.03618325
15	25327490	cg05172655	SNORD116	Yes	0.920532419	0.017667679	0.867529383	0.973535454	0.895716467	0.013453406	0.938878475	0.056615414	0.911384687	0.029121626
15	25327920	cg07897695	SNORD116	Yes	0.942106233	0.016216298	0.89345734	0.990755126	0.954222306	0.050956767	0.92639479	0.023129251	0.941967797	0.038702258
15	25328740	cg05951178	SNORD116	Yes	0.967279757	0.008816394	0.940830576	0.993728938	0.950516559	0.023977483	0.974420122	0.047881045	0.976794609	0.050255533
15	25329169	cg26280499	SNORD116	Yes	0.614341738	0.043300895	0.484439054	0.744244422	0.648697087	0.061968008	0.587518717	0.000789637	0.576893305	-0.009835774
15	25329355	cg03760060	SNORD116	Yes	0.860059027	0.042481046	0.732615887	0.987502166	0.872956531	0.050183709	0.851990391	0.029217569	0.85275207	0.029979248
15	25329448	cg02625244	SNORD116	Yes	0.629592854	0.039977327	0.509660871	0.749524836	0.661491636	0.060216511	0.633482357	0.032207232	0.566212837	-0.035062288
15	25330243	cg16978004	SNORD116	Yes	0.795163609	0.038136414	0.680754366	0.909572852	0.788997745	0.02737979	0.761594372	-2.35836E-05	0.827081764	0.065463809
15	25330342	cg08051964	SNORD116	Yes	0.800219291	0.033645357	0.699283219	0.901155363	0.845377021	0.080060106	0.795734915	0.030418	0.787857824	0.022540909
15	25330371	cg13075322	SNORD116	Yes	0.780047969	0.031313159	0.68610849	0.873987447	0.789468323	0.042445915	0.737428364	-0.009594043	0.768045356	0.021022949
15	25330537	cg01871408	SNORD116	Yes	0.970877278	0.008431041	0.945584155	0.996170401	0.981880359	0.052114998	0.968914482	0.039149121	0.961276346	0.031510985
15	25331392	cg00476675	SNORD116	Yes	0.990969696	0.001754111	0.985707364	0.996232029	0.992215042	0.042544194	0.994436284	0.044765436	0.991186895	0.041516047
15	25334118	cg14481604	SNORD116	Yes	0.97314721	0.012932117	0.934350858	1.011943561	0.984197985	0.052021011	0.959703971	0.027526997	0.976157322	0.043980348
15	25334880	cg05585263	SNORD116	Yes	0.910146489	0.051033958	0.757044617	1.063248362	0.90994988	0.039100357	0.952740059	0.081890536	0.897742773	0.02689325
15	25334989	cg16150571	SNORD116	Yes	0.985935539	0.00318784	0.976372017	0.99549906	0.985645604	0.040711262	0.990677704	0.045743362	0.985045729	0.040111387
15	25335705	cg20025389	SNORD116	Yes	0.970775314	0.009817339	0.941323298	1.00022733	0.976980914	0.046663978	0.96575932	0.035442384	0.979261039	0.048944103
15	25335746	cg17077566	SNORD116	Yes	0.953556571	0.021028775	0.890470246	1.016642896	0.930972015	0.016891817	0.943947503	0.029867305	0.974971739	0.060891541
15	25337004	cg20066737	SNORD116	Yes	0.926517385	0.023271722	0.856702218	0.996332552	0.948914589	0.061895627	0.915120569	0.028101607	0.923565888	0.036546926
15	25337645	cg03799387	SNORD116	Yes	0.988007755	0.003148495	0.97856227	0.997453241	0.989163976	0.042552903	0.989007925	0.042396853	0.986716667	0.040105595
15	25338220	cg15590133	SNORD116	Yes	0.572984146	0.05405342	0.410823885	0.735144406	0.609992583	0.062006915	0.536549327	-0.011436341	0.538405576	-0.009580091
15	25426393	cg07050869	SNORD115	Yes	0.800255307	0.050600903	0.648452597	0.952058017	0.733122384	-0.02938795	0.75821432	-0.004296015	0.870235666	0.107725332
15	25427059	cg25230549	SNORD115	Yes	0.888534418	0.032011602	0.792499611	0.984569225	0.921186474	0.071952889	0.846679575	-0.00255401	0.912124195	0.062890611
15	25427510	cg08343671	SNORD115	Yes	0.824126955	0.039533925	0.705525179	0.94272873	0.81538747	0.026486238	0.83875812	0.049856887	0.869463909	0.080562677
15	25427937	cg01258039	SNORD115	Yes	0.988326332	0.002945884	0.97948868	0.997163985	0.988510692	0.041486437	0.987981575	0.04095732	0.990348022	0.043323767
15	25429177	cg00284551	SNORD115	Yes	0.777961335	0.045408228	0.641736652	0.914186019	0.789438966	0.046485857	0.739151701	-0.003801408	0.807626598	0.064673489
15	45314790	cg06424894	SORD	Yes	0.423209983	0.080234428	0.182506698	0.663913267	0.35537621	-0.050151145	0.476873634	0.071346279	0.467945343	0.062417988
15	45314916	cg22023531	SORD	Yes	0.511843981	0.094851119	0.227290624	0.796397338	0.423453048	-0.070547895	0.587958732	0.09395779	0.596090596	0.102089653
15	45314934	cg27073142	SORD	Yes	0.214212148	0.060435926	0.032904371	0.395519924	0.164317128	-0.044772187	0.275133602	0.066044287	0.251408571	0.042319256
15	45314944	cg08414676	SORD	Yes	0.514643727	0.033791467	0.413269326	0.616018128	0.477535157	-0.016630804	0.555628266	0.061462305	0.530767659	0.036601697
15	99408637	cg19322380	**IGF1R:Int2-DMR**	Yes	0.811617375	0.055758137	0.644342963	0.978891786	0.807422964	0.033962901	0.777482262	0.004022199	0.910250109	0.136790047
15	99408805	cg21746425	**IGF1R:Int2-DMR**	Yes	0.575312205	0.067172093	0.373795925	0.776828485	0.540942478	-0.004300704	0.512648642	-0.03259454	0.668555498	0.123312316
15	99408959	cg12553689	**IGF1R:Int2-DMR**	Yes	0.724883804	0.062626244	0.537005071	0.912762536	0.704987654	0.014902731	0.669852494	-0.020232429	0.820676179	0.130591257
15	99409195	cg07615383	**IGF1R:Int2-DMR**	Yes	0.465257562	0.055556479	0.298588125	0.631926999	0.403709594	-0.039305648	0.455906275	0.012891033	0.563356025	0.120340783
15	99409361	cg00098799	**IGF1R:Int2-DMR**	NA	0.642984847	0.063785865	0.451627252	0.834342443	0.580469626	-0.030576272	0.606125342	-0.004920555	0.749492107	0.138446209
15	99409412	cg13812291	**IGF1R:Int2-DMR**	Yes	0.736573178	0.059497399	0.55808098	0.915065376	0.656047787	-0.044961695	0.749421544	0.048412062	0.815208146	0.114198664
15	99409507	cg03380198	**IGF1R:Int2-DMR**	Yes	0.611534769	0.126619121	0.231677405	0.991392133	0.471053832	-0.109244695	0.62942848	0.049129953	0.728464776	0.148166248
16	3345464	cg07116631	TIGD7	Yes	0.678448873	0.053022461	0.519381492	0.837516255	0.706279362	0.054958671	0.753143692	0.101823001	0.665322963	0.014002272
16	3345605	cg08555866	TIGD7	Yes	0.866072394	0.033065795	0.76687501	0.965269779	0.85826838	0.028918472	0.899802961	0.070453053	0.895712984	0.066363076
16	3414984	cg27295850	MTRNR2L4	Yes	0.349869157	0.060749884	0.167619505	0.53211881	0.324433395	-0.01227463	0.445235264	0.108527239	0.343130208	0.006422183
16	3415257	cg02898051	MTRNR2L4	Yes	0.101554465	0.039860173	-0.018026052	0.221134983	0.095528733	-0.002570071	0.125117532	0.027018728	0.092892374	-0.00520643
16	3415312	cg07388216	MTRNR2L4	Yes	0.102013363	0.041548556	-0.022632304	0.226659029	0.06593744	-0.031459026	0.09980435	0.002407884	0.144995563	0.047599097
16	3481971	cg02880119	**ZNF597:3'-DMR**	Yes	0.721515203	0.064553398	0.527855008	0.915175397	0.70981663	0.019426293	0.728645712	0.038255375	0.766061234	0.075670897
16	3482079	cg04027059	**ZNF597:3'-DMR**	Yes	0.774669408	0.051182241	0.621122684	0.928216131	0.677262134	-0.064028175	0.853435421	0.112145112	0.804555849	0.06326554
16	3482233	cg12472342	**ZNF597:3'-DMR**	NA	0.800869362	0.047720173	0.657708843	0.944029881	0.757536428	-0.008290699	0.884269652	0.118442524	0.821930434	0.056103306
16	3493134	cg06410284	**ZNF597:TSS-DMR**	Yes	0.534820174	0.055609922	0.367990408	0.70164994	0.542623484	0.032617137	0.541646334	0.031639988	0.50546137	-0.004544977
16	3493337	cg09596866	**ZNF597:TSS-DMR**	Yes	0.448454775	0.05154846	0.293809396	0.603100155	0.453848948	0.022126998	0.504186163	0.072464212	0.40675831	-0.02496364
16	3493424	cg10482201	**ZNF597:TSS-DMR**	Yes	0.629881671	0.040010444	0.509850339	0.749913004	0.65461467	0.049590733	0.631532436	0.0265085	0.638476832	0.033452895
16	3493429	cg24333473	**ZNF597:TSS-DMR**	Yes	0.496853849	0.061577618	0.312120995	0.681586703	0.487993931	0.014094278	0.50593937	0.032039717	0.480931399	0.007031745
16	3493441	cg03896694	**ZNF597:TSS-DMR**	Yes	0.599998571	0.06636531	0.400902642	0.7990945	0.584091552	0.0073827	0.644875728	0.068166875	0.599447882	0.02273903
16	3493535	cg27136643	**ZNF597:TSS-DMR**	Yes	0.689872731	0.047083666	0.548621732	0.83112373	0.664359953	0.004965964	0.708056442	0.048662453	0.705107499	0.04571351

16	3493615	cg03933495	**ZNF597:TSS-DMR**	Yes	0.419640767	0.076639347	0.189722726	0.649558808	0.374980848	-0.024033787	0.427511861	0.028497226	0.449006614	0.049991979
16	3493642	cg24027317	**ZNF597:TSS-DMR**	NA	0.571474455	0.057215837	0.399826943	0.743121967	0.575848016	0.031530925	0.576286767	0.031969676	0.531565389	-0.012751702
16	3493682	cg27460547	**ZNF597:TSS-DMR**	Yes	0.524798627	0.055556111	0.358130294	0.691466959	0.531977311	0.031474827	0.501360024	0.000857539	0.520303684	0.019801199
16	3493688	cg01344564	**ZNF597:TSS-DMR**	NA	0.548289621	0.048430826	0.402997144	0.693582098	0.532513308	0.008377135	0.534338688	0.010202514	0.546203558	0.022067385
16	3493816	cg26918062	**ZNF597:TSS-DMR**	Yes	0.509021228	0.057892526	0.33534365	0.682698806	0.488672881	0.003895396	0.514590664	0.029813179	0.528041133	0.043263648
16	3494007	cg14372630	**ZNF597:TSS-DMR**	NA	0.433209964	0.046741852	0.292984408	0.57343552	0.365897392	-0.045632625	0.447498339	0.035968323	0.479665966	0.06813595
16	3494095	cg10406676	**ZNF597:TSS-DMR**	Yes	0.585070615	0.058286258	0.41021184	0.75992939	0.591555277	0.033707646	0.572847748	0.015000117	0.604063444	0.046215813
16	3494156	cg06121207	**ZNF597:TSS-DMR**	Yes	0.582668351	0.057609627	0.409839471	0.755497231	0.550647537	-0.00801546	0.583223594	0.024560597	0.636676161	0.078013163
19	54040775	cg20646939	**ZNF331:alt-TSS-DMR1**	Yes	0.525767886	0.054211572	0.363133168	0.688402603	0.504525743	0.005319646	0.516964988	0.01775889	0.547218357	0.04801226
19	54040814	cg06855497	**ZNF331:alt-TSS-DMR1**	Yes	0.573568947	0.047078248	0.432334205	0.71480369	0.549519903	0.002793249	0.591798223	0.045071569	0.564391729	0.017665075
19	54040819	cg15576576	**ZNF331:alt-TSS-DMR1**	Yes	0.592122854	0.034260092	0.489342577	0.694903131	0.569474731	0.001290381	0.605531161	0.037346811	0.585896728	0.017712378
19	54040837	cg16020674	**ZNF331:alt-TSS-DMR1**	NA	0.508979857	0.088414318	0.243736904	0.774222809	0.444281961	-0.038679964	0.510008454	0.027046529	0.542817591	0.059855666
19	54041164	cg22475353	**ZNF331:alt-TSS-DMR1**	Yes	0.338892829	0.031110007	0.245562809	0.43222285	0.329621416	0.005307388	0.326223187	0.001909159	0.370109559	0.045795531
19	54041252	cg27296330	**ZNF331:alt-TSS-DMR1**	Yes	0.318810596	0.044278111	0.185976263	0.45164493	0.321639701	0.018793644	0.320425787	0.01757973	0.311196142	0.008350085
19	54041304	cg10023249	**ZNF331:alt-TSS-DMR1**	Yes	0.200711896	0.036733398	0.090511701	0.31091209	0.180984794	-0.013179445	0.21720697	0.023042732	0.201437372	0.007273134
19	54041309	cg15607708	**ZNF331:alt-TSS-DMR1**	Yes	0.299751289	0.035158568	0.194275584	0.405226994	0.279167874	-0.009344289	0.293849405	0.005337243	0.334430175	0.045918012
19	54041399	cg05338009	**ZNF331:alt-TSS-DMR1**	Yes	0.448773885	0.086975112	0.18784855	0.709699221	0.455498042	0.026902489	0.449178775	0.020673222	0.46145604	0.032410487
19	54041520	cg03643149	**ZNF331:alt-TSS-DMR1**	NA	0.448898986	0.034677392	0.344866809	0.552931162	0.43201648	0.001799299	0.472426502	0.042209321	0.456163984	0.025946803
19	54041848	cg14593306	**ZNF331:alt-TSS-DMR1**	NA	0.483750558	0.055163132	0.318261162	0.649239953	0.478501566	0.018051505	0.508827671	0.04837761	0.478243478	0.017793417
19	54041857	cg03499639	**ZNF331:alt-TSS-DMR1**	Yes	0.547489682	0.059523164	0.368920189	0.726059175	0.516998151	-0.005183716	0.58701862	0.064836383	0.552945757	0.03076352
19	54057209	cg06860848	**ZNF331:alt-TSS-DMR2**	Yes	0.574460786	0.052548867	0.416814186	0.732107386	0.57966416	0.033597494	0.60013781	0.054071145	0.547409021	0.001342356
19	54057416	cg03113572	**ZNF331:alt-TSS-DMR2**	Yes	0.653742512	0.065328159	0.457758034	0.84972699	0.606898039	-0.015819786	0.682105161	0.059387337	0.669574731	0.046856906
19	54057616	cg17223707	**ZNF331:alt-TSS-DMR2**	NA	0.599833416	0.055156206	0.434364797	0.765302034	0.590204063	0.018837837	0.615325832	0.043959607	0.61014648	0.038780255
19	54057672	cg10823956	**ZNF331:alt-TSS-DMR2**	NA	0.536456035	0.039078944	0.419219202	0.653692867	0.510694148	-0.00309791	0.534630002	0.020837944	0.538151373	0.024359315
19	54057706	cg19696891	**ZNF331:alt-TSS-DMR2**	Yes	0.385877182	0.034249504	0.283128669	0.488625695	0.373222909	0.006457036	0.377952988	0.011187115	0.410953837	0.044187964
19	54057795	cg03490784	**ZNF331:alt-TSS-DMR2**	Yes	0.531243032	0.080078019	0.291008975	0.771477089	0.545789194	0.039159857	0.52591938	0.019290043	0.514061043	0.007431707
19	54057813	cg22815181	**ZNF331:alt-TSS-DMR2**	NA	0.686406504	0.080146862	0.445965917	0.926847091	0.616124485	-0.041333445	0.711821391	0.054363461	0.712905503	0.055447573
19	54057900	cg21307424	**ZNF331:alt-TSS-DMR2**	NA	0.564886961	0.032599949	0.467087113	0.662686809	0.566734331	0.025172195	0.581215004	0.039652868	0.542513529	0.000951393
19	54057917	cg12551529	**ZNF331:alt-TSS-DMR2**	NA	0.524911342	0.036155135	0.416445937	0.633376747	0.48473309	-0.014399754	0.54184436	0.042711515	0.554471649	0.055338804
19	54058086	cg20071427	**ZNF331:alt-TSS-DMR2**	Yes	0.633995149	0.057905367	0.460279048	0.807711251	0.623208251	0.020216568	0.655578568	0.052586885	0.629518987	0.026527304
19	57348530	cg14904295	**PEG3:TSS-DMR**	NA	0.391372809	0.071214395	0.177729623	0.605015995	0.330312817	-0.038953381	0.333458657	-0.035807542	0.473156057	0.103889859
19	57349205	cg15473155	**PEG3:TSS-DMR**	Yes	0.571026301	0.026902527	0.49031872	0.651733881	0.573869762	0.025953524	0.618764923	0.070848685	0.536798853	-0.011117384
19	57349680	cg19041006	**PEG3:TSS-DMR**	Yes	0.611109613	0.036761275	0.500825786	0.721393439	0.569469937	-0.014856014	0.645056882	0.060730931	0.602102225	0.017776275
19	57349710	cg17663463	**PEG3:TSS-DMR**	Yes	0.736398626	0.035446882	0.630057981	0.842739272	0.666958243	-0.036179176	0.773129458	0.069992039	0.770521179	0.06738376
19	57349816	cg19771589	**PEG3:TSS-DMR**	Yes	0.653393107	0.034374963	0.550268219	0.756517995	0.62004533	-0.004072748	0.680813767	0.056695688	0.646006042	0.021887964
19	57350005	cg02793099	**PEG3:TSS-DMR**	Yes	0.590415441	0.022221095	0.523752155	0.657078727	0.585932354	0.020271487	0.626929306	0.061268439	0.571154173	0.005493306
19	57350097	cg01656470	**PEG3:TSS-DMR**	Yes	0.72816439	0.02499934	0.653166372	0.803162409	0.697169878	0.00015387	0.743461207	0.0464452	0.736020139	0.039004132
19	57350293	cg27519373	**PEG3:TSS-DMR**	Yes	0.781373256	0.033402466	0.681165859	0.881580653	0.831025827	0.084968705	0.751978023	0.005920901	0.787129206	0.041072084
19	57350314	cg18516833	**PEG3:TSS-DMR**	NA	0.856139905	0.026029213	0.778052265	0.934227545	0.875974203	0.056145234	0.819052309	-0.000776659	0.867943131	0.048114162
19	57350504	cg07310951	**PEG3:TSS-DMR**	Yes	0.570518396	0.046393975	0.431336472	0.709700321	0.599198896	0.056360225	0.515052548	-0.027786123	0.590123307	0.047284636
19	57351214	cg20897667	**PEG3:TSS-DMR**	Yes	0.669328709	0.034014213	0.56728607	0.771371349	0.678233834	0.038745709	0.668352729	0.028864604	0.673442131	0.033954007
19	57351323	cg02478023	**PEG3:TSS-DMR**	Yes	0.627955552	0.028614586	0.542111793	0.71379931	0.633399033	0.032809678	0.593166065	-0.00742329	0.66501571	0.064426354
19	57351441	cg22354595	**PEG3:TSS-DMR**	Yes	0.650629029	0.033293808	0.550747606	0.750510453	0.623153539	0.001658386	0.666808806	0.045313653	0.675623464	0.05412831
19	57351643	cg19335327	**PEG3:TSS-DMR**	Yes	0.627204462	0.044572035	0.493488357	0.760920566	0.612805556	0.014813479	0.607558076	0.009566	0.648095501	0.050103425
19	57351792	cg14849423	**PEG3:TSS-DMR**	Yes	0.698876939	0.032510813	0.601344499	0.796409378	0.677283558	0.009983057	0.677622745	0.010322244	0.746416674	0.079116173
19	57352015	cg15777825	**PEG3:TSS-DMR**	Yes	0.536651949	0.018991106	0.479678631	0.593625267	0.530844977	0.017023821	0.526433305	0.012612148	0.546191905	0.032370749
19	57352022	cg12205903	**PEG3:TSS-DMR**	Yes	0.556264496	0.027080052	0.475024339	0.637504652	0.557276867	0.025354745	0.557376236	0.025454115	0.566175431	0.034253309
19	57352075	cg26349266	**PEG3:TSS-DMR**	Yes	0.66349256	0.018400486	0.608291103	0.718694017	0.655431028	0.020884326	0.666434514	0.031887812	0.685619902	0.0510732
19	57352135	cg02162069	**PEG3:TSS-DMR**	Yes	0.583628216	0.03299302	0.484649156	0.682607276	0.561530038	0.003778125	0.575476092	0.017724179	0.605411783	0.04765987
19	57352143	cg13960339	**PEG3:TSS-DMR**	Yes	0.656356466	0.039473198	0.537963873	0.774776059	0.673043025	0.0474988	0.601675472	-0.023868752	0.685452354	0.05990813
19	57352177	cg13369939	**PEG3:TSS-DMR**	Yes	0.673157275	0.025197175	0.597565751	0.7487488	0.659452123	0.015138568	0.679748202	0.035434647	0.693381185	0.04906763
19	57352186	cg20628335	**PEG3:TSS-DMR**	Yes	0.612114968	0.035804919	0.50470021	0.719529726	0.597314698	0.012075071	0.598175519	0.012935892	0.637314839	0.052075213
19	57352253	cg22294267	**PEG3:TSS-DMR**	Yes	0.745437377	0.015454901	0.699072675	0.791802079	0.735999011	0.022972055	0.760621033	0.047594077	0.736400887	0.023373931
19	57352270	cg19098268	**PEG3:TSS-DMR**	Yes	0.63988415	0.038018171	0.525829637	0.753938663	0.649674333	0.039806512	0.649097662	0.03922984	0.611577864	0.001710043
19	57352466	cg24844423	**PEG3:TSS-DMR**	Yes	0.736078401	0.035984722	0.628124234	0.844032567	0.699700979	-0.002507552	0.731439866	0.029231336	0.789524991	0.08731646
19	57352520	cg15678121	**PEG3:TSS-DMR**	Yes	0.734251487	0.038369002	0.619144481	0.849358492	0.747558456	0.047544659	0.773855449	0.073841652	0.689527964	-0.010485833
19	57352543	cg12032885	**PEG3:TSS-DMR**	Yes	0.455328734	0.029098722	0.368032568	0.542624901	0.450929819	0.015150118	0.442971251	0.00719155	0.486260821	0.05048112
19	57352585	cg25458871	**PEG3:TSS-DMR**	Yes	0.616144617	0.050433819	0.464843158	0.767446075	0.63575669	0.050390769	0.577421748	-0.007944173	0.612462613	0.027096692
19	57352658	cg18668753	**PEG3:TSS-DMR**	Yes	0.749965051	0.030914233	0.657222352	0.84270775	0.740814031	0.024787884	0.774301628	0.058275482	0.78016784	0.064141693
19	57352687	cg18706888	**PEG3:TSS-DMR**	Yes	0.664128216	0.041009364	0.541100123	0.787156309	0.671953578	0.03871902	0.650917498	0.01768294	0.680112987	0.046878429
19	57352730	cg01054891	**PEG3:TSS-DMR**	Yes	0.518892694	0.041007632	0.395869798	0.64191559	0.44002596	-0.054476983	0.471934328	-0.022568615	0.555148809	0.060645866
19	57352747	cg18644235	**PEG3:TSS-DMR**	Yes	0.828448687	0.031353428	0.734388405	0.92250897	0.786407103	-0.006365666	0.854940837	0.062168067	0.854898082	0.062125312
19	57352786	cg13374648	**PEG3:TSS-DMR**	Yes	0.587783976	0.034112146	0.485447539	0.690120413	0.58573216	0.025006462	0.561274598	0.000548901	0.601834775	0.041109078
19	57352808	cg26917367	**PEG3:TSS-DMR**	Yes	0.822508803	0.026437123	0.743197485	0.901820222	0.79274213	0.006105545	0.855758599	0.069122014	0.841872821	0.055236236
19	57353129	cg22927979	**PEG3:TSS-DMR**	Yes	0.880268278	0.028704443	0.79415495	0.966381606	0.883916142	0.042041364	0.890117317	0.04824254	0.908424211	0.066549433
20	30134930	cg25359645	**MCT2P:TSS-DMR**	Yes	0.503350216	0.02999422	0.413367556	0.593332876	0.497740106	0.015590683	0.51487679	0.032727367	0.515954421	0.033804998
20	30134974	cg24607140	**MCT2P:TSS-DMR**	Yes	0.713768199	0.027889535	0.630099594	0.797436803	0.690275274	0.008731936	0.755955663	0.074412325	0.712362595	0.030819257
20	30135145	cg15815607	**MCT2P:TSS-DMR**	Yes	0.663515275	0.021638479	0.598599836	0.728430713	0.66477663	0.027844821	0.67004505	0.033108641	0.675385004	0.038453195
20	30135150	cg25645178	**MCT2P:TSS-DMR**	Yes	0.590874254	0.027325686	0.508897195	0.672851313	0.604843596	0.038526771	0.61592833	0.049611505	0.561973542	-0.004343283
20	30135159	cg17840843	**MCT2P:TSS-DMR**	Yes	0.480353845	0.024708658	0.406227871	0.554479818	0.492536067	0.032704095	0.500549873	0.040717901	0.467586887	0.007754915
20	30135178	cg02146091	**MCT2P:TSS-DMR**	Yes	0.571082468	0.026357582	0.492009721	0.650155216	0.606855404	0.06128795	0.58661553	0.041048076	0.548979169	0.003411715
20	30135292	cg14175568	**MCT2P:TSS-DMR**	Yes	0.546770488	0.039600906	0.42796777	0.665573206	0.508292407	-0.013959167	0.554429392	0.032178131	0.563322363	0.041071102
20	30135363	cg20129782	**MCT2P:TSS-DMR**	Yes	0.580793585	0.030709228	0.416665901	0.600921269	0.502538378	0.016941706	0.552496351	0.066899678	0.485888073	0.000291401
20	36148605	cg23757721	**NNAT:TSS-DMR**	Yes	0.841845914	0.037546229	0.729207228	0.9544846	0.807345154	0.001623215	0.883724621	0.078002681	0.836947976	0.031226036
20	36148616	cg26083330	**NNAT:TSS-DMR**	Yes	0.833981926	0.019910525	0.774250352	0.893713501	0.815429121	0.016955117	0.860949883	0.062475878	0.82329851	0.024824506
20	36148621	cg22551578	**NNAT:TSS-DMR**	Yes	0.690481659	0.041483341	0.566031636	0.814931683	0.651267354	-0.010025282	0.734196335	0.072903699	0.688714987	0.02742235
20	36148643	cg04489586	**NNAT:TSS-DMR**	Yes	0.714083714	0.023533537	0.643483104	0.784684325	0.690515993	0.007373468	0.732264412	0.049121887	0.707279939	0.024137415
20	36148673	cg23605670	**NNAT:TSS-DMR**	Yes	0.620303292	0.026217959	0.541649414	0.69895717	0.592755529	-0.001981446	0.653073983	0.058337009	0.621654298	0.026917324
20	36148680	cg12862537	**NNAT:TSS-DMR**	Yes	0.711803651	0.038554325	0.596140677	0.827466626	0.661754855	-0.017646226	0.745817936	0.066416855	0.715862157	0.036461077
20	36148700	cg10981598	**NNAT:TSS-DMR**	Yes	0.644624935	0.031719916	0.549465188	0.739784682	0.609768691	-0.005836225	0.681264303	0.065659387	0.65358425	0.037979334
20	36148739	cg13790727	**NNAT:TSS-DMR**	Yes	0.858231884	0.021102262	0.794925097	0.921538672	0.842385903	0.021486439	0.858248548	0.037349084	0.869426157	0.048526693
20	36148768	cg20783699	**NNAT:TSS-DMR**	Yes	0.784270023	0.027136658	0.702860051	0.865679996	0.755040091	0.005058208	0.817565796	0.067583912	0.791162728	0.041180845
20	36148776	cg25712981	**NNAT:TSS-DMR**	Yes	0.854660049	0.021769901	0.789350345	0.919969753	0.831202134	0.013460213	0.886013515	0.068271593	0.849334768	0.031592847
20	36148780	cg03615235	**NNAT:TSS-DMR**	Yes	0.840245057	0.022731968	0.772049152	0.908440962	0.800407812	-0.003942708	0.876105156	0.071754636	0.845849605	0.041499085
20	36148792	cg17643025	**NNAT:TSS-DMR**	Yes	0.896025395	0.021486766	0.831565095	0.960485694	0.875444689	0.018180836	0.92623982	0.068975967	0.913596618	0.056332765
20	36148799	cg24762053	**NNAT:TSS-DMR**	Yes	0.843090826	0.026115162	0.764745338	0.921436313	0.814318854	0.00852328	0.88634617	0.080550596	0.847817092	0.042021518
20	36148804	cg14469070	**NNAT:TSS-DMR**	Yes	0.892625844	0.021087258	0.82936407	0.955887618	0.854765179	0.000242738	0.916869236	0.062346796	0.90588485	0.05136241
20	36148861	cg22298088	**NNAT:TSS-DMR**	Yes	0.854643681	0.028822906	0.768174963	0.941112399	0.821843738	0.004942346	0.895620552	0.07871916	0.859885946	0.042984555
20	36148929	cg22943498	**NNAT:TSS-DMR**	Yes	0.822073223	0.031597732	0.727280026	0.916866419	0.777768485	-0.008263403	0.862207754	0.076175866	0.840696191	0.054664303
20	36148955	cg14765818	**NNAT:TSS-DMR**	Yes	0.725019603	0.022518274	0.657464781	0.792574424	0.68373305	-0.009966625	0.751045119	0.057345443	0.729969387	0.036269711
20	36148995	cg15473473	**NNAT:TSS-DMR**	Yes	0.793498619	0.038799763	0.677099331	0.909897906	0.737363653	-0.021740278	0.836454882	0.07735095	0.815700433	0.056596501
20	36149014	cg07156273	**NNAT:TSS-DMR**	Yes	0.821448499	0.036101898	0.713142805	0.929754194	0.768055701	-0.018226995	0.866972972	0.080690276	0.833316083	0.047033387
20	36149023	cg01466133	**NNAT:TSS-DMR**	Yes	0.84377602	0.030761922	0.751490254	0.936061785	0.806793602	-0.000275955	0.877344446	0.070274889	0.861080706	0.054011149
20	36149082	cg24675557	**NNAT:TSS-DMR**	Yes	0.77981977	0.029556982	0.691148823	0.868490717	0.758790206	0.012784937	0.823516428	0.077511159	0.767500759	0.021495491
20	36149113	cg20479660	**NNAT:TSS-DMR**	Yes	0.777106292	0.036407521	0.667883729	0.886328856	0.721434982	-0.020826896	0.807491873	0.065229995	0.809799935	0.067538057
20	36149120	cg22421148	**NNAT:TSS-DMR**	Yes	0.814350871	0.032093811	0.718069439	0.910632302	0.776517757	-0.001357343	0.842546887	0.064671787	0.825925791	0.04805069
20	36149122	cg22510412	**NNAT:TSS-DMR**	Yes	0.860320733	0.025106099	0.785002436	0.935639029	0.831706143	0.007545647	0.888175308	0.064014812	0.858574819	0.034414323
20	36149186	cg03061677	**NNAT:TSS-DMR**	Yes	0.813589449	0.027563673	0.730898429	0.896280469	0.765315557	-0.012651556	0.834980117	0.057013004	0.823590312	0.045623199
20	36149189	cg00576435	**NNAT:TSS-DMR**	Yes	0.772416004	0.03612682	0.664035545	0.880796464	0.732262862	-0.005682383	0.802801705	0.06485646	0.781566175	0.04362093
20	36149195	cg18433380	**NNAT:TSS-DMR**	Yes	0.786427424	0.035735859	0.679219845	0.893635002	0.715804681	-0.03467515	0.808764206	0.058284375	0.824727469	0.074247638
20	36149232	cg16648571	**NNAT:TSS-DMR**	Yes	0.640972881	0.02876568	0.554675841	0.727269921	0.584746538	-0.027290786	0.641720581	0.029683257	0.673845443	0.061808119
20	36149272	cg04810287	**NNAT:TSS-DMR**	Yes	0.800459882	0.035546949	0.693819035	0.907100729	0.750723412	-0.014079622	0.79467744	0.029874405	0.840452728	0.075649694
20	36149453	cg25962605	**NNAT:TSS-DMR**	Yes	0.692822888	0.043923639	0.561051972	0.824593804	0.64555026	-0.015778442	0.738022475	0.076693773	0.706757443	0.045428741
20	36149456	cg21588305	**NNAT:TSS-DMR**	Yes	0.827572092	0.030305647	0.73665515	0.918489035	0.787058683	-0.004157876	0.852774263	0.061530703	0.839709695	0.048466136
20	36149657	cg13708635	**NNAT:TSS-DMR**	Yes	0.608567403	0.030462879	0.517178766	0.699956039	0.579135916	-0.002485453	0.61956542	0.037944052	0.62504309	0.043421721
20	36149707	cg11174847	**NNAT:TSS-DMR**	Yes	0.663856283	0.026118577	0.585500551	0.742212015	0.64219767	0.006738835	0.678853446	0.043394612	0.665355533	0.029896698
20	36149751	cg23566503	**NNAT:TSS-DMR**	Yes	0.563146687	0.031731574	0.467951965	0.658341409	0.543135502	0.005697958	0.567585866	0.030148322	0.573967768	0.036530224
20	36150062	cg10642330	**NNAT:TSS-DMR**	Yes	0.48205106	0.032899671	0.383352047	0.580750073	0.446978534	-0.013950504	0.52600191	0.065072872	0.510512114	0.049583077
20	36150136	cg17705814	**NNAT:TSS-DMR**	NA	0.726454927	0.041005261	0.603439145	0.84947071	0.790118761	0.094357486	0.663900126	-0.031861149	0.75337914	0.057617865
20	42142418	cg22457903	**L3MBTL1:alt-TSS-DMR**	Yes	0.559261842	0.052068853	0.403055284	0.7154684	0.576484949	0.044368228	0.557969683	0.025852962	0.523910066	-0.008206655
20	42142452	cg20091959	**L3MBTL1:alt-TSS-DMR**	Yes	0.614212446	0.050590937	0.462439636	0.765985256	0.592133201	0.006532994	0.648058785	0.062458578	0.605123875	0.019523668
20	42142485	cg17091610	**L3MBTL1:alt-TSS-DMR**	Yes	0.626019485	0.055168063	0.460515297	0.791523673	0.628138262	0.030647145	0.654590558	0.057099441	0.584312346	-0.013178771
20	42142495	cg23626798	**L3MBTL1:alt-TSS-DMR**	Yes	0.664582617	0.050421544	0.513317984	0.815847251	0.646319715	0.011898156	0.678498073	0.044076515	0.669615516	0.035193958
20	42142560	cg15388309	**L3MBTL1:alt-TSS-DMR**	Yes	0.749480289	0.042124504	0.623106778	0.8758538	0.705596629	-0.009581898	0.774471521	0.059292994	0.768881663	0.053703137
20	42142597	cg16862791	**L3MBTL1:alt-TSS-DMR**	Yes	0.555264121	0.035477748	0.448830877	0.661697365	0.561341752	0.03236532	0.5611057	0.032129268	0.53653341	0.007556978
20	42142672	cg06446163	**L3MBTL1:alt-TSS-DMR**	Yes	0.582450311	0.065693406	0.385370094	0.779530529	0.581018658	0.02605276	0.619364182	0.064398284	0.544487887	-0.010478011

20	42142674	cg04984575	**L3MBTL1:alt-TSS-DMR**	Yes	0.618430297	0.051541541	0.463805674	0.77305492	0.618578262	0.028026983	0.625975177	0.035423898	0.609605293	0.019054013
20	42142700	cg02177317	**L3MBTL1:alt-TSS-DMR**	Yes	0.802211346	0.024340554	0.729189685	0.875233007	0.786372433	0.019630717	0.819569574	0.052827859	0.80229571	0.035553994
20	42142752	cg20529070	**L3MBTL1:alt-TSS-DMR**	Yes	0.627668487	0.060934762	0.444864201	0.810472772	0.602752345	0.005245324	0.673146339	0.075639318	0.622038431	0.02453141
20	42142767	cg10360552	**L3MBTL1:alt-TSS-DMR**	Yes	0.622311945	0.031887079	0.526650707	0.717973183	0.594893328	3.57239E-05	0.637800134	0.04294253	0.631881037	0.037023433
20	42142785	cg18911945	**L3MBTL1:alt-TSS-DMR**	NA	0.538779678	0.03243285	0.441481129	0.636078228	0.502716433	-0.014109199	0.595892859	0.079067228	0.495864383	-0.020961249
20	42142848	cg01877937	**L3MBTL1:alt-TSS-DMR**	Yes	0.602742868	0.0649951	0.407757567	0.797728169	0.592349959	0.019469559	0.620731634	0.047851234	0.605077611	0.032197211
20	42142853	cg08633313	**L3MBTL1:alt-TSS-DMR**	Yes	0.604953756	0.062665959	0.41695588	0.792951633	0.631143565	0.054997446	0.640192019	0.0640459	0.55498318	-0.021162938
20	42142898	cg12699433	**L3MBTL1:alt-TSS-DMR**	Yes	0.563839828	0.066615562	0.363993143	0.763686512	0.536329578	0.001341901	0.611932006	0.07694433	0.542659027	0.007671351
20	42142948	cg11319028	**L3MBTL1:alt-TSS-DMR**	Yes	0.547662603	0.067735254	0.34445684	0.750868366	0.583357172	0.062186426	0.550916408	0.029745662	0.520205824	-0.000964923
20	42142996	cg22601123	**L3MBTL1:alt-TSS-DMR**	Yes	0.626907442	0.065575423	0.430181172	0.823633712	0.685456535	0.085840493	0.579667194	-0.019948848	0.608664259	0.009048217
20	42143016	cg22330467	**L3MBTL1:alt-TSS-DMR**	Yes	0.661137304	0.052476586	0.503707547	0.818567061	0.665284398	0.033756497	0.703290591	0.07176269	0.646630036	0.015102135
20	42143046	cg14306330	**L3MBTL1:alt-TSS-DMR**	Yes	0.496912955	0.074547796	0.273269567	0.720556344	0.528815478	0.056178957	0.455575255	-0.017061266	0.50855746	0.035920939
20	42143081	cg01071811	**L3MBTL1:alt-TSS-DMR**	Yes	0.695998426	0.047498825	0.553501951	0.838494901	0.674089421	0.010521875	0.752001779	0.088434233	0.648726071	-0.014841476
20	42143097	cg02611863	**L3MBTL1:alt-TSS-DMR**	Yes	0.677168114	0.052722212	0.519001478	0.83533475	0.687138288	0.041912563	0.694131934	0.048906209	0.658742511	0.013516786
20	42143175	cg15330298	**L3MBTL1:alt-TSS-DMR**	Yes	0.717329539	0.057034031	0.546227447	0.888431632	0.728798611	0.043645393	0.770432906	0.085279687	0.674279254	-0.010873964
20	42143212	cg20252111	**L3MBTL1:alt-TSS-DMR**	Yes	0.629100936	0.027673164	0.546081445	0.712120427	0.637278943	0.034535147	0.645361935	0.042618139	0.597553283	-0.005190513
20	42143400	cg15302378	**L3MBTL1:alt-TSS-DMR**	Yes	0.733172804	0.036585184	0.623417252	0.842928357	0.753982627	0.052221708	0.729543355	0.027782437	0.726655412	0.024894493
20	42143490	cg09541000	**L3MBTL1:alt-TSS-DMR**	Yes	0.718545802	0.049149252	0.571098047	0.865993557	0.679249659	-0.007029496	0.752636386	0.066357231	0.694232686	0.007953531
20	42143503	cg02472486	**L3MBTL1:alt-TSS-DMR**	Yes	0.563327834	0.03330344	0.463417513	0.663238155	0.5661901	0.027247152	0.589368899	0.050425951	0.527429403	-0.011513545
20	57414040	cg04132853	**GNAS-NESP:TSS-DMR**	Yes	0.695459431	0.040894227	0.572776749	0.818142113	0.668175554	0.005007207	0.710240705	0.047072357	0.683344332	0.020175984
20	57414060	cg25090051	**GNAS-NESP:TSS-DMR**	Yes	0.727333255	0.032140451	0.630911902	0.823754609	0.707948816	0.013097074	0.756113359	0.061261617	0.69358181	-0.001269931
20	57414163	cg00732970	**GNAS-NESP:TSS-DMR**	Yes	0.575852564	0.02747245	0.493435215	0.658269913	0.56029772	0.010971766	0.573852437	0.024526483	0.55424944	0.004923487
20	57414218	cg17696847	**GNAS-NESP:TSS-DMR**	Yes	0.644983944	0.046138056	0.506569777	0.783398111	0.608206186	-0.006474361	0.687750436	0.073069888	0.609364001	-0.005316547
20	57414352	cg20019489	**GNAS-NESP:TSS-DMR**	Yes	0.84219962	0.03073787	0.749986009	0.934413231	0.855044508	0.049615344	0.851588391	0.046159227	0.837052547	0.031623383
20	57414408	cg02274728	**GNAS-NESP:TSS-DMR**	Yes	0.682921755	0.042139398	0.556503562	0.809339947	0.66884152	0.016396462	0.687806618	0.035361559	0.680462651	0.028017593
20	57414530	cg12054318	**GNAS-NESP:TSS-DMR**	Yes	0.663031602	0.036769039	0.552724486	0.773338717	0.662356769	0.028434902	0.666576607	0.03265474	0.654941382	0.021019515
20	57414540	cg25268451	**GNAS-NESP:TSS-DMR**	Yes	0.707049071	0.026468482	0.627643623	0.786454518	0.724244718	0.048911573	0.713354589	0.038021443	0.69022888	0.014895734
20	57414579	cg11996914	**GNAS-NESP:TSS-DMR**	Yes	0.59394352	0.023524823	0.523369051	0.664517988	0.584026732	0.016115767	0.61424122	0.046330255	0.578507487	0.010596522
20	57414597	cg21330323	**GNAS-NESP:TSS-DMR**	NA	0.560915599	0.019855455	0.501349235	0.620481963	0.560889839	0.024468147	0.572472926	0.036051233	0.557639385	0.021217692
20	57414746	cg17986444	**GNAS-NESP:TSS-DMR**	NA	0.714440635	0.022084833	0.648186135	0.780695135	0.718721677	0.036346332	0.70897495	0.026599606	0.705592869	0.023217524
20	57414885	cg06163629	**GNAS-NESP:TSS-DMR**	Yes	0.563110688	0.025856066	0.485542489	0.640678887	0.574600556	0.033442403	0.562619427	0.021461274	0.548297438	0.007139284
20	57415145	cg00765653	**GNAS-NESP:TSS-DMR**	Yes	0.588309518	0.017787775	0.534946192	0.641672844	0.604707779	0.041752607	0.576009403	0.013054231	0.584997386	0.022042215
20	57415178	cg14728235	**GNAS-NESP:TSS-DMR**	Yes	0.642586017	0.020277183	0.581754469	0.703417565	0.635237821	0.019015862	0.655642697	0.039420738	0.647017147	0.030795188
20	57415378	cg05558390	**GNAS-NESP:TSS-DMR**	Yes	0.368023763	0.021340854	0.3040012	0.432046326	0.373953265	0.020501789	0.3594011	0.005949623	0.353509462	5.79854E-05
20	57415698	cg19296354	**GNAS-NESP:TSS-DMR**	Yes	0.617449505	0.041165731	0.493952313	0.740946698	0.615865596	0.026242669	0.611640377	0.02201745	0.638058431	0.048435504
20	57415956	cg15725616	**GNAS-NESP:TSS-DMR**	NA	0.610685089	0.04044094	0.489362267	0.73200791	0.597224235	0.015346851	0.56677463	-0.015102754	0.646908453	0.065031069
20	57415979	cg18035618	**GNAS-NESP:TSS-DMR**	NA	0.688933607	0.033694666	0.587849608	0.790017605	0.675267516	0.016896342	0.683564886	0.025193712	0.712278394	0.053907219
20	57416222	cg24975842	**GNAS-NESP:TSS-DMR**	Yes	0.546348344	0.025917409	0.468596117	0.624100571	0.562445603	0.039065009	0.541268587	0.017887992	0.540237789	0.016857194
20	57416507	cg18619398	**GNAS-NESP:TSS-DMR**	Yes	0.56236912	0.03908367	0.445118108	0.679620131	0.542795989	0.006072519	0.544921904	0.008198434	0.583494533	0.046771064
20	57416889	cg01355739	**GNAS-NESP:TSS-DMR**	Yes	0.676001475	0.030492832	0.584522978	0.767479972	0.669564883	0.024285535	0.675416611	0.030137262	0.68361672	0.038337371
20	57417234	cg20582984	**GNAS-NESP:TSS-DMR**	Yes	0.775905959	0.029791681	0.686530916	0.865281002	0.766506733	0.024934587	0.787458461	0.045886314	0.786049334	0.044477187
20	57418016	cg18949315	**GNAS-NESP:TSS-DMR**	Yes	0.611087925	0.041567641	0.486385001	0.735790848	0.617823789	0.035212525	0.626979456	0.044368192	0.585532663	0.002921399
20	57418374	cg02544157	**GNAS-NESP:TSS-DMR**	NA	0.473799978	0.033168708	0.374293855	0.573306102	0.485066892	0.033915724	0.464309968	0.0131588	0.467007181	0.015856014
20	57425980	cg18935491	**GNAS-AS1:TSS-DMR**	Yes	0.687087114	0.024603454	0.613276751	0.760897478	0.651705308	-0.006479937	0.674790673	0.016605428	0.706656986	0.048471742
20	57425987	cg24203465	**GNAS-AS1:TSS-DMR**	Yes	0.59608806	0.035687416	0.489025812	0.703150308	0.566286484	-0.002986663	0.608298784	0.039025637	0.61261956	0.043346412
20	57425995	cg26102503	**GNAS-AS1:TSS-DMR**	Yes	0.636676202	0.040485003	0.515221192	0.758131212	0.57170983	-0.035215686	0.651773862	0.044848346	0.658846105	0.051920589
20	57426132	cg03344105	**GNAS-AS1:TSS-DMR**	Yes	0.653745138	0.032629463	0.555856748	0.751633528	0.632438547	0.007933766	0.68171735	0.057212568	0.626138523	0.001633741
20	57426139	cg14660776	**GNAS-AS1:TSS-DMR**	Yes	0.550345236	0.03730164	0.438440317	0.662250156	0.531830556	0.007086151	0.552071234	0.027326829	0.55962724	0.034882835
20	57426216	cg19592829	**GNAS-AS1:TSS-DMR**	Yes	0.751812584	0.024200217	0.679211932	0.824413236	0.742870935	0.023681024	0.747754589	0.028564678	0.761484958	0.042295048
20	57426265	cg09123158	**GNAS-AS1:TSS-DMR**	Yes	0.81627117	0.034573479	0.712550732	0.919991608	0.774963675	-0.005598178	0.868933839	0.088371985	0.806912966	0.026351113
20	57426275	cg19140375	**GNAS-AS1:TSS-DMR**	NA	0.314102867	0.061376919	0.129972109	0.498233625	0.250606395	-0.048401598	0.394202986	0.095194993	0.300858894	0.0018509
20	57426323	cg11669839	**GNAS-AS1:TSS-DMR**	Yes	0.505042235	0.042066601	0.378842432	0.631242038	0.475801362	-0.005400674	0.475928841	-0.005273195	0.528029885	0.046827848
20	57426369	cg17841572	**GNAS-AS1:TSS-DMR**	Yes	0.598256315	0.032228635	0.50157041	0.69494222	0.549273615	-0.022704176	0.63664443	0.064666639	0.614208178	0.042230386
20	57426375	cg25229306	**GNAS-AS1:TSS-DMR**	Yes	0.64864165	0.030737248	0.556429906	0.740853393	0.635300881	0.015014845	0.665191353	0.044905317	0.660918528	0.040632492
20	57426384	cg14482474	**GNAS-AS1:TSS-DMR**	Yes	0.638619036	0.032956511	0.539749504	0.737488569	0.603295538	-0.007056859	0.636824039	0.026471643	0.662314478	0.051962082
20	57426392	cg08166863	**GNAS-AS1:TSS-DMR**	Yes	0.646847322	0.024267267	0.574045522	0.719649123	0.641206317	0.020935913	0.673259281	0.052988877	0.629715541	0.009445137
20	57426396	cg11285807	**GNAS-AS1:TSS-DMR**	Yes	0.606145816	0.021138131	0.542731424	0.669560208	0.591869609	0.011480862	0.60526636	0.024877613	0.613157082	0.032768335
20	57426421	cg06200857	**GNAS-AS1:TSS-DMR**	Yes	0.600245067	0.038689772	0.484175751	0.716314383	0.536459018	-0.035477535	0.602961619	0.031025065	0.63830551	0.066368957
20	57426426	cg08091561	**GNAS-AS1:TSS-DMR**	Yes	0.62721263	0.037386679	0.515052594	0.739372667	0.549496685	-0.048722174	0.64731466	0.0490958	0.65875063	0.06053177
20	57426539	cg22860367	**GNAS-AS1:TSS-DMR**	Yes	0.536426459	0.041301503	0.412521949	0.660330968	0.458056511	-0.054249075	0.536356931	0.024051345	0.491610414	-0.020695172
20	57426546	cg07947033	**GNAS-AS1:TSS-DMR**	Yes	0.626649587	0.02209578	0.560362249	0.692936926	0.598059275	-0.001407871	0.624735917	0.025268771	0.643824389	0.044357243
20	57426571	cg06693667	**GNAS-AS1:TSS-DMR**	Yes	0.605764817	0.035975723	0.497837647	0.713691986	0.575980541	-0.001427304	0.606660972	0.029253127	0.626535712	0.049127867
20	57426744	cg04677683	**GNAS-AS1:TSS-DMR**	Yes	0.612841423	0.04123896	0.489124543	0.736558304	0.658356894	0.073080565	0.533894949	-0.05138138	0.638655419	0.05337909
20	57426750	cg15160445	**GNAS-AS1:TSS-DMR**	Yes	0.660971534	0.03903141	0.543877304	0.778065764	0.705829491	0.074765668	0.588072883	-0.042990941	0.682282982	0.051219158
20	57426758	cg25326570	**GNAS-AS1:TSS-DMR**	Yes	0.654944649	0.035272082	0.549128403	0.760760894	0.680320292	0.054431427	0.58261874	-0.043270124	0.660321527	0.034432663
20	57426760	cg23249369	**GNAS-AS1:TSS-DMR**	Yes	0.713188392	0.016759672	0.662909375	0.763467409	0.712224118	0.02872748	0.676793377	-0.006703261	0.729965134	0.046468496
20	57426790	cg01715551	**GNAS-AS1:TSS-DMR**	Yes	0.550959177	0.04094466	0.428125197	0.673793158	0.508165683	-0.016810968	0.518857928	-0.006118723	0.573918766	0.048942115
20	57426802	cg14176797	**GNAS-AS1:TSS-DMR**	Yes	0.592855576	0.042776894	0.464524894	0.721186258	0.531848057	-0.033044273	0.572415785	0.007523455	0.644128311	0.079235981
20	57426836	cg04457481	**GNAS-AS1:TSS-DMR**	Yes	0.607345712	0.041299606	0.483446894	0.731244529	0.571061296	-0.008765356	0.59262182	0.012795168	0.620499591	0.040672939
20	57426859	cg27262796	**GNAS-AS1:TSS-DMR**	Yes	0.631795418	0.027440089	0.549475152	0.714115684	0.588716715	-0.015030622	0.60402513	0.000277793	0.640818444	0.037071107
20	57426932	cg21938532	**GNAS-AS1:TSS-DMR**	Yes	0.648643455	0.023821708	0.577178331	0.72010858	0.599778775	-0.021000521	0.634337242	0.013557945	0.668926049	0.048146752
20	57426951	cg22989942	**GNAS-AS1:TSS-DMR**	Yes	0.605496375	0.017879576	0.551857646	0.659135103	0.583065404	0.003938121	0.60003429	0.020907007	0.615613398	0.036486114
20	57426980	cg18224653	**GNAS-AS1:TSS-DMR**	Yes	0.545381552	0.020099453	0.485083194	0.60567991	0.499312951	-0.023644758	0.553191093	0.030233383	0.548320445	0.025362736
20	57427011	cg14309385	**GNAS-AS1:TSS-DMR**	Yes	0.574389161	0.014026379	0.532310023	0.616468299	0.553186024	0.002580086	0.580901661	0.030295723	0.578888256	0.028282319
20	57427018	cg05309239	**GNAS-AS1:TSS-DMR**	Yes	0.586953196	0.023119509	0.517594669	0.656311723	0.528848993	-0.032898068	0.608427336	0.046680275	0.583160164	0.021413103
20	57427031	cg12573482	**GNAS-AS1:TSS-DMR**	Yes	0.510370907	0.035320008	0.404410883	0.61633093	0.44810474	-0.038186348	0.489206989	0.002915901	0.550125081	0.063833992
20	57427104	cg06324048	**GNAS-AS1:TSS-DMR**	Yes	0.630320644	0.052371342	0.473206619	0.787434668	0.535518977	-0.068473203	0.577463047	-0.026529133	0.681879836	0.077887655
20	57427147	cg24617313	**GNAS-AS1:TSS-DMR**	Yes	0.556888838	0.06341313	0.366649449	0.747128226	0.668743825	0.134147526	0.356849855	-0.177746444	0.566121905	0.031525605
20	57427171	cg03908391	**GNAS-AS1:TSS-DMR**	Yes	0.619175968	0.034208902	0.516549262	0.721802674	0.497855976	-0.097839289	0.627167031	0.031471766	0.568615708	-0.027079557
20	57427174	cg22290117	**GNAS-AS1:TSS-DMR**	Yes	0.657136399	0.046008096	0.51911211	0.795160688	0.511395771	-0.118052264	0.667186642	0.037738607	0.655833478	0.026385443
20	57427211	cg26496204	**GNAS-AS1:TSS-DMR**	Yes	0.614158878	0.041986696	0.488198791	0.740118965	0.483719621	-0.103808348	0.609691989	0.02216402	0.63457869	0.047050722
20	57427275	cg03010274	**GNAS-AS1:TSS-DMR**	Yes	0.505852547	0.046245282	0.3671167	0.644588393	0.389317156	-0.093444858	0.501599955	0.018837941	0.507892709	0.025130695
20	57427413	cg25652859	**GNAS-AS1:TSS-DMR**	Yes	0.384140916	0.057403494	0.211930435	0.556351398	0.239860779	-0.129663282	0.348498726	-0.021025335	0.40011385	0.030589789
20	57427427	cg12321149	**GNAS-AS1:TSS-DMR**	Yes	0.468774167	0.068022861	0.264705583	0.672842751	0.273913861	-0.178220886	0.457294775	0.005160029	0.431295064	-0.020839683
20	57427444	cg06065549	**GNAS-AS1:TSS-DMR**	Yes	0.471173429	0.038172804	0.356655016	0.585691842	0.354596846	-0.096591641	0.445638271	-0.005550215	0.434631605	-0.016556882
20	57427473	cg07105596	**GNAS-AS1:TSS-DMR**	Yes	0.455624555	0.068613597	0.249783765	0.661465345	0.25777425	-0.184361701	0.420788747	-0.021347204	0.421720495	-0.020415456
20	57427484	cg27304369	**GNAS-AS1:TSS-DMR**	Yes	0.530751174	0.054750054	0.366501013	0.695001335	0.387746073	-0.126522091	0.53593829	0.021670126	0.52808428	0.013816116
20	57427494	cg26811638	**GNAS-AS1:TSS-DMR**	Yes	0.35761204	0.051947154	0.201770578	0.513453501	0.234438866	-0.114246576	0.384261424	0.035575981	0.34250019	-0.006185253
20	57427496	cg26534489	**GNAS-AS1:TSS-DMR**	Yes	0.457015725	0.041207316	0.333393777	0.580637673	0.374703584	-0.067083717	0.473776559	0.031989257	0.440232814	-0.001554488
20	57427504	cg08587534	**GNAS-AS1:TSS-DMR**	Yes	0.475422079	0.036687086	0.365360821	0.585483336	0.382187933	-0.076050141	0.44495457	-0.013283504	0.499637901	0.041399826
20	57427557	cg14564778	**GNAS-AS1:TSS-DMR**	Yes	0.438300846	0.061180931	0.254758053	0.621843638	0.286020779	-0.139785149	0.447847765	0.022041838	0.36046917	-0.065336757
20	57427643	cg01817393	**GNAS-AS1:TSS-DMR**	Yes	0.350798711	0.075732621	0.123600848	0.577996574	0.19329688	-0.143016229	0.243871356	-0.092441753	0.279488852	-0.056824257
20	57427651	cg02890368	**GNAS-AS1:TSS-DMR**	Yes	0.26615916	0.077085639	0.034902244	0.497416076	0.14223029	-0.116419904	0.313916374	0.05526618	0.245048247	-0.013601948
20	57427714	cg04257105	**GNAS-AS1:TSS-DMR**	Yes	0.426526574	0.053793177	0.265147044	0.587906104	0.256672369	-0.157889986	0.414671689	0.000109334	0.344050588	-0.070511768
20	57427731	cg20528838	**GNAS-AS1:TSS-DMR**	Yes	0.278423187	0.052999351	0.119425135	0.437421239	0.128024924	-0.143259365	0.328355875	0.057071586	0.229542094	-0.041742195
20	57427739	cg27661264	**GNAS-AS1:TSS-DMR**	Yes	0.31655774	0.049184712	0.169003604	0.464111876	0.157477666	-0.147361192	0.322480963	0.017642105	0.250379829	-0.054459029
20	57427763	cg19589727	**GNAS-AS1:TSS-DMR**	Yes	0.348991946	0.061552639	0.16433403	0.533649862	0.169155821	-0.167131338	0.345914367	0.009627208	0.27118984	-0.065097319
20	57427822	cg10302550	**GNAS-AS1:TSS-DMR**	Yes	0.460104848	0.056667488	0.290102386	0.630107311	0.248876265	-0.197084196	0.460941304	0.014980843	0.383370412	-0.062590049
20	57427831	cg17414107	**GNAS-AS1:TSS-DMR**	Yes	0.594142187	0.076407893	0.364918509	0.823365865	0.331956293	-0.241873954	0.591075617	0.01724537	0.500512416	-0.073317831
20	57427943	cg00943909	**GNAS-AS1:TSS-DMR**	Yes	0.469075372	0.054666025	0.305077297	0.633073448	0.228814678	-0.226118389	0.499540044	0.044606977	0.402212764	-0.052720303
20	57427974	cg19640589	**GNAS-AS1:TSS-DMR**	Yes	0.311222832	0.054868706	0.146616713	0.47582895	0.125046515	-0.178382608	0.30319388	-0.000235243	0.253929087	-0.049500036
20	57427978	cg09393900	**GNAS-AS1:TSS-DMR**	NA	0.509427251	0.054243536	0.346696643	0.672157859	0.322303998	-0.171564884	0.508793707	0.014924825	0.447761712	-0.046107169
20	57429026	cg20554943	**GNAS-XL:Ex1-DMR**	NA	0.476189085	0.033163054	0.376699924	0.575678245	0.472367042	0.015850332	0.443935446	-0.012581263	0.511786757	0.055270048
20	57429859	cg07284407	**GNAS-XL:Ex1-DMR**	Yes	0.581200753	0.040605977	0.459382822	0.703018684	0.583083443	0.028458601	0.574736786	0.020111945	0.576966672	0.02234183
20	57430134	cg26711395	**GNAS-XL:Ex1-DMR**	NA	0.680484546	0.013449425	0.640136271	0.720832821	0.695915416	0.044035104	0.675199435	0.023319123	0.65829304	0.006412728
20	57430314	cg21625881	**GNAS-XL:Ex1-DMR**	Yes	0.74098829	0.036367432	0.631885995	0.850090585	0.729621011	0.021128318	0.750707503	0.04221481	0.716690508	0.008197815
20	57430664	cg14203179	**GNAS-XL:Ex1-DMR**	Yes	0.559804208	0.024139733	0.48738501	0.632223405	0.567867953	0.032399853	0.529220697	-0.006247403	0.584845136	0.049377035
20	57431203	cg09437522	**GNAS-XL:Ex1-DMR**	Yes	0.593777459	0.036872669	0.483159453	0.704395465	0.64964449	0.082809989	0.553072758	-0.013761743	0.592763429	0.025928928
20	57431304	cg10468484	**GNAS-XL:Ex1-DMR**	Yes	0.577096455	0.02134157	0.513071746	0.641121165	0.557594119	0.003008625	0.599716448	0.045130955	0.567422661	0.012837168
20	57463266	cg22741626	**GNAS-A/B:TSS-DMR**	Yes	0.845102819	0.026364631	0.766008927	0.924196712	0.841710356	0.033989541	0.863606501	0.055885687	0.836315553	0.028594739
20	57463271	cg18997188	**GNAS-A/B:TSS-DMR**	Yes	0.771363504	0.032768094	0.673059221	0.869667786	0.766373323	0.030386529	0.819465449	0.083478655	0.732535622	-0.003451172
20	57463326	cg20213508	**GNAS-A/B:TSS-DMR**	Yes	0.830148461	0.033542765	0.729520166	0.930776756	0.81870328	0.025299549	0.865829715	0.072425985	0.811477028	0.018073298
20	57463331	cg26791489	**GNAS-A/B:TSS-DMR**	Yes	0.766467322	0.031428429	0.672182034	0.86075261	0.733671827	0.001560418	0.799829387	0.067717979	0.757456597	0.025345189
20	57463356	cg04779428	**GNAS-A/B:TSS-DMR**	Yes	0.80494425	0.034014561	0.702900566	0.906987933	0.791298863	0.022174135	0.827845893	0.058721165	0.800957406	0.031832677
20	57463358	cg04019914	**GNAS-A/B:TSS-DMR**	Yes	0.85019553	0.029886437	0.760536217	0.939854842	0.8280999	0.01546579	0.868904736	0.056270626	0.837703459	0.025069349
20	57463398	cg11357538	**GNAS-A/B:TSS-DMR**	Yes	0.659253503	0.039370667	0.541141502	0.777365504	0.650239607	0.020568113	0.661258004	0.03158651	0.655955973	0.026284479

20	57463456	cg20008140	**GNAS-A/B:TSS-DMR**	Yes	0.664602471	0.047937623	0.520789603	0.80841534	0.624056313	-0.010267509	0.651615448	0.017291626	0.687370926	0.053047104
20	57463504	cg11480267	**GNAS-A/B:TSS-DMR**	Yes	0.630934235	0.075757673	0.403661216	0.858207255	0.686434613	0.086006781	0.499038187	-0.101389645	0.681599934	0.081172102
20	57463528	cg10011623	**GNAS-A/B:TSS-DMR**	Yes	0.867350062	0.022407814	0.800126621	0.934573502	0.864228536	0.033750236	0.888475479	0.057997179	0.848485687	0.018007387
20	57463531	cg01748573	**GNAS-A/B:TSS-DMR**	Yes	0.601805382	0.028513549	0.516264735	0.687346029	0.570246269	-0.005337131	0.600218953	0.024635552	0.618851486	0.043268085
20	57463573	cg17334845	**GNAS-A/B:TSS-DMR**	Yes	0.536661076	0.042888577	0.407995345	0.665326807	0.538599464	0.027971246	0.517162409	0.006534191	0.542100343	0.031472124
20	57463616	cg26767990	**GNAS-A/B:TSS-DMR**	Yes	0.537638873	0.03483059	0.433147103	0.642130643	0.569387256	0.055388763	0.528887784	0.01488929	0.544530305	0.030531811
20	57463654	cg17652507	**GNAS-A/B:TSS-DMR**	Yes	0.566560754	0.034235634	0.46385385	0.669267657	0.586716295	0.045211003	0.54764757	0.006142278	0.576684959	0.035179667
20	57463659	cg22407822	**GNAS-A/B:TSS-DMR**	Yes	0.639615499	0.035479615	0.533176654	0.746054343	0.628871882	0.014497089	0.627707325	0.013332532	0.667219498	0.052844705
20	57463712	cg07341934	**GNAS-A/B:TSS-DMR**	Yes	0.631005868	0.040826029	0.508527781	0.753483954	0.592474694	-0.010109649	0.640107481	0.037523138	0.638950186	0.036365843
20	57463714	cg02107718	**GNAS-A/B:TSS-DMR**	Yes	0.600156542	0.044682283	0.466109694	0.73420339	0.566733258	-0.005567079	0.605976776	0.033676439	0.612846718	0.04054638
20	57463726	cg23496597	**GNAS-A/B:TSS-DMR**	Yes	0.553435075	0.024404604	0.480221263	0.626648887	0.559213989	0.027944841	0.548519788	0.017250639	0.563659441	0.032390292
20	57463764	cg25308079	**GNAS-A/B:TSS-DMR**	Yes	0.648081092	0.01974555	0.588844443	0.707317741	0.658474707	0.037273122	0.642916847	0.021715262	0.636041191	0.014839605
20	57463776	cg09772382	**GNAS-A/B:TSS-DMR**	Yes	0.66502618	0.019254207	0.607263559	0.722788802	0.651018389	0.014544774	0.639741343	0.003267728	0.690515792	0.054042177
20	57463784	cg15222215	**GNAS-A/B:TSS-DMR**	Yes	0.598484151	0.020580159	0.536743673	0.660224629	0.614467721	0.041295817	0.581500143	0.00832824	0.596620319	0.023448416
20	57463788	cg14263118	**GNAS-A/B:TSS-DMR**	Yes	0.601989726	0.020683443	0.539939396	0.664040057	0.586527575	0.009393478	0.619712386	0.04257829	0.598345855	0.021211759
20	57463901	cg11244758	**GNAS-A/B:TSS-DMR**	Yes	0.584443472	0.021982292	0.518496597	0.650390348	0.582843369	0.023940165	0.578967392	0.020064188	0.601191738	0.042288534
20	57463907	cg05926269	**GNAS-A/B:TSS-DMR**	Yes	0.773655271	0.026868016	0.693051225	0.854259318	0.779192916	0.038654158	0.775245449	0.034706691	0.767069152	0.026530394
20	57463926	cg03821543	**GNAS-A/B:TSS-DMR**	Yes	0.480551257	0.031948702	0.384705152	0.576397362	0.471792317	0.013041225	0.486863433	0.028112342	0.510941921	0.052190829
20	57463975	cg01538522	**GNAS-A/B:TSS-DMR**	Yes	0.451695757	0.046759413	0.311417517	0.591973997	0.466488154	0.037507105	0.406621509	-0.02235954	0.473694739	0.04471369
20	57463985	cg00267746	**GNAS-A/B:TSS-DMR**	Yes	0.475924634	0.027780419	0.392583378	0.559265889	0.472287384	0.01750877	0.488711856	0.033933242	0.466748664	0.01197005
20	57464001	cg03837903	**GNAS-A/B:TSS-DMR**	Yes	0.556024819	0.064709403	0.361896609	0.750153029	0.594891359	0.065664239	0.454823493	-0.074403627	0.615258293	0.086031173
20	57464003	cg23159236	**GNAS-A/B:TSS-DMR**	Yes	0.413919167	0.029512473	0.325381747	0.502456587	0.422102994	0.025738289	0.371998526	-0.02436618	0.437117166	0.040752461
20	57464130	cg22798925	**GNAS-A/B:TSS-DMR**	Yes	0.538786449	0.027966919	0.454885694	0.622687205	0.52142282	0.005622078	0.529725757	0.013925015	0.551379945	0.035579203
20	57464572	cg20126878	**GNAS-A/B:TSS-DMR**	Yes	0.565542136	0.040878962	0.442905249	0.688179022	0.548839565	0.00962725	0.548147941	0.008935626	0.580110094	0.040897778
20	57464743	cg27027803	**GNAS-A/B:TSS-DMR**	Yes	0.662595908	0.039503128	0.544086525	0.781105292	0.649060117	0.017022715	0.658271208	0.026233806	0.686664719	0.054627318
20	57464971	cg08997444	**GNAS-A/B:TSS-DMR**	Yes	0.74107729	0.031657441	0.646104966	0.836049613	0.721697946	0.013825254	0.723812293	0.0159396	0.76255655	0.054683857
20	57464974	cg22639787	**GNAS-A/B:TSS-DMR**	Yes	0.726015138	0.02754255	0.643387487	0.808642789	0.680953813	-0.012661013	0.745079687	0.051464861	0.733003406	0.039388579
20	57465124	cg05960039	**GNAS-A/B:TSS-DMR**	Yes	0.362876016	0.026644555	0.282942351	0.442809681	0.366046789	0.019333405	0.369418627	0.022705243	0.332377818	-0.014335566
20	57465126	cg09604333	**GNAS-A/B:TSS-DMR**	Yes	0.452138529	0.023077855	0.382904965	0.521372093	0.448177477	0.015847928	0.422758366	-0.009571183	0.470972702	0.038643153
20	57465133	cg06047881	**GNAS-A/B:TSS-DMR**	Yes	0.544120868	0.045315028	0.408175784	0.680065952	0.551235095	0.031337634	0.524315033	0.004417572	0.567540112	0.04764265
20	57465140	cg20018057	**GNAS-A/B:TSS-DMR**	Yes	0.52201858	0.047077437	0.380786269	0.66325089	0.544233299	0.045558698	0.470236584	-0.028438017	0.533341934	0.034667333
20	57465176	cg10748817	**GNAS-A/B:TSS-DMR**	Yes	0.579010419	0.028169527	0.494501837	0.663519	0.555123097	-0.001089257	0.58757879	0.031366436	0.606940197	0.050727842
21	15352609	cg00566515	ANKRD20A11P	Yes	0.294193795	0.09558584	0.007436275	0.580951316	0.445781356	0.160553196	0.231253865	-0.053974294	0.347471322	0.062243162
21	15352849	cg02197629	ANKRD20A11P	Yes	0.388582627	0.136907375	-0.022139498	0.799304753	0.711271152	0.332154159	0.348155501	-0.030961492	0.331622708	-0.047494285
21	15436261	cg25507885	LIPI	Yes	0.858512196	0.025103828	0.783200711	0.933823681	0.876554155	0.055981164	0.876889892	0.056316901	0.863227109	0.042654118
21	15456606	cg19509405	LIPI	Yes	0.991560837	0.003107619	0.982237979	1.000883694	0.991584852	0.041366073	0.990497187	0.040278408	0.99561874	0.04539996
21	15457267	cg24114772	LIPI	Yes	0.978512119	0.003707508	0.967389596	0.989634642	0.981476881	0.043853473	0.978913897	0.041290489	0.977692584	0.040069176
21	40757692	cg00606841	**WRB:alt-TSS-DMR**	Yes	0.671455348	0.049243202	0.523725742	0.819184954	0.60029097	-0.039703013	0.69488256	0.054888578	0.70174356	0.061749578
21	40757900	cg26710963	**WRB:alt-TSS-DMR**	Yes	0.55027174	0.044604284	0.416458889	0.684084591	0.522390648	-0.000724579	0.574154414	0.051039187	0.55825257	0.035137343
21	40758209	cg09916765	**WRB:alt-TSS-DMR**	Yes	0.351325964	0.084025072	0.099250747	0.603401181	0.376594401	0.040175846	0.406221633	0.069803077	0.249018684	-0.087399872
22	42077940	cg06781532	**SNU13:alt-TSS-DMR**	Yes	0.745434717	0.040246784	0.624694366	0.866175067	0.761821	0.047675372	0.733011084	0.018865456	0.774276926	0.060131298
22	42078218	cg18152773	**SNU13:alt-TSS-DMR**	Yes	0.483397619	0.051482852	0.328949062	0.637846177	0.459014181	-0.001627292	0.455320115	-0.005321359	0.52733809	0.066696617
22	42078331	cg05871614	**SNU13:alt-TSS-DMR**	Yes	0.586945538	0.059696579	0.407855802	0.766035274	0.584685701	0.024662841	0.578473013	0.018450153	0.588315613	0.028292753
22	42078366	cg22083753	**SNU13:alt-TSS-DMR**	Yes	0.536006223	0.057957854	0.36213266	0.709879785	0.491772575	-0.019145863	0.527342388	0.01642395	0.595814744	0.084896306
22	42078389	cg15284719	**SNU13:alt-TSS-DMR**	Yes	0.500105079	0.051468703	0.345698969	0.65451119	0.484964526	0.007152359	0.508601238	0.030789071	0.513921552	0.036109385
22	42078568	cg08686092	**SNU13:alt-TSS-DMR**	Yes	0.523900565	0.057983443	0.349950237	0.697850893	0.530387508	0.029963968	0.528025837	0.027602296	0.508761621	0.008338081
22	42078708	cg11677105	**SNU13:alt-TSS-DMR**	Yes	0.411047251	0.072603294	0.193237368	0.628857134	0.376576105	-0.017176314	0.411627079	0.01787466	0.404867605	0.011115187

DMR, Differentially methylated region; 450k, Illumina 450K BeadChip Arrays; and SD, standard diviation.
The names of DMRs are based on the previous reports by Monk et al. [1] and Joshi et al. [2].
DMRs shown in bold letters are described in Monk et al. or in both Monk et al. and Joshi et al., and those shown in plain letters are described in Joshi et al. but not in Monk et al.
Beta values < mean - 3 SD and > mean + 3 SD are written in green and red numbers, respectively.
Delta beta values < 0.05 and > 0.05 are highlighted with green and red backgroundeds, respectively.
The probes with age-related drift and sex-bias have been excluded.

References

1. Monk D, Morales J, den Dunnen JT, Russo S, Court F, Prawitt D, et al. Recommendations for a nomenclature system for reporting methylation aberrations in imprinted domains. Epigenetics. 2018;13:117–21.
2. Joshi RS, Garg P, Zaitlen N, Lappalainen T, Watson CT, Azam N, et al. DNA Methylation Profiling of Uniparental Disomy Subjects Provides a Map of Parental Epigenetic Bias in the Human Genome. Am J Hum Genet. 2016;99:555–66.
