# Supplementary material for: ZNF445: a homozygous truncating variant in a patient with Temple syndrome and multilocus imprinting disturbance
Source: Clin Epigenetics. 2021 May 26;13:119. doi: 10.1186/s13148-021-01106-5 (PMC8157728; doi:10.1186/s13148-021-01106-5)
Supplement: Supplementary file 8 — Additional file 8: Figure S3. Physical map of the chromosome 14q32.2 imprinted region and that of the magnified MEG3/DLK1:IG-DMR and MEG3:TSS-DMR. [file 13148_2021_1106_MOESM8_ESM.pdf]

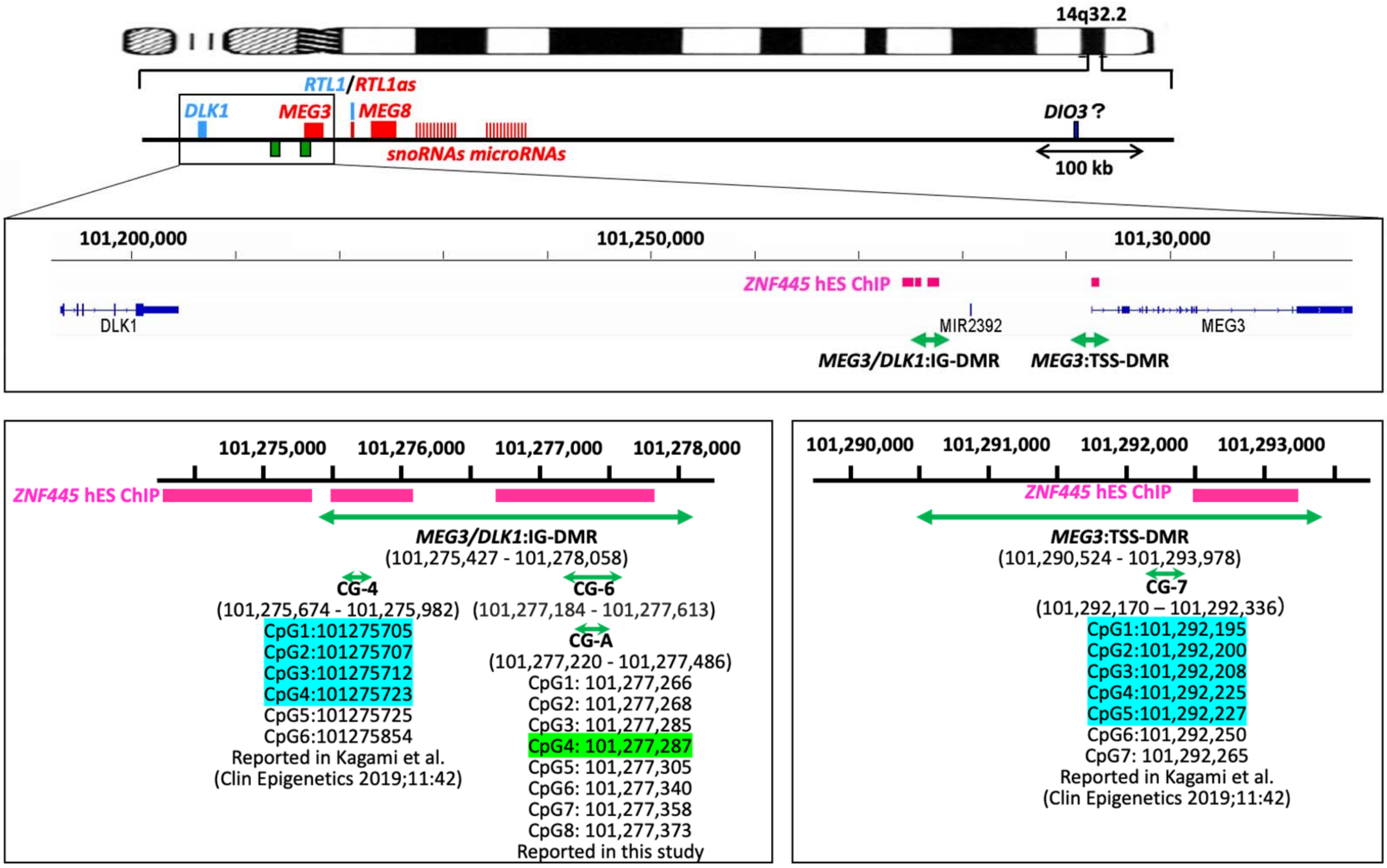

Figure S3. Physical map of the chromosome 14q32.2 imprinted region and that of the magnified *MEG3/DLK1*:IG-DMR and *MEG3*:TSS-DMR. The physical positions are based on GRCh37/h19. In the top panel, paternally and maternally expressed genes are shown in blue and red, respectively; a probably non-imprinted gene *DIO3* is depicted in black. In the middle and bottom panels, the pink bars represent the regions containing ZNF445 binding sites indicated by ChIP assay for hES (Integrative Genomics Viewer images of GSE115387_hES_445_HA_ChIP_S2_vs_hES_445_Ti_S1_peaks.bed.gz downloaded https://www.ncbi.nlm.nih.gov/geo/query/acc.cgi?acc=). The CG-4 and CG-7 have been studied by bisulfite sequencing in the previous paper (Kagami et al. [1]), and the CG-A has been investigated by bisulfite sequencing in this study. The CpGs1–4 at CG-4 and the CpGs1–5 at CG-7 highlighted with blue have also been examined by pyrosequencing (Table S1 of this study and Figure 2A of Kagami et al. [1]), and the CpG4 at CG-A highlighted with green has also been studied by Infinium MethylationEPIC in this study (Figure 2A in this report).

Reference

1. Kagami M, Yanagisawa A, Ota M, Matsuoka K, Nakamura A, Matsubara K, et al. Temple syndrome in a patient with variably methylated CpGs at the primary MEG3/*DLK1*:IG-DMR and severely hypomethylated CpGs at the secondary *MEG3*:TSS-DMR. Clin Epigenetics. 2019;11:42.
